# Unique genetic signatures of local adaptation over space and time for diapause, an ecologically relevant complex trait, in *Drosophila melanogaster*

**DOI:** 10.1371/journal.pgen.1009110

**Published:** 2020-11-20

**Authors:** Priscilla A. Erickson, Cory A. Weller, Daniel Y. Song, Alyssa S. Bangerter, Paul Schmidt, Alan O. Bergland

**Affiliations:** 1 Department of Biology, University of Virginia, Charlottesville, Virginia, United States of America; 2 Department of Biology, University of Pennsylvania, Philadelphia, Pennsylvania, United States of America; University of Oregon, UNITED STATES

## Abstract

Organisms living in seasonally variable environments utilize cues such as light and temperature to induce plastic responses, enabling them to exploit favorable seasons and avoid unfavorable ones. Local adapation can result in variation in seasonal responses, but the genetic basis and evolutionary history of this variation remains elusive. Many insects, including *Drosophila melanogaster*, are able to undergo an arrest of reproductive development (diapause) in response to unfavorable conditions. In *D*. *melanogaster*, the ability to diapause is more common in high latitude populations, where flies endure harsher winters, and in the spring, reflecting differential survivorship of overwintering populations. Using a novel hybrid swarm-based genome wide association study, we examined the genetic basis and evolutionary history of ovarian diapause. We exposed outbred females to different temperatures and day lengths, characterized ovarian development for over 2800 flies, and reconstructed their complete, phased genomes. We found that diapause, scored at two different developmental cutoffs, has modest heritability, and we identified hundreds of SNPs associated with each of the two phenotypes. Alleles associated with one of the diapause phenotypes tend to be more common at higher latitudes, but these alleles do not show predictable seasonal variation. The collective signal of many small-effect, clinally varying SNPs can plausibly explain latitudinal variation in diapause seen in North America. Alleles associated with diapause are segregating in Zambia, suggesting that variation in diapause relies on ancestral polymorphisms, and both pro- and anti-diapause alleles have experienced selection in North America. Finally, we utilized outdoor mesocosms to track diapause under natural conditions. We found that hybrid swarms reared outdoors evolved increased propensity for diapause in late fall, whereas indoor control populations experienced no such change. Our results indicate that diapause is a complex, quantitative trait with different evolutionary patterns across time and space.

## Author summary

Animals exhibit diverse strategies to cope with unfavorable conditions in temperate, seasonally varying environments. The model fly, *Drosophila melanogaster*, can enter a physiological state known as diapause under winter-like conditions. Diapause is characterized by an absence of egg maturation in females and is thought to conserve energy for survival during stressful times. The ability to diapause is more common in flies from higher latitudes and in offspring from flies that have recently overwintered. Therefore, diapause has been thought to be a recent adaptation to temperate climates. We identified hundreds of genetic variants that affect diapause and found that some vary predictably across latitudes in North America. We found little signal of repeated seasonality in diapause-associated genetic variants, but our populations evolved an increased ability to diapause in the winter when they were exposed to natural conditions. Combined, our results suggest that diapause-associated variants evolve differently across space and time. We find little evidence that diapause evolved recently in temperate environments; rather, alleles associated with diapause are as common as expected in Zambia, suggesting that diapause may promote survival under stresses other than cold. Our results provide future targets for research into the genetic underpinnings of this complex, ecologically relevant trait.

## Introduction

Organisms exhibit diverse strategies to survive environments that vary in space and time. Populations can undergo local adaptation, producing genotypic combinations that optimize fitness under present environmental conditions but may be less fit in other environments [1]. In temperate, seasonal locales, environmental signals are often used to anticipate the onset of the unfavorable season and induce plastic responses [2–4]. Insects exhibit a spectacular array of plastic responses to changes in season that can affect morphology [5–7], behavior [8], and developmental progression [9–11]. The ability to reprogram or arrest development, potentially for a fixed period of time (*diapause*), allows insects to weather unfavorable conditions in a hardy state of low metabolism [9–13]. Entry into diapause can result in tradeoffs between immediate survival and future growth, longevity, and reproductive success [14–16]. Due to these tradeoffs, some species exhibit local adaptation of their diapause response, producing dramatic differences in life history patterns across space and time [17–22]. Therefore, diapause is a plastic response to environmental changes, but the genetic ability to diapause can also differ across environments as a result of local adaptation. Intraspecific differences in diapause strategies offer an opportunity to study the spatiotemporal variation and evolutionary history of alleles contributing to a critical life history trait.

A number of reproductive life history traits vary in the genetic model organism *Drosophila melanogaster* [18,23]. Female *D*. *melanogaster* are capable of arresting reproductive development and oogenesis when exposed to short day lengths (10 hours light: 14 hours dark, or 10L:14D) and low temperatures (10–14°C) soon after eclosion [24]. These winter-like conditions induce a change in hormonal signaling that prevents reproductive development [25,26]. Physiologically, dormancy is regulated by hormones including insulin [27–29], juvenile hormone [25,30,31], and ecdysteroids [30,32], as well as dopamine and serotonin [33]. *D*. *melanogaster*’s ovarian dormancy is accompanied by metabolic changes [34,35] and is enhanced by starvation conditions [36]. Dormancy is coincident with transcriptional changes in up to half of all genes in *D*. *melanogaster* [37–39] and in other drosophilid species [40–42]. Male *D*. *melanogaster* also arrest spermatogenesis and undergo physiological changes under unfavorable conditions [43]. Dormancy subsequently diverts limited energy to survival rather than to reproduction at the onset of winter, perhaps allowing flies to overwinter *in situ* [44,45] or in local refugia [46]. Whether this dormancy state is a true diapause or a state of quiescence is still open to debate [11], however it is clearly a substantial physiological reprogramming of the normal female egg production program that phenocopies diapause strategies of temperate endemic drosophilids [47–52]. Hereafter, we refer to *D*. *melanogaster’*s ovarian dormancy as diapause, keeping with established terminology in the field [for example, 18,24,26,46, 52].

Clinal variation in the severity of winter is correlated with clinal variation in the ability to enter diapause in *D*. *melanogaster*. Flies from northern locations in North America, such as Maine, are more likely to be capable of entering diapause than those from more southern locations, such as Florida [18]. Additionally, the ability to enter diapause varies seasonally: the offspring of flies captured in the early spring (the survivors of winter, or their direct descendants) have a greater propensity for diapause than the offspring of flies captured in late summer [19], the descendants of lineages that prospered during favorable conditions [53]. Whether the same genetic loci underlie similar spatial and temporal evolution of phenotypes such as diapause remains unknown at a genome-wide level (but see [54]).

Adaptive evolutionary change in diapause propensity [19] during the growing season (~15 generations; see [55]) suggests the existence of tradeoffs related to diapause: while diapause is advantageous in unfavorable conditions, fitness costs occur when diapause is unnecessary [12]. In the absence of diapause-inducing conditions, strains capable of diapause have lower early reproductive success, putting them at a disadvantage when conditions are ideal for rapid reproduction. However, strains able to diapause tend to live longer, have greater reproductive success later in life, and are more tolerant of cold and starvation [15]. Laboratory selection experiments offer further evidence for tradeoffs: outbred flies reared under alternating cold and starvation stress in the lab evolve an increased genetic propensity to diapause, whereas flies reared under benign lab conditions evolve a decreased propensity for diapause [19], as do flies experimentally selected for heat tolerance [56]. Taken together, these findings suggest diapause is an ecologically relevant trait with tradeoffs that underlie local adaptation across both space and time.

In addition to being genetically polymorphic, *D*. *melanogaster*’s diapause is shallow relative to the diapause of other insects, including other drosophilids [57,58]. Females may spontaneously resume ovarian development after ~6 weeks even when diapause-inducing conditions persist [24] (but see [59]), and diapause is rapidly broken if temperature or day length is increased [24]. In a short-lived species with many generations per year, this weak and facultative diapause strategy may be advantageous to allow individuals to quickly reenter reproductive mode soon after conditions become favorable. On the other hand, the relatively recent colonization of temperate habitats [53–57] led to the suggestion that seasonal diapause may be a recently evolved trait [24,60], so the weak diapause of *D*. *melanogaster* might reflect its incipient evolution. Phenotypic plasticity, such as diapause or behavioral modification, is predicted to evolve when the environment varies more rapidly than generation time, whereas fixed alternative strategies are favored when environmental variation occurs more slowly than generation time [61]. However, in temperate *D*. *melanogaster*, the scale of temporal environmental variation is similar to generation time (weeks), which may explain the reversibility of diapause and the substantial genetic variation in diapause induction over seasonal timescales.

Despite our extensive knowledge of the natural history and physiological basis of diapause in *D*. *melanogaster* and other insects, we still know little about the identity and evolutionary history of polymorphisms underlying variation in this critical life history trait [62]. Herein, we sought to identify genetic variants underlying diapause via a genome wide association study (GWAS) and to link these variants to patterns of global polymorphism in *D*. *melanogaster*. After identifying hundreds of single nucleotide polymorphisms (SNPs) with small effects on diapause, we specifically addressed two questions about the evolutionary history of these alleles. First, do SNPs underlying diapause show predictable patterns of genetic variation across latitudes and seasons, with pro-diapause alleles more common in northern latitudes and in the spring? Second, are alleles associated with diapause present in ancestral populations, and have they experienced recent selective sweeps in North America? Our results suggest that the evolution of diapause across spatial gradients may be distinct from its evolution across seasons: while diapause-associated alleles are weakly clinal, they do not tend to vary predictably over seasons across multiple populations. Furthermore, alleles controlling diapause represent ancestral genetic variation, suggesting they may play roles in seasonal, or even general, aspects of stress response in ancestral localities. The favorability of diapause-associated alleles under a variety of stressful conditions could explain the lack of repeated seasonality of these alleles as well as their presence in tropical climates with pronounced seasonality. Our results provide a roadmap to understand the role of small-effect alleles underlying the evolution of a complex, ecologically relevant trait that varies across space and time.

## Results

**Fig 1:**
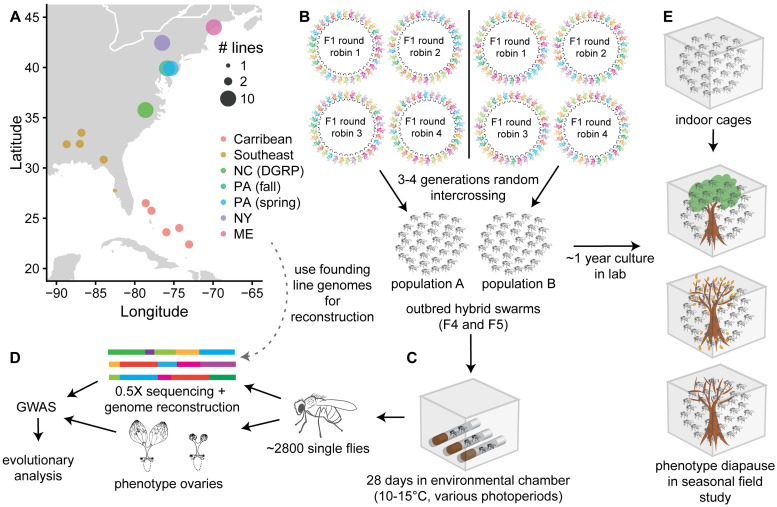
Experimental design for hybrid-swarm based association mapping of diapause. (A) A total of 68 sequenced, inbred lines from seven North American collections were used to initiate hybrid swarm crosses. The lines were divided into two groups of 34 (populations A and B) so that each hybrid swarm had equal representation from the seven collections. Map generated using [64]. (B) Within each population, randomly ordered round-robin crosses were established. The F1 adults were released into cages and propagated with non-overlapping generations. (C) Virgin females from the F4 and F5 generations were collected and placed in environmental chambers with varying photoperiods and temperatures for 28 days. (D) Individual flies were dissected to phenotype diapause. DNA was extracted from the carcasses and individually sequenced to approximately 0.5X coverage. Sequencing reads were used to reconstruct full genome sequences and perform a genome-wide association study; results of the GWAS were integrated with *D*. *melanogaster* population genetic data. E) After one year of laboratory culture, hybrid swarm flies were placed in outdoor cages for a seasonal study of diapause phenotypes; the wild-reared flies were compared to indoor controls. Ovary drawing in (D) modified from [65] under a CC-BY license.

In order to characterize the genetic polymorphisms that underlie variation in diapause in *D*. *melanogaster*, we used a novel hybrid-swarm based mapping approach [63] employing sequenced, genetically diverse inbred lines collected around North America and the Caribbean (Fig 1A; S1 Fig; S1 Table). We intercrossed these lines to produce two outbred populations with recombinant genotypes (populations A and B, Fig 1B; S2 Fig) and then exposed these hybrid individuals to various diapause-inducing conditions in custom-built chambers (Fig 1C). We mapped the genetic basis of diapause by dissecting and genotyping over 2,800 females (Fig 1D). We used the results of this GWAS to analyze patterns of spatial and temporal variation in SNPs associated with diapause and concurrently conducted a field study of diapause incidence of hybrid swarm populations placed in natural conditions (Fig 1E).

### Temperature and nutrition affect diapause

**Fig 2:**
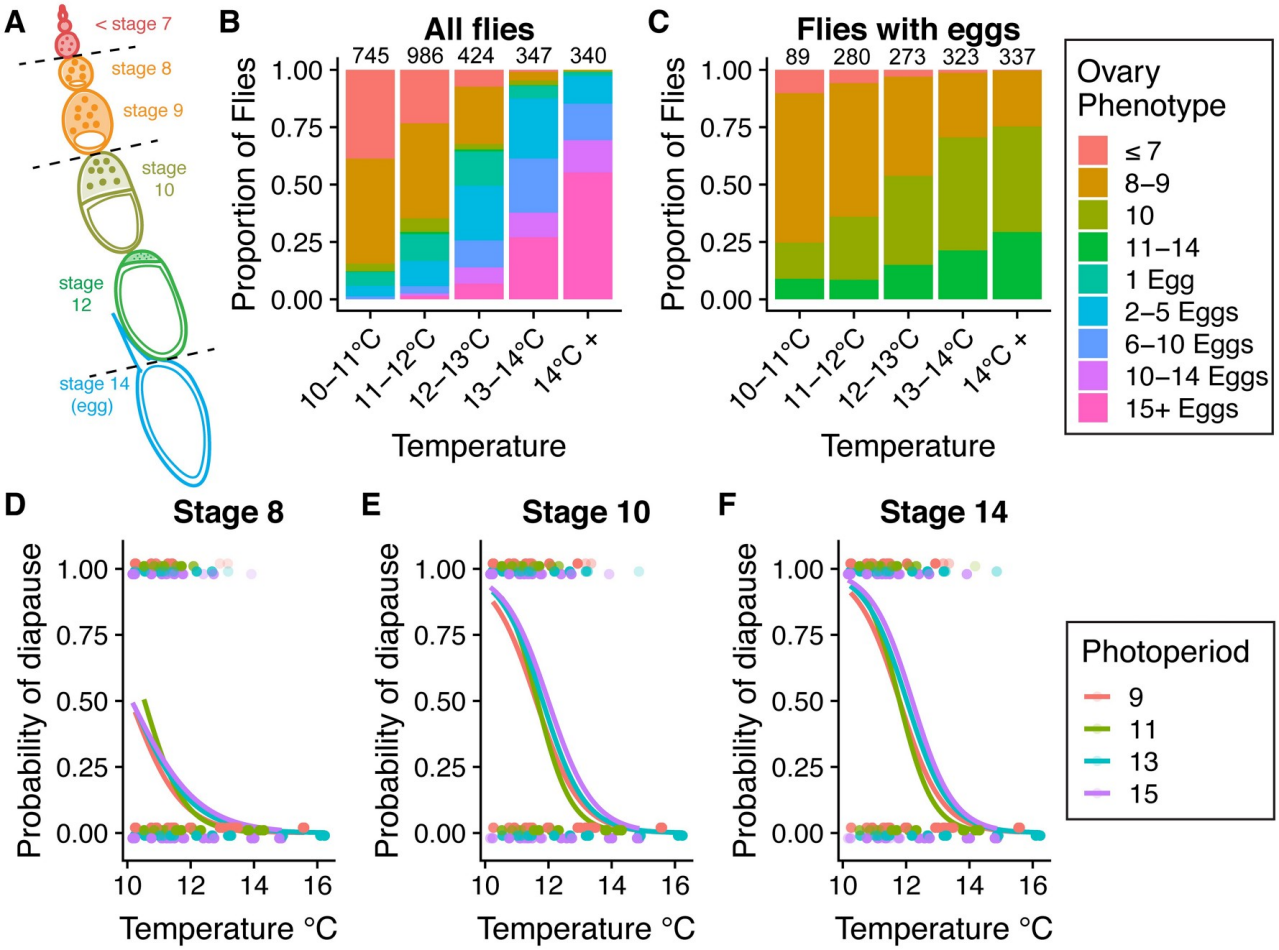
Effects of temperature and photoperiod on diapause in hybrid swarm populations. (A) Ovaries were scored based on the most advanced ovariole stage and the total number of eggs, according to King (1970) [66]. Dashed lines indicate three cutoffs for scoring diapause: stage 8, stage 10, and stage 14. Colors correspond to panels B and C. (B) The most advanced ovariole stage, proportion of flies with eggs, and the total number of eggs per individual all increase with increasing temperature. Numbers above bars indicate the total number of flies phenotyped in each temperature range. (C) Among flies with eggs, the stage of the most advanced egg chamber also increases with temperature. (D-F) Diapause incidence, scored at the stage 8, stage 10, or stage 14 cutoffs, decreases with increasing temperature (binomial general linear model, *P* < 2 x 10^−16^ for all phenotypes). Unexpectedly, longer photoperiods (shown as hours of light: hours of dark) result in increased diapause incidence (binomial *glm*, *P* = 0.002, *P* = 2.7 x 10^−7^, *P* = 5.0 x 10^−8^, respectively). Points represent individual fly phenotypes (diapause = 1, non-diapause = 0).

We initially assessed ovarian development in F4 and F5 hybrid swarm individuals exposed to a range of cool temperatures (10–16 °C, S3 Fig) and photoperiods representing the approximate range of day lengths experienced by our northernmost Maine population. We recorded the stage of the most advanced non-stage 14 ovariole [66] (Fig 2A) and counted the number of mature eggs in each individual. We found a strong effect of temperature on ovarian development: higher temperatures led to more advanced ovarian development and more eggs (Fig 2A and 2B). When examining the most advanced pre-stage 14 ovariole in flies that had produced eggs, we also found that higher temperatures increased the proportion of individuals with advanced ovariole stages (Fig 2C).

We initially scored diapause as an absence of development past three different ovariole stages [66]: stage 8 (no ovarioles past stage 7), stage 10 (no ovarioles past stage 9), and stage 14 (no mature eggs present) (Fig 2A). Diapause at stage 8 and stage 10 were modestly correlated (Pearson correlation, *R* = 0.49, *P* = 2 x 10^−172^) and diapause at stage 10 and stage 14 were strongly correlated (*R* = 0.92, *P* < 1 x 10^−200^). After accounting for differences caused by temperature, photoperiod had an effect on diapause in the opposite direction predicted: individuals exposed to long day (13L:11D or 15L:9D) light cycles tended to have higher diapause induction (Fig 2D–2F), regardless of the ovariole stage cutoff used to determine diapause. Based on recent studies [35], previous work [18,19,52,59,67], and developmental evidence that a checkpoint exists between stages 9 and 10 in ovariole development [68–70], we chose to classify diapause either as an absence of ovariole development to stage 8 (Fig 2D) or stage 10 (Fig 2E) for the remaining genetic analysis.

We next investigated other factors besides temperature and photoperiod that may affect diapause. The F4 generation of our outbred populations had increased diapause incidence at all temperatures (S4A Fig; *P* < 2 x 10^−16^) relative to generation F5, potentially reflecting inadvertent rearing differences (such as larval density) between generations. Outbred populations A and B also showed significantly different diapause induction across temperatures (S4B Fig, *P* < 0.01), though the differences were much less pronounced than the effect of generation. In an experiment conducted in the F20 generation of the hybrid swarm, we found that feeding adults supplemental live yeast substantially reduced diapause incidence across a range of temperatures, further suggesting that adult nutrition and temperature contribute to diapause induction (S5 Fig, *P* < 1 x 10^−10^).

Lastly, we tested for an influence of *Wolbachia* infection on diapause. We quantified the proportion of sequencing reads mapping to the *Wolbachia* genome for each hybrid individual to infer *Wolbachia* infection status. We found that *Wolbachia* decreased the likelihood of diapause at stage 8 (diapause was observed in 17% of *Wolbachia*-infected individuals vs 23% of uninfected individuals; Fisher’s exact test; *P* = 0.0001) but did not influence diapause at stage 10 (*P* = 0.31). Placing all of these variables into a single model revealed that temperature and generation accounted for the majority of explainable variation in the diapause phenotype, though more than 50% of the variation remains unexplained by environmental factors (S2 Table).

### Diapause is heritable with a polygenic signal

**Fig 3:**
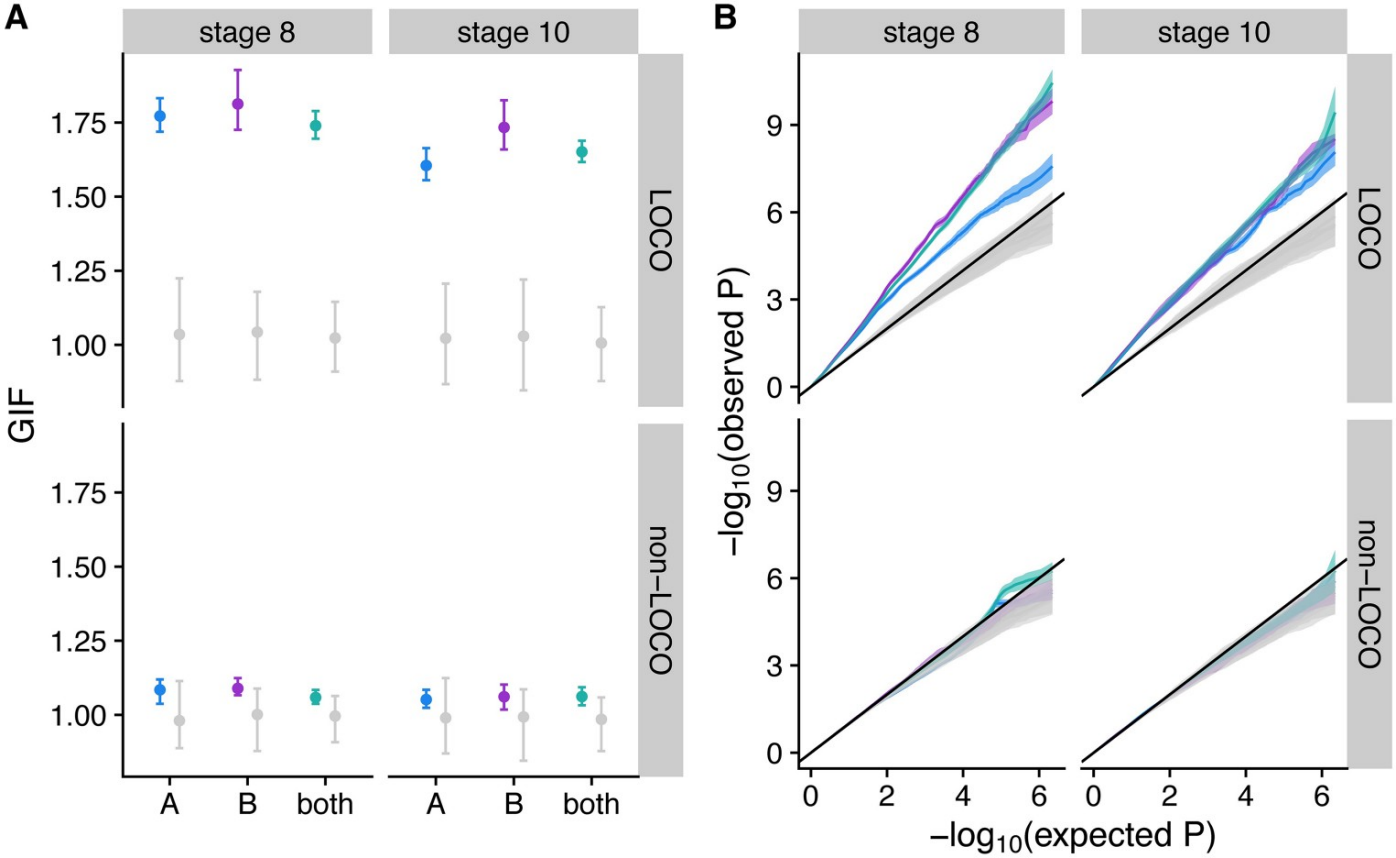
A leave-one-chromosome-out (LOCO) genetic relatedness matrix results in inflated *P*-values in actual but not permuted GWAS. (A) Genomic inflation factor (GIF or λ_GC_) for 100 imputations of actual data and 100–1000 permutations for LOCO (top) and non-LOCO (bottom) GWAS of two diapause phenotypes. Points represent the median and bars extend to the 2.5 an 97.5% quantiles. Grey bars show permutations for each mapping population. (B) Average quantile-quantile plots (blue = A, purple = B, teal = both, grey = permutations) for each GWAS. Observed *P*-values were averaged for each expected *P* -value across all imputation/permuations. Ribbons represent standard deviation; black lines have slope of 1.

To characterize the genetic architecture of natural variation in diapause, we performed a GWAS with reconstructed genotypes (S6 Fig) from 2,740 hybrid individuals for diapause scored at two cutoffs. We performed this analysis using 100 different imputations of missing genotype data (see Methods) in populations A and B separately, as well as the two populations combined (“both”). We also generated 1000 permutations of the combined dataset and 100 permutations of each individual population. Importantly, these permutations preserved the linkage structure among SNPs and the relationship between environment and phenotype, while randomizing the relationship between genotype and phenotype. We initially conducted the GWAS incorporating genetic related matrices (GRMs) generated with a leave-one-chromosome-out (LOCO) approach to avoid proximal contamination: the deflation of GWAS effects caused by using a GRM that includes the focal SNP and linked SNPs [71,72]. We then calculated the genomic inflation factor, λ_GC_, for the mapping results of each phenotype in each permutation. We discovered that in our study design, a LOCO approach resulted an inflation of the genomic inflation factor (λ_GC_ > 1.5, Fig 3A) and an excess of low *P*-values observed in a quantile-quantile plot (Fig 3B). This inflation of genetic signal was not due to population structure, as it was observed in populations A and B separately as well as the combined populations. Further, inflation was seen only in the actual data, and not in the permutations, suggesting that true, linked genetic signal in the actual data was exaggerated by excluding nearby SNPs from the GRM. In the permutations, where no true genetic signal exists, we observed no such inflation. As an alternative approach, we included all SNPs in the GRM (non-LOCO analysis), which leads to an overcorrection that reduces GWAS signal, because the potential phenotypic effect of the SNP has been accounted for in the GRM. This non-LOCO analysis resulted in λ_GC_ values and quantile-quantile plots that are similar between permuted and observed data (Fig 3A and 3B), across all mapping populations and chromosome arms (S7 Fig).

Log-transformed LOCO and non-LOCO *P*-values were highly correlated (Spearman rank correlation, *R* ranges between 0.83 and 1.0). The correlation of *P*-values was substantially higher in permuted relative to non-permuted data (mean ± standard deviation: *R*_permuted_ = 0.99 ± 0.006; *R*_observed_ = 0.89 ± 0.016). This observation further suggests that true genetic information incorporated into the GRM influences the mapping when the data are in their original order, but the GRM is less influential for the mapping when the data are permuted. However, because all LOCO and non-LOCO *P*-values are highly correlated, we decided to proceed in our analysis with the more conservative non-LOCO GWAS, which likely underestimates the effect of individual SNPs due to proximal contamination.

**Fig 4:**
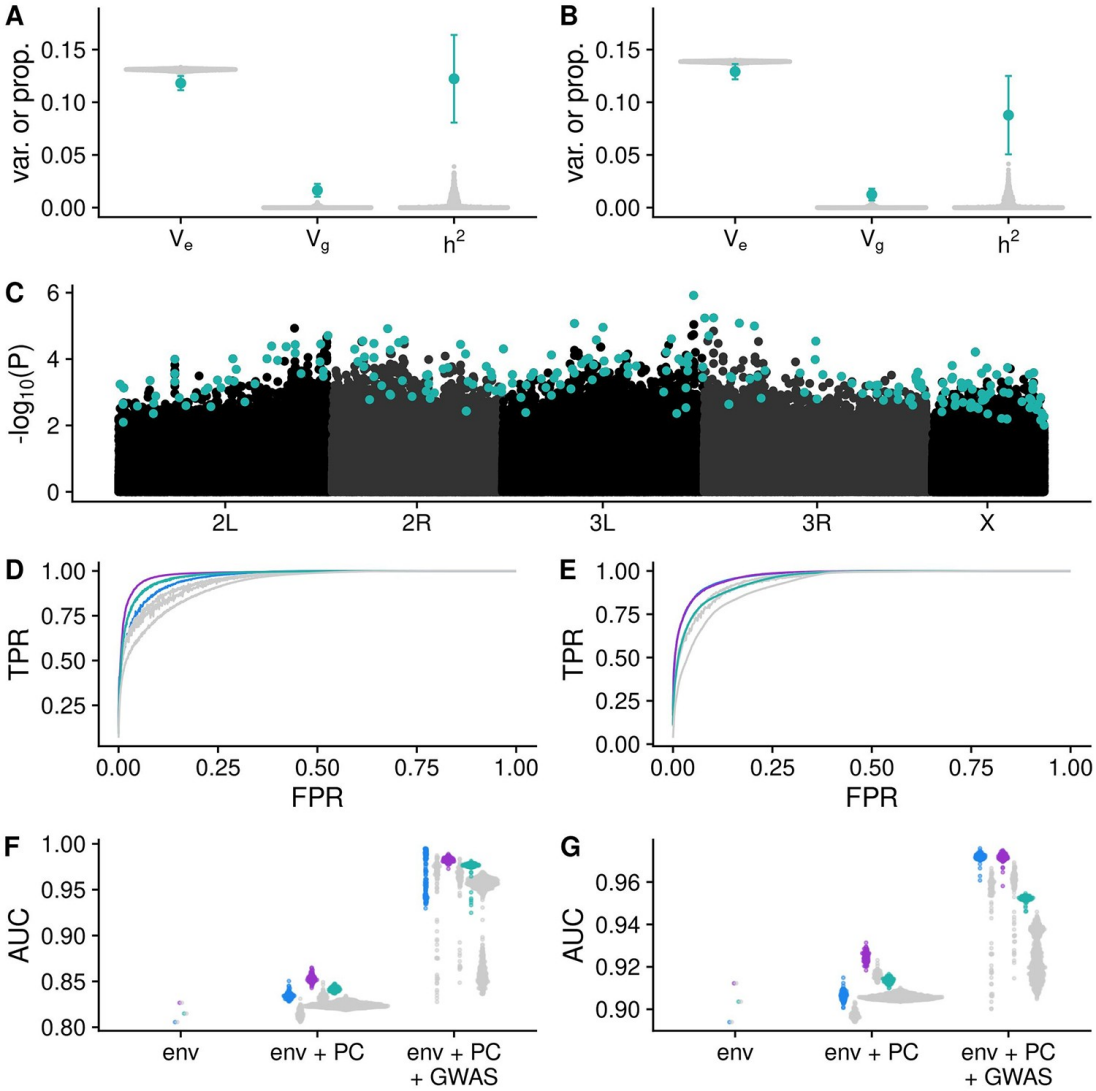
Diapause is a polygenic trait. (A-B) V_g_, V_e_ and heritabiilty estimates for stage 8 (A) and stage 10 (B) diapause phenotypes. Teal points indicate observed estimates +/- 95% confidence interval for heritability in the hybrid swarm (populations A and B combined). Grey points indicate heritability estimates for 1000 permutations. (C) Manhattan plot for GENESIS *P*-values for stage 10 diapause in one imputation. Teal points indicate LASSO SNPs. (D-E) Average receiver operating characteristic (ROC) curves for stage 8 (D) and stage 10 (E) diapause predictions made using LASSO SNPs (blue = A, purple = B, teal = both, grey = permutations). Phenotypes for each individual in the mapping population were predicted using the informative environmental variables, genetic principal components, and SNPs chosen by LASSO. At any given false positive rate (FPR), the true positive rate was averaged across all imputations or permutations. Observed data (colored lines) have higher true positive rates (TPR) relative to permutated GWAS (gray lines). (F-G) Quantification of ROC analysis using area under the curve metric (AUC) for stage 8 (F) and stage 10 (G) phenotypes. “env” is a model containing only environmental data; “env + PC” includes environmental data and 32 principal components; “env + PC + GWAS” includes the former plus the genotypes of up to several hundred SNPs chosen by LASSO.

The two GWAS strategies suggested that true genetic signal in the hybrid swarm influences diapause; to confirm this finding, we estimated narrow-sense heritability of diapause using genome-wide genotypes in a restricted maximum likelihood analysis in GCTA [73,74]. We estimated heritabilities of approximately 0.12 and 0.08 for stage 8 and 10 diapause, respectively, using all genotype data from both populations combined. These estimates far exceeded the heritability calculated for 1,000 permuted phenotype datasets (Fig 4A and 4B).

Visual inspection of a representative Manhattan plot also revealed a broadly polygenic signal of diapause, with SNPs scattered throughout the genome (Fig 4C).

Following the GWAS, we used LASSO [75] to identify a subset of unlinked, informative SNPs (hereafter, “LASSO SNPs”) from the top 10,000 SNPs ranked by *P*-value (Fig 4C). LASSO takes a number of potential predictor variables and chooses those that are most informative yet independent [76]. Each LASSO model started with environmental covariates, the top 32 principal components derived from genome-wide SNPs, and the SNP genotypes; the resulting model usually retained several hundred SNPs. LASSO SNPs (S6 Table) showed low levels of LD (S8 Fig), suggesting the algorithm was successful in choosing unlinked informative markers. In general, a larger number of LASSO SNPs were identified in the observed data relative to permutations, but this excess was only significant in the combined mapping population (S9 Fig), demonstrating increased genetic signal in the true ordering of the data. LASSO SNPs are variable between imputations; of a total of 38,442 LASSO SNPs identified in various iterations of the GWAS, nearly half (18,632) were found in only one imputation, and only 333 were found in 50 or more imputations. Therefore, we note that LASSO SNPs may be markers of important haplotypes and not causative loci.

To determine whether the SNPs identified by LASSO were in fact informative for predicting diapause phenotypes, we implemented a receiver operating characteristic curve (ROC) analysis [77]. The SNPs chosen by LASSO substantially improved the accuracy of the predicted phenotypes, with a higher true positive rate (TPR) and lower false positive rate (FPR) in the observed data relative to the permutations (Fig 4D and 4E, compare blue/green/purple lines to grey). This difference can be quantified using the area under the curve (AUC), which was higher for predictions in the observed data relative to the permutations for both phenotypes (Fig 4F and 4G). Additionally, we found that for both phenotypes, adding genetic principal components to the model improved the model relative to environment alone, and adding LASSO SNPs further improved the model over principal components plus environment (Fig 4F and 4G). This improvement was greater in the actual data relative to the permutations; combined, these observations suggest true genetic signal in the observed data.

We tested for shared genetic signal for diapause between the two mapping populations by counting the number of SNPs shared between populations A and B for each imputation and permutation of the data. We found that the number of shared LASSO SNPs or quantile-ranked SNPs was very small, and the overlap between SNPs identified from actual data did not exceed the overlap between SNPs identified in permutations (S10A Fig), suggesting that the genetic architecture of diapause may be dependent on the mapping population. However, we did observe an excess of shared SNPs associated with both of the two phenotypes. We found that for the quantile-ranked cutoffs, the number of SNPs shared in the observed data was generally greater than the number of SNPs shared between permutations (S10B Fig). This finding suggests that some loci affect diapause at both stages. But, we note that like LASSO SNPs, top quantile-ranked SNPs are not entirely consistent across imputations. For example, when considering stage 8 diapause in both populations, a total of 6,145 SNPs fall into the top 0.1% of SNPs in at least one imputation. Of these, 194 are found in ≥ 90 of imputations, and 2,146 are found in only one imputation (see S6 Table for complete details). In contrast, we observed little overlap of LASSO SNPs between the two phenotypes, again indicating that LASSO SNPs may be markers and not causative.

Intriguingly, we found no effect of two previously described variants (*cpo* SNP: *dm3* chr3R: 13,793,588; and *timeless* indel: chr2L:, 3,504,474) [52,54,60,78] on diapause (S11 Fig). We tested for association between diapause and karyotype at five cosmopolitan inversions [In(2L)Ns, In(2R)t, In(3R)Payne, In(3R)C, and In(3R)Mo] segregating in the hybrid swarm. We found that individuals heterozygous for In(3R)Payne had a modest decrease in diapause induction at both stages in population A and both populations combined (S3 Table); however, these effects were not significant after correcting for multiple testing of the various inversions. Therefore, the cosmopolitan inversions do not appear to play major roles in diapause induction. We also investigated the functional annotation categories of SNPs associated with diapause. The vast majority of diapause-associated SNPs were found in non-coding regions: upstream/downstream of annotated genes, or in introns (S4 Table). However, there was little signal of enrichment or de-enrichment for particular classes of diapause-associated SNPs relative to permutations (S5 Table).

### Linkage is not responsible for the polygenic basis of diapause

**Fig 5:**
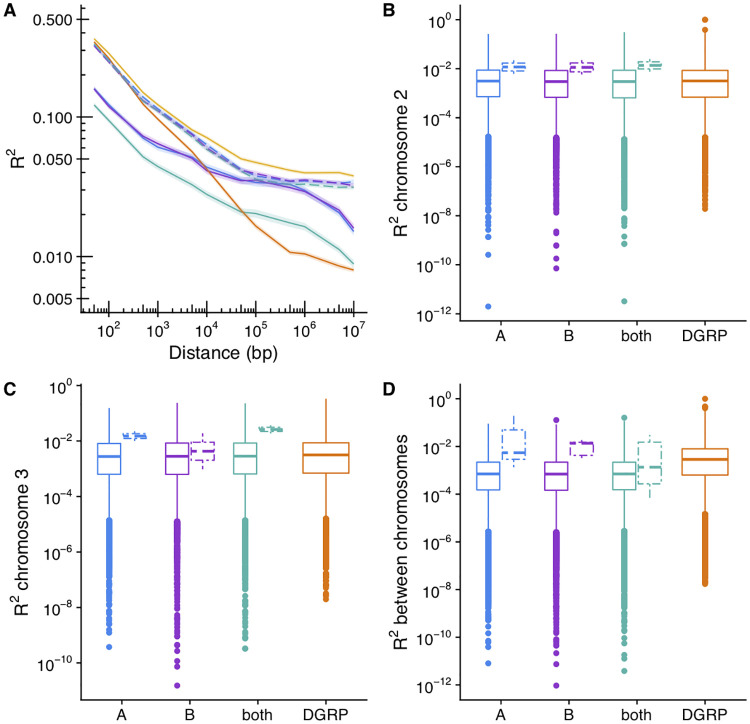
Analysis of linkage disequilibrium supports a polygenic basis of diapause. (A): Linkage disequilibrium (LD) decay for SNPs with minor allele frequency > 0.05 in the hybrid swarm (blue = A, purple = B, teal = both) contrasted to the DGRP (orange) and the DGRP down-sampled to 34 lines (yellow). Dashed lines indicate parents of the hybrid swarms, while solid lines indicate the hybrid offspring. 10,000 SNPs were randomly sampled and LD (R^2^) to nearby SNPs at fixed distances was measured. Lines represent median LD; ribbons represent the 95% confidence intervals. (B-D) Long distance LD between pairs of SNPs randomly sampled from 2L and 2R (B), 3L and 3R (C), or sampled from different chromosomes (D). Dashed box plots indicate parental lines.

Collectively, these results suggest that many loci throughout the genome contribute to phenotypic variation in diapause. Alternatively, the nature of our mapping population might increase linkage disequilibrium (LD), potentially causing large linkage blocks to be associated with diapause. To test the latter possibility, we examined the general signal of LD in our mapping population in contrast with a wild-derived population from North Carolina (the Drosophila Genetic Reference Panel, or DGRP [79]). We also analyzed the two sets of 34 parental lines that produced the hybrid swarms, and randomly down-sampled the DGRP to 34 lines for comparison. Four to five generations of mating reduced LD in the hybrids relative to the parental lines (Fig 5A and 5B; compare solid and dashed lines). The LD of the DGRP is slightly lower than that of the hybrid swarm at large distances (>10^5^ bp; compare orange and teal) but higher at shorter distances. This effect is partially mediated by the number of lines, as the down-sampled DGRP (yellow) had higher LD than the hybrid swarm at all distances. None of the mapping populations demonstrated substantial long-distance LD between arms of the same chromosome or across chromosomes (Fig 5B–5D), and long-distance LD was reduced in the hybrid swarm relative to parental populations (compare solid boxplots to dashed boxplots). Therefore, while LD between nearby SNPs may contribute to some signal in the GWAS, the polygenic signal is not solely due to linkage, since LD drops below 0.05 within ~10 kb in the F4s and F5s of the hybrid swarm.

### Diapause-associated SNPs are likely to be clinal, but not consistently seasonal

We tested for signatures of local adaptation across space (a north to south cline in North America) and time (between fall and spring) by intersecting the GWAS results with existing datasets of spatiotemporal SNP variation in *D*. *melanogaster* [53,80]. Based on *D*. *melanogaster’s* natural history [18,19], we predicted that pro-diapause alleles (which increase diapause in the GWAS) would be at higher frequency in the north and in the spring. We used LASSO SNPs as well as genome-wide quantile thresholds to conduct this analysis. We calculated a polygenic score [81,82] by multiplying the clinal or seasonal effect size (including sign) by the GWAS effect size and sign and summing this product across all SNPs, all LASSO SNPs, the top 0.01% of the GWAS, or the top 0.1% of the GWAS (see S6 Table and Materials and methods for details). In this test, we predict that concordant signal in the GWAS and the clinal/seasonal datasets should result in positive polygenic scores that exceed permutations. We identified significant signal as cases in which over 50% of the imputations scored within the most extreme 5% of the permutations.

**Fig 6:**
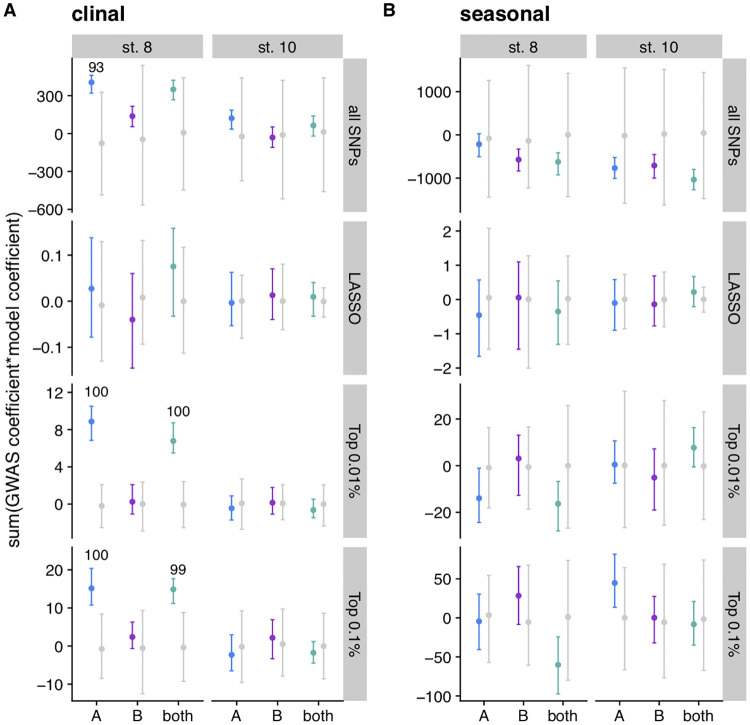
SNPs associated with diapause at stage 8 vary predictably across latitudinal clines. (A) Polygenic scores calculated by multiplying clinal effect size reported in Bergland *et al* (2014) and GWAS effect size for each SNP and summing across all SNPs, LASSO SNPs, and the top 0.01% or 0.1% of the SNPs in the GWAS. Effect sizes are polarized such that positive numbers indicated pro-diapause alleles are more common in the north. (B) Polygenic scores calculated for seasonal data by multiplying seasonal effect size reported in Bergland *et al* (2014) and GWAS effect size, polarized so that pro-diapause alleles and alleles with higher frequency in spring are positive. Data are shown with a point for the mean and error bars extending to the 2.5% and 97.5% quantiles. Colored points indicate actual data for 100 imputations of each mapping population; grey points indicate the distribution for permutations. Numbers indicate the percentage of imputations that exceed the 97.5% quantile of the permutations, if greater than 50%.

We first compared our data to the results from Bergland *et al* (2014), which sampled allele frequencies across a latitudinal cline and also identified SNPs that repeatedly oscillate in allele frequency between spring and fall in a single Pennsylvania orchard over three years. We found that in population A and both populations combined, the average clinal polygenic score of the actual data was positive and exceeded the permutations for the stage 8 diapause phenotype for the top 0.01% and 0.1% of GWAS SNPs (Fig 6A). Therefore, the combination of many small-effect alleles that vary clinally could produce the clinal variation of diapause observed in North America. This trend was not observed in LASSO SNPs, suggesting that LASSO SNPs may not be the subset of SNPs with the greatest ecological relevance, or that the clinal signal that we observe is driven by linked sites among quantile-ranked SNPs.

We also observed a significantly positive clinal trend when looking at all SNPs in population A (93/100 imputations have a clinal polygenic score greater than 97.5% of permutations when summing across all SNPs). This finding could be caused by the effect of the most significant SNPs, or due to a strong clinal signal across a larger percentage of SNPs in the genome that are weakly associated with diapause. The positive clinal signal remained even when excluding top SNPs from the analysis (S17 Fig), suggesting pervasive parallelism of small diapause effects and clinal changes in allele frequencies. No strong clinal trends were observed for the SNPs associated with stage 10 diapause, suggesting the possibility of different spatial selection pressures on SNPs underlying the two phenotypes. Furthermore, no strong seasonal trends were observed for either phenotype (Fig 6B), suggesting that pro-diapause alleles in our mapping population do not repeatedly increase in frequency in spring relative to fall in natural populations.

We performed the same analysis on seasonal allele frequency data collected by Machado *et al* (2019), which also sampled a cline and compared spring and fall allele frequencies in 20 populations sampled from North America and Europe. We see similar trends in clinal signal in diapause-associated alleles in this dataset and no seasonal signal (S12 Fig). The lack of predictable polygenic signal in the seasonal tests led to the intriguing possibility that diapause might evolve seasonally via changes in allele frequencies of different SNPs in different populations. We tested for concordant signal of diapause-associated SNPs among seasonally varying polymorphisms from 20 individual locales sampled in Machado *et al* 2019 [80]. Ten geographic populations showed stronger than expected seasonal allele frequency changes of top 0.01% diapause-associated SNPs for the stage 8 phenotype, but not stage 10, in hybrid populations A and both (S13 and S14 Figs). However, the direction of this effect varied, with some locales showing an excess of pro-diapause alleles in the spring (3 populations with significantly positive scores), and others showing an excess of pro-diapause alleles in the fall (10 populations with significantly negative scores). Therefore, we suggest that seasonal evolution of diapause-associated SNPs can and does occur in individual populations, but in an unpredictable manner across years and populations.

### Hybrid swarms evolve an increased capacity for diapause in the winter in natural environments

**Fig 7:**
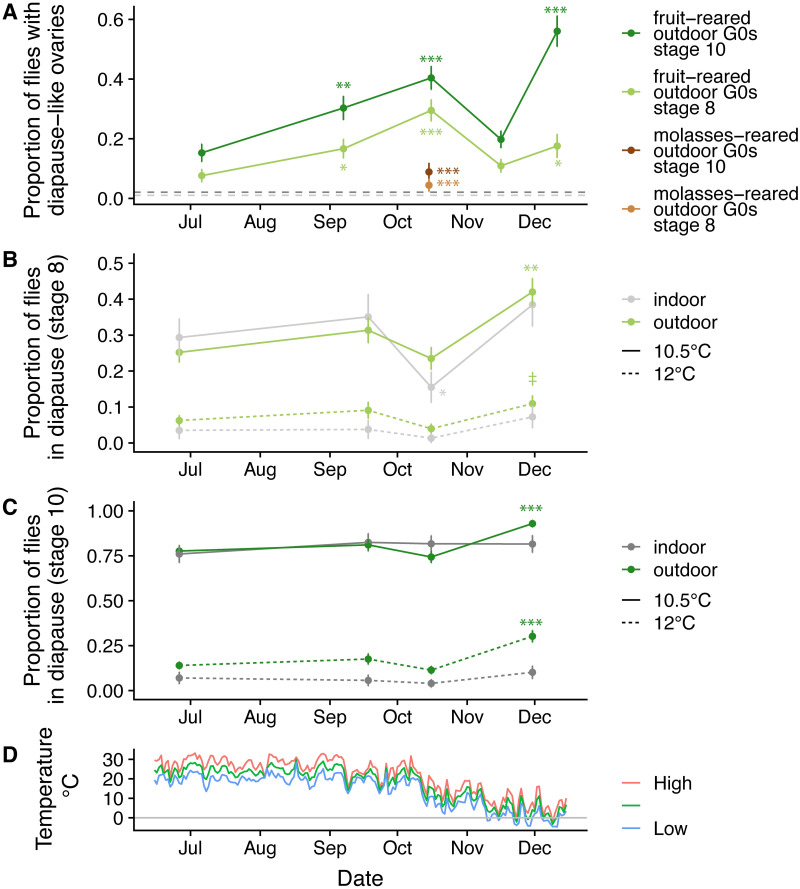
Plasticity and selection contribute to increased diapause in late fall in field-reared samples. (A) Ovaries were dissected from outdoor cage flies reared on fruit and diapause was assessed at stage 8 (light green) or 10 (dark green). A general linear model was used to determine whether diapause at later collection dates differed significantly the first collection on June 26^th^, 2018. (‡ P < 0.1, * *P* < 0.05, ** *P* < 0.01, *** *P* < 1x 10^−4^). Samples were also collected at a single time point from cages reared on cornmeal-molasses food (brown). This sample had significantly lower diapause incidence than fruit-reared samples collected one day later at both stages. The horizontal grey lines indicate diapause incidence for stage 8 (light grey) and stage 10 (dark grey) in a single sample of 96 mated, 5–8 day old flies reared in laboratory cages on cornmeal-molasses food. (B) Field cage and laboratory cage flies were collected at several timepoints and reared in the lab for two generations before assessing diapause at stage 8 in the standard assay at either 10.5 °C (solid lines) or 12 °C (dashed lines). Diapause was marginally increased in the December outdoor sample at 12 °C (*P* = 0.08) and significantly increased in December at 10.5 °C (*P* = 0.0002), whereas it remained relatively consistent across indoor flies. (C) Same as B, but phenotypes for stage 10 diapause. Diapause increased in the December sample of outdoor cage flies relative to the first sample from June (general linear model, *P* = 3.7 x 10^−5^ at 12 °C, *P* = 6.1 x 10^−5^ at 10.5 °C), while diapause was consistent across samples in the lab-reared flies (*P* > 0.05 for all pairwise comparisons). (D) Weather Underground temperature data during the field season for Carter Mountain, VA, approximately 2 km from our field site.

To study the natural seasonal dynamics of diapause expression, we introduced our hybrid swarm populations to outdoor cages fed regularly with rotting apples and bananas to experience semi-natural “wild” conditions (S15 Fig). These cages were not density controlled and were composed of individuals of various ages because they were allowed to propagate freely, with overlapping generations. We sampled flies periodically from these cages over six months and assessed ovary status in the captured females. Surprisingly, we found that a substantial proportion of flies appeared to be in diapause, even during the favorable months of summer and early fall. We refer to these flies as “diapause-like” since they were not placed in the standard laboratory diapause assay as virgin females. All field-caught samples (G0) from fruit-fed cages (Fig 7A, green lines) had higher incidence of diapause-like ovaries than a reference sample of flies reared in lab cages at 25°C (Fig 7A, grey dashed lines). The single highest rate of diapause-like ovaries for stage 10 was observed from a sample in mid-December that was collected in sub-freezing conditions (Fig 7C), suggesting that cold temperatures may be one of several factors that reduce ovary maturation in natural conditions. However, the highest diapause incidence for stage 8 was observed in October, suggesting other factors besides cold temperature may contribute as well, and the two phenotypes may respond to somewhat different signals. Intriguingly, diapause incidence of field caught samples was quite low in November, even though conditions were cool relative to previous months. This observation further suggests that environmental factors beyond temperature mediate ovary development.

Diapause in the field was influenced by nutrition: flies sampled from a separate set of outdoor cages that were fed cornmeal-molasses food revealed a much lower proportion of flies in diapause when compared to fruit-reared flies captured just one day later (Fig 7A; compare brown and green points in October). Therefore, fruit consumption, or the microbiota associated with fruit in the wild [83], may cause reduced ovary maturation relative to standard lab medium. This finding agrees with our result that live yeast diminishes diapause (S3 Fig); the large quantity of deactivated yeast present in the cornmeal-molasses food, or other nutritional differences between the two substrates [84], may have enhanced ovary development in outdoor flies fed standard food. However, we cannot rule out additional differences between indoor and outdoor cages since density and age of sampled flies were not controlled in the outdoor cages. Nonetheless, the fruit-fed cages, which more closely resemble the environment of wild flies in an orchard or compost pile, may more accurately represent the natural state of investment in reproduction in the wild; rich laboratory fly medium may permit more oogenesis than normally occurs in nature [85–88].

To test the hypothesis that evolved genetic changes in the outdoor populations also contributed to increased diapause propensity in early winter, we captured seasonal samples from outdoor cages and reared them in the lab for two generations (“G2”). We exposed the G2 offspring to diapause-inducing conditions to perform a common-garden assay of diapause in flies that evolved under natural conditions. We concurrently sampled flies from our indoor hybrid swarm cages and bred them under identical conditions as negative controls. We found that the outdoor populations evolved a significantly increased propensity for diapause in late fall for both diapause phenotypes, whereas the indoor populations experienced few significant changes in diapause propensity (Fig 7B and 7C). This result was observed in flies held at two different diapause-inducing temperatures (10.5 °C and 12 °C), and the changes in diapause incidence were stronger for stage 10 diapause (Fig 7C) relative to stage 8 (Fig 7B). We note that both indoor and outdoor cages experienced parallel changes in diapause, likely due to experimental variation between cohorts. However, the magnitude of change from the first collection to the final collection was significant for outdoor cages (*P* < 0.05 for three of four tests, Fig 7B and 7C) but not indoor cages (*P* > 0.05 for all tests). The evolved change in diapause corresponds to the onset in mid-November of sustained cold conditions at our field site (Fig 7D), suggesting that these conditions may have caused selection for individuals able to diapause. Collectively, our field results suggest that environmental conditions produce plastic changes in ovary development and also result in selection for increased propensity to diapause. We note that the results of these field experiments may have been influenced by potential invasion of wild flies into our field cages, which would change the genetic composition of the *D*. *melanogaster* populations. Based on the ratio of *D*. *simulans* to *D*. *melanogaster* at nearby Carter Mountain Orchard in Charlottesville, VA, we estimate that at most 5% of *D*. *melanogaster* in the cages were invaders (see Methods). However, even if the seasonal signal was driven by contaminating flies that outcompeted the hybrid swarm, it still suggests an increase in the survival of diapause-prone flies in winter field conditions.

### Diapause-associated SNPs are present in Zambia

**Fig 8:**
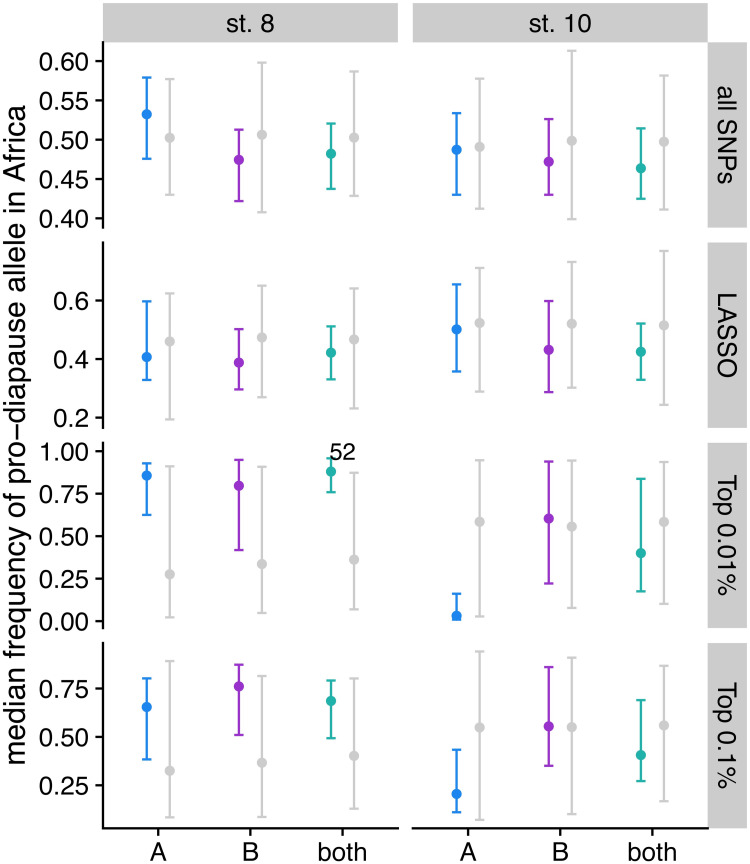
Diapause-associated alleles are as common as expected in Zambian flies. The median allele frequency of pro-diapause alleles for all SNPs in the GWAS, LASSO SNPs, the top 0.01% of the GWAS, and the top 0.1% of the GWAS for stage 8 (left) and stage 10 (right). Points represent median of the imputations/permutations; error bars show 2.5% to 97.5% quantiles. Grey points/bars are 100 permutations for populations A and B; 1000 permutations for both. Colored points/bars are 100 imputations of the original data. Text indicates the percentage of imputations that exceed the 97.5% quantile of the permutations, if that percentage is greater than 50%.

Diapause has been proposed to be a recent adaptation to seasonal cold in temperate populations of *D*. *melanogaster* [12,24,89], but see [35,90]. To test whether the diapause-associated alleles mapped here are present in ancestral populations, we first examined the median allele frequency of pro-diapause alleles in a large sample of flies from Zambia [91,92]. We predicted alleles that increase diapause would have lower allele frequencies in Zambia than the pro-diapause alleles identified in permutations if pro-diapause alleles were recently selected in temperate habitats. We found that the actual median allele frequency of pro-diapause alleles falls within the expected range of allele frequencies based on permutations for both phenotypes (Fig 8). The occurrence of these alleles in Zambia is not due to an excess of diapause associated SNPs in regions of admixture with European flies (S16 Fig). The presence of pro-diapause alleles at expected frequencies suggests they are longstanding polymorphisms that are not deleterious in ancestral climates.

### Evidence for partial sweeps of anti-diapause alleles in North America

**Fig 9:**
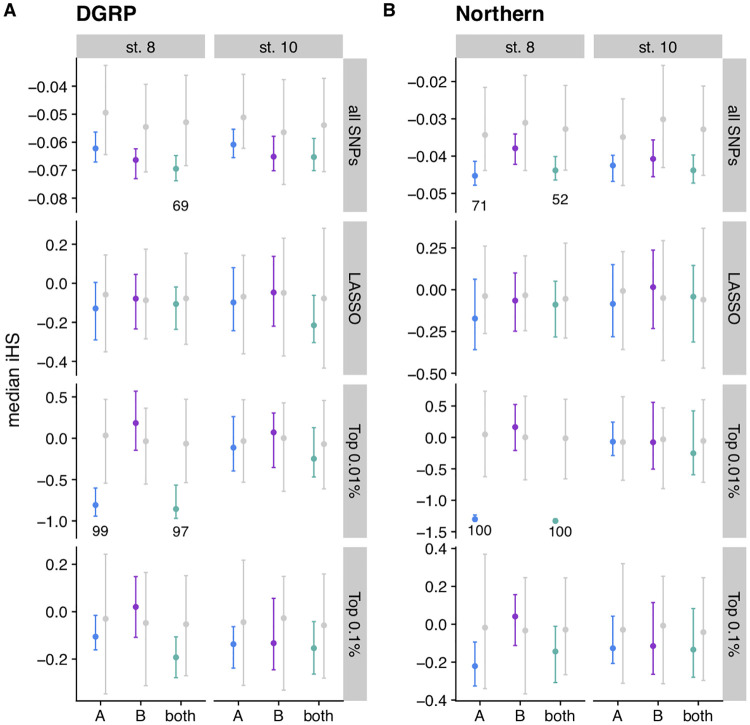
Integrated haplotype homozygosity score (iHS) of diapause-associated SNPs in two populations. (A) iHS was calculated for every SNP in the DGRP, with ancestral and derived states polarized based on direction of diapause effect. For each GWAS, the median iHS was calculated for all SNPs, the LASSO SNPs, the top 0.01% of SNPs in the GWAS, and the top 0.1% of SNPs in the GWAS for each phenotype. Points represent the median; error bars extend to the 2.5% to 97.5% quantiles. Colored bars are 100 imputations of the original data. Grey bars are 100 permutations for populations A and B; 1000 permutations for both. (B) Same as panel A, but using a set of 205 lines collected from Pennsylvania and Maine (“Northern”). Text indicates the percentage of imputations that are lower than the 2.5% quantile of the permutations, if that percentage is greater than 50%.

We tested for signals of partial selective sweeps on pro-diapause alleles in North America. We used the DGRP, from Raleigh, North Carolina [79], and a set of sequenced, inbred lines collected from Pennsylvania and Maine (“Northern” lines) to calculate the integrated haplotype homozygosity score (iHS) [93] for the pro-diapause alleles identified in the GWAS. In this test, an elevated iHS would suggest that the pro-diapause allele is found on a longer shared haplotype, suggesting a recent sweep, whereas depressed iHS would suggest a sweep of the anti-diapause allele. Median iHS of stage 8 top 0.01% diapause SNPs was depressed relative to permutations in both the DGRP and Northern populations (Fig 9), suggesting the possibility of partial selective sweeps that increased the frequency of anti-diapause alleles. This trend was also seen, albeit more weakly, when looking at IHS of *all* SNPs (polarized based on the direction of diapause effect), suggesting potential partial sweeps on a large number of SNPs underlying this polygenic trait. This signal generally persisted when excluding top-ranked SNPs from the analysis (S17 Fig), suggesting that on a broad scale, alleles associated with decreases in diapause propensity are found on longer haplotypes in North American populations.

### Linked SNPs on the X chromosome drive population genetic signals in the top 0.01% of diapause-associated SNPs

**Fig 10:**
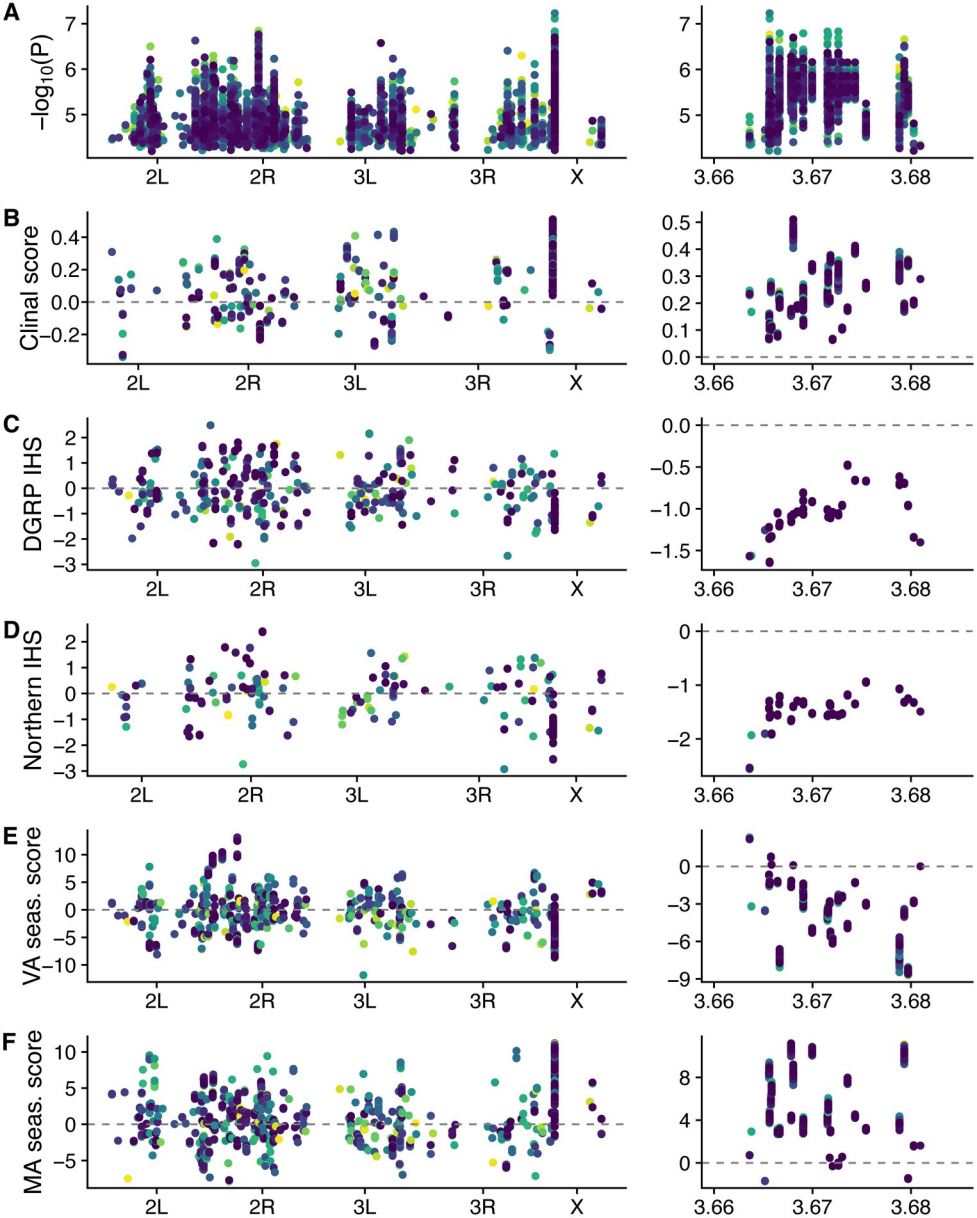
A small region near *tlk* on the X chromosome drives population genetic signal in the top 0.01% of SNPs. The top 0.01% of SNPs for the combined mapping population for stage 8 diapause are color coded by their imputation number. Left panels show the full genome; right panel is focused on a region within the gene *tlk* (ChrX:3,660,000–3,685,000). (A) Manhattan plot. (B) Clinal polygenic scores using data from Bergland *et al* 2014. (C-D) IHS in the DGRP and Northern populations. (E) Seasonal polygenic scores from Charlottesville, VA in 2014. (F) Seasonal polygenic score from Lancaster, MA in 2014. Note that y-axis scales vary between left and right panels.

In several analyses (Figs 6 and 9, S13 Fig), we report strong population genetic signal in the top 0.01% of SNPs underlying diapause at stage 8 in population A and/or the combined populations. To investigate whether this signal was driven by linked SNPs in a small region of the genome or more broadly distributed SNPs, we examined polygenic scores and IHS for individual SNPs in the top 0.01% of diapause-associated SNPs (Fig 10). These plots reveal that closely linked SNPs on the X chromosome (in the region of chrX:3,660,000–3,685,000, dm3) were likely responsible for causing polygenic scores and IHS values that were outliers relative to permutations. While the magnitude of the polygenic scores and IHS in this region of the genome is on par with other regions of the genome, there are multiple SNPs in a small region with concordant signs that contribute to more extreme averages relative to the permutations. These SNPs (S7 Table) are non-coding, intronic, and synonymous variants within and near the genes *tousled-like kinase* and *mir-4962* and suggest that this region of the genome may be of interest for future studies on local adaptation in *Drosophila*. When we exclude all SNPs in a 0.1 Mb region surrounding *tlk* from the analysis, the top 0.01% of SNPs no longer show clinal signal or significantly negative IHS (S18 Fig). However, the significantly concordant clinal signal and negative IHS persist genome-wide across all SNPs when this small region is excluded (S18 Fig). Combined, these results suggest that combination of strong localized signals (*tlk*) and a large number of small-effect SNPs with concordant clinal signal and short haplotypes contribute to the population genetic signal of diapause-associated SNPs.

## Discussion

### The genetic basis of an ecologically relevant trait identified from mapping in a hybrid swarm

Herein, we estimated the genetic architecture of natural variation in ovarian diapause in *D*. *melanogaster* using a novel hybrid-swarm based mapping strategy. Our work changes the interpretation of the genetic basis of variation in diapause in *D*. *melanogaster*, which has previously been characterized as being under the control of a small number of loci with large effect [27,52,60,78]. We studied spatiotemporal variation of small-effect, diapause-associated alleles in global *D*. *melanogaster* populations, finding evidence that both pro- and anti-diapause alleles have experienced selection in different geographic regions and seasons.

The SNPs we identify, which are generally unremarkable in a genomic context (Figs 6, 8 and 9), are evidence of “gold dust” for an ecologically relevant trait: alleles that are likely critical to evolutionary processes but nonetheless have neither large phenotypic effects nor dramatic genomic signatures of selection [94]. Whether selection on these SNPs is due to selection for diapause or selection for correlated traits with a shared genetic basis remains unknown. However, collectively these unexceptional polymorphisms can contribute to ecologically important variation. The lack of strong genomic signals in diapause-associated SNPs relative to randomly identified SNPs suggests that perhaps a large fraction of polymorphisms in *D*. *melanogaster* could play roles in other complex traits [95] that may have experienced varying degrees of spatiotemporal selection, recent partial sweeps, and balancing selection. If many SNPs underlie ecologically relevant phenotypic variation, strong deviations from the genomic background of patterns of diversity will be the exception, not the norm. More studies of the evolutionary history of alleles underlying complex traits will be required to determine whether the pattern we observe for diapause is a general phenomenon.

### Modest heritability and strong environmental control of diapause

We find that the heritability of diapause is low but detectable (Fig 4), and we demonstrate that this heritable variation may be subject to selection under field conditions (Fig 7). We note that our estimates of individual effects of SNPs are likely underestimates due to the inclusion of a genome-wide genetic relatedness matrix in our model, which would partially correct away the effect of any given SNP (Fig 3). Like other studies [24,35,36,96], we identify temperature and nutritional exposure as two critical variables that influence diapause and extend this analysis to outbred lab and field-caught samples. Specifically, we find that diapause induction is exquisitely sensitive to extremely small differences in temperature (Fig 2), emphasizing the importance of precise thermal control and monitoring for experiments studying diapause in this species [97]. This fine-grained sensitivity suggests that even temperature variation across shelves of an incubator or at different distances from a light source could potentially influence experimental outcomes; these potential sources of thermal variation were controlled in our experiment by our use of multiple environmental chambers with nearly identical physical setup aside from temperature and photoperiod.

Our finding that diapause was not induced by short day length (but rather by long days in our experiment, Fig 2) is somewhat consistent with other studies that failed to find an effect of photoperiod on diapause in *D*. *melanogaster* [96,97]. However, some recent studies do observe short-day photoperiodic responses in diapause [90,98]. One study suggested that light conditions which mimic the natural daily variation of solar radiation can in fact generate a photoperiodic response [99]. In our study, short days under rectangular light cycles with standard white LED light did not increase diapause induction. The slightly increased diapause incidence we observe at longer photoperiods is surprising and warrants future study. Because we were able to account for temperature variation on a precise scale, we can rule out the potential of light-mediated temperature differences. Future experiments should work to disentangle the roles of temperature, wavelength and variable light intensity on photoperiodism in *D*. *melanogaster*.

### Different genetic signals underlie unique diapause phenotypes

Throughout our results, we observe different genetic signals underlying diapause scored at two different developmental stages. Diapause at stage 8 has been used as the phenotype for most studies of diapause [15,18,19,52]; however, others have argued that stage 10 is a more biologically relevant phenotype [35]. Stage 8 marks the onset of vitellogenesis [66], whereas stage 10 requires clearing a major checkpoint in the ovarian development program before proceeding to yolk-demanding stages [100]. We find an enrichment of SNPs shared between the two phenotypes, but the population genetic signals of diapause SNPs tend to be stronger for the stage 8 phenotype. The SNPs underlying stage 8 diapause vary more predictably clinally (Fig 6), are more likely to show seasonal variation (S13 Fig), and show stronger potential selection on anti-diapause alleles (Fig 8). However, we observed more dramatic seasonal variation in the stage 10 phenotype in our field study. Combined, these results suggest that the two phenotypes may be influenced by both different genetic architectures and different environmental cues, and therefore may respond differently to temporally or geographically varying selection. We also note that the genetic makeup of the mapping population appears to be critically important to the identification of SNPs associated with a polygenic trait like diapause, consistent with quantitative genetic variation in diapause being influenced by rare variants.

### Weak clinal signal in alleles underlying a strongly clinal phenotype

Diapause shows strong clinality in both North America [96] and Australia [67], with flies collected at higher latitudes showing higher diapause incidence in common gardens. This trend has not been seen in Europe, perhaps due to the high frequency in southern Europe of a pro-diapause allele that recently arose in Italy [101]. The collective effect of many weakly clinal diapause-associated alleles may be sufficient to produce clinal variation in diapause. Concordant clinal signal is seen when examining the top GWAS SNPs, with the signal driven by a cluster of linked SNPs on the X chromosome (Fig 10, S7 Table), but it is also seen genome-wide when these SNPs are excluded (S17 & S18 Figs). The latter observation is consistent with studies that have found polygenic signal across large numbers of SNPs in humans [102, but see 103] and domesticated animals [82]. However, the generally weak clinal trend suggests those SNPs with the strongest clinal signal may not always be the most ecologically relevant SNPs, at least for some phenotypes. We also note that the polygenic clinal signal was only observed in population A and both populations combined, suggesting that fewer clinally varying SNPs may underlie diapause in population B.

Our evidence of multilocus clinal selection for diapause adds to a growing literature showing evidence of spatial differentiation of loci underlying complex traits in natural populations. For example, in *D*. *melanogaster*, clinal SNPs are also enriched for SNPs influencing cuticular hydrocarbon content [104]. In gypsy moths, SNPs associated with three ecologically relevant traits have consistent clinal signals [105], and in corals, SNPs associated with heat tolerance are more common in warmer reefs and in warmer microclimates within a single reef [106]. Drought-associated SNPs also show spatial variation in European populations of *Arabidopsis thaliana* [107], and models suggest that those populations with more drought-tolerance alleles may adapt to global warming more effectively. Therefore, in addition to elucidating genetic mechanisms of local adaptation, the ability to detect polygenic adaptation of ecologically relevant traits over space is important for predicting population-level changes in response to climate change.

### Seasonal evolution of diapause may be idiosyncratic

We identified several pieces of evidence for stochastic or unpredictable seasonal variation in diapause. We observed a genetic shift towards higher diapause in the late fall in our field experiment (Fig 7) but saw little evidence for broad-scale, repeatable seasonal variation in pro-diapause alleles across multiple years (Fig 6) or multiple populations (S12 Fig). The increase in diapause in late fall may not solely be due to selection on the diapause trait itself; flies that are able to diapause are also more tolerant of cold [15], which could potentially be the phenotype under selection. If the ability to diapause makes flies more tolerant of cold, or if diapause and cold tolerance are influenced by some of the same alleles, then selection for cold tolerance in late fall could explain the increase in diapause. We also found that some individual populations show signals of seasonal variation in top diapause-associated SNPs (S13 and S14 Figs), though the direction of this variation is often the opposite of what we would predict, with pro-diapause alleles more common in the fall. Like the clinal signal, this signal appears to be driven by a cluster of SNPs near *tlk* on the X chromosome. However, unlike the clinal signal, the seasonal signal does not appear genome-wide (in all SNPs), suggesting a lack of predictable seasonal evolution of diapause-associated alleles. Interestingly, the gene *tlk*, which is involved in ovary follicle cell morphogenesis [108] and chromosome segregation [109], was also identified as a candidate gene in a genomic study of balancing selection in *D*. *melanogaster* [110]. Seasonally varying selection related to diapause or other phenotypes could contribute to this signal of balancing selection.

We propose three possible explanations for the general absence of repeatable seasonal signals in diapause-associated SNPs. First, we note that the seasonality of diapause has only been tested in mid-latitudes [19 and this study]; more southern or northern environments may not produce strong seasonal variation in diapause or other life history traits. In this case, we would not expect to see repeatable seasonal changes in diapause-associated SNPs across diverse populations. We also note that the design of our mapping experiment may have given us greater power to detect clinal signal relative to seasonal signal, due to the wide geographic spread of our starting lines and relatively few lines of known seasonal origin.

Second, the variants associated with diapause may also be favorable for stress resistance during seasons other than winter, resulting in a lack of strong spring/fall differentiation. This possibility is supported by the presence and even excess of pro-diapause alleles in central African populations, which experience seasonal dry periods and food limitation as well as cooler temperatures at high elevations [111]. To our knowledge, this study is the first to examine diapause status in field-caught flies across seasons. We observed surprisingly high incidence of diapause-like ovaries in outdoor cage-reared flies over the course of the summer and fall. While high diapause incidence was observed in December, we also observed increasing diapause throughout the summer and fall, followed by a dramatic drop in November. This finding suggests that diapause-like ovaries may be a response to general stress conditions. Indeed, starvation, heat stress, and crowding are known to cause arrest of oocyte maturation and degradation of pre-stage 10 egg chambers [85–87,112,113]. Stochastic seasonal phenotypic evolution has been documented in stick insects (*Timema cristinae*). In *Timema*, the evolution of the stripe pattern, which is under strong frequency-dependent selection, is highly predictable across seasons, whereas the evolution of color, which is under diverse selective pressures, varies unpredictably [114]. Diapause and its underlying SNPs may be subject to more complex patterns of selection due to pleiotropy and environmental plasticity, resulting in a general lack of predictably across populations and years. This possibility is further confounded by the limitations of endpoint-based sampling. While the populations were broadly sampled in “spring” and “fall”, the particular selective pressures experienced by flies in each sample may have varied dramatically between locations and years.

Lastly, seasonal evolution of diapause may require only a subset of the variants we identified, resulting in a lack of broad-scale signal across multiple sampling populations or years. This possibility could occur because only a fraction of the sites are sufficient to induce adaptive changes, or could possibly be due to false-positives and limited statistical power in both the GWAS reported here and in the seasonal surveys [53]. The seasonal model employed by [80] prioritizes SNPs that change in the same direction across many populations. The pro-diapause variants selected in different populations may differ from year to year, resulting in a lack of signal when comparing our large collection of diapause-associated variants to those SNPs with repeatable seasonal changes in allele frequency. We find evidence for this possibility in our analysis of individual populations, which revealed consistent changes in the frequency of diapause associated alleles in some, but not all, populations (S13 and S14 Figs). Likewise, polygenic adaptation to thermal regimes in *Drosophila* can use different combinations of genetic loci, with replicate populations evolving frequency changes in only subsets of the same alleles when subject to the same selective conditions [115]. However, we did observe selection for diapausing genotypes in our field data, with flies sampled in late November showing an increased genetic propensity for diapause. Given the observed phenotypic shift, we hypothesize that sequencing the late November samples would show an increase in frequency of at least some pro-diapause alleles relative to samples collected earlier in the season.

Taken together, our results confirm that North American populations carry heritable and selectable genetic variation for diapause, but the genetic basis of seasonal selection on this trait may not be repeatable from year to year or population to population. While strong evidence exists for seasonal variation in the genetic composition of local *D*. *melanogaster* populations based on pooled sequencing [53,80], the loci underlying a single quantitative trait likely do not capture the complexity of the selective forces driving these allele frequency changes. Interactions between variable environments, polygenic traits, and pleiotropic alleles may result in a lack of broad-scale seasonal signal in diapause-associated SNPs.

### Diapause may be an ancestral adaptation coopted for cold

Our results on the evolutionary history of pro-diapause alleles are consistent with recent work suggesting that diapause is in fact an ancient, polymorphic adaptation in *Drosophila* [90]. While two studies [12,89] observed negligible diapause incidence in African lines of *Drosophila*, these studies classified entire isofemale lines as diapausing or non-diapausing based on at least 50% of individuals having ovarioles developed to stage 8 [24]. Therefore, it is possible that some individuals were in diapause even if the entire line was classified as non-diapausing. More recent studies have documented quantitative variation in diapause induction among various African lines of *D*. *melanogaste*r [35,90], as well as other related species [90], using a stage 10 cutoff, but these studies did not rule out potential introgression of European haplotypes. We find that alleles promoting diapause are as common in Zambia as control SNPs identified by permutation and are not enriched in regions of European introgression. Taken together, our genetic data and previously published phenotypic results suggest that diapause at either stage may in fact be an ancestral trait that is also polymorphic in related species.

Diapause in African flies may be related to cold temperatures at mid to high elevations [116,117], or to seasonal stressors other than cold, including wet-dry seasons that influence food availability. Ancestral populations of *D*. *melanogaster* may have subsisted on marula, which only fruits for part of the year [111]. As a result, dry conditions or seasonal lack of food availability may have required intermittent diapause or estivation. Our data on diapause-associated SNPs in central Africa support this hypothesis. Interestingly, our data are also consistent with the observation by Bergland *et al* (2014) that summer-favored alleles of seasonally varying SNPs are more likely to be rare in Africa. Further, the Zambian data also consistent with the evidence of partial selective sweeps of anti-diapause alleles in North Carolina and the Northeast (Fig 9). Collectively, these data suggest diapause may in fact be favored in Zambia, and the ability to *not* diapause may be favored, at times, in temperate latitudes of North America, resulting in longer average haplotypes for anti-diapause alleles. Our findings support a model in which *D*. *melanogaster* is highly opportunistic, taking advantage of favorable conditions at any point in the year but also restricting investment in reproduction when conditions are unfavorable for offspring survival, whether due to cold, desiccation, starvation, or other stressors.

## Conclusions

Understanding the genetic basis of local adaptation has been a major goal for evolutionary biology in the genomic era [118,119]. Studying this question in *D*. *melanogaster*, which exhibits dramatic phenotypic variation across space and time and offers extensive population genomic resources, allows for a thorough exploration of the evolutionary history of alleles underlying adaptive phenotypes. Diapause is a particularly valuable trait to dissect at a genome-wide level because it underlies demonstrable life history tradeoffs and is relevant to predicting insect responses to changing climate conditions [120]. The genetic basis of variation in diapause induction has been investigated in numerous other species and ranges from a single underlying locus in *D*. *littoralis*, flesh flies and linden bugs [48,121,122], to two large effect loci involved in circadian rhythms in the European corn borer [123], to many loci in mosquitoes, face flies, and the speckled wood butterfly [124–126]. Many of these studies use traditional quantitative genetic approaches; our study is one of few to estimate the genome-wide genetic architecture of diapause via association mapping (but see [123,125]). The association mapping performed here indicates that ancestral variation in this highly polygenic trait contributes in unique ways to spatial and temporal variation in common selection pressures associated with life in temperate environments. Whether this result generalizes to other life-history, behavioral, and physiological traits that underlie local adaptation across time and space remains an open question.

Compared to many insects with hard-programmed diapauses that are triggered by photoperiod and last for months or even years [9], ovarian dormancy in *D*. *melanogaster* is short-lived, readily reversible, and highly dependent on present environmental conditions. These characteristics of diapause in *D*. *melanogaster* may be a result of the genetic architecture of the trait, with hundreds or even thousands of SNPs slightly modulating an individual’s propensity to diapause under a variety of unfavorable conditions. These loci vary somewhat predictably across space but not across time; nonetheless populations do evolve an increased propensity for diapause in unfavorable conditions, perhaps via selection of unique allelic combinations in different populations, or selection for linked traits. On a broad geographic scale, clinal differentiation of thousands of alleles of small effect results in cumulative genetic effects that produce latitudinal phenotypic variation. Despite similar patterns of diapause variation over latitudes and seasons in North America, we conclude that local adaptation relies on different patterns of selection in space and time to optimize performance in variable conditions.

## Materials and methods

### Construction of custom photoperiod chambers

We built environmental chambers (S19A and S19B Fig) from custom-cut opaque black plastic (Quality Machine Service, Waynesboro, VA). Design files for the machining of each wall are available upon request. Each chamber was controlled by a Raspberry Pi Model 3 computer with a static IP address and its own GitHub account running custom Python scripts (see https://github.com/bergland-rpi/rpi-02 for an example of scripts). To improve air circulation and prevent temperature changes associated with lights, four 92 mm computer fans (OutletPC.com) were mounted behind light-proof circular vents (4” diameter darkroom vents, midgetlouver.com). The two fans on the sides blew air in from the outside, and fans on the back and top pulled air out to create constant airflow and reduce the warming produced by lights. A custom circuit board (S19C Fig) was designed with Fritzing software (fritzing.org). The following electronic components were directly soldered to each circuit board: TSL2561 Digital Luminosity detector, SHT-31D Temperature and Humidity Sensor, MCP9808 Temperature sensor, and 74AHCT125 Quad Level Shifter. A 24-LED RGBW Natural White NeoPixel ring was connected to the circuit board with 22 AWG wire. After the initial mapping experiments, the lights were replaced with a 64-LED NeoPixel grid. All electronic components were purchased from www.adafruit.com. A custom Python script turned lights on and off for fixed photoperiods, while recording temperature, light intensity, and humidity every 60 seconds. After the initial mapping experiments, the LED lights were replaced with 64-LED RGBW Natural white NeoPixel grids for future experiments. The TSL2561 sensor was used to ensure constant light intensity across boxes for all experiments following the initial mapping.

The boxes were housed in a cold room held at 10°C (S19A Fig). However, we noticed that there was spatial variation in the actual room temperature, and we exploited this variation to expose flies to a broad range of temperatures (S3 Fig). To further modulate temperature, some boxes were outfitted with ZooMed ReptiTherm habitat heaters in one of three sizes (6x8”, 8x12”, or 8x18”). We found that these heaters increased the air temperature in the chambers by approximately 0.5, 1, and 1.5 °C respectively. We placed all fly vials horizontally on wire racks elevated 3 cm above the surface of the heaters to prevent directly warming the flies or their vials. We calibrated the temperature readings in each box by manually recording the temperature at the position of the fly vials using a high accuracy thermometer (Model EL-WIFI-DTP+; www.dataq.com) and offsetting the recorded temperatures based on this standardized reading. Quality control checks of our environmental data revealed that all chambers had consistent temperatures throughout the course of the experiment (S3A Fig) and that diurnal temperature fluctuations were limited to approximately 0.25°C (S3B and S3C Fig).

### Hybrid swarm construction and collection of individuals for phenotyping

Seventy inbred or isofemale lines spanning seven geographic/seasonal collections (S1 Table) were chosen to initiate the hybrid swarm: Rocky Ridge Orchard, Bowdoin, Maine [NCBI BioProject #PRJNA383555]; Ithaca, New York [127]; June and October collections from Linvilla Orchard, Media, Pennsylvania [128]; the Drosophila Genetic Reference Panel from Raleigh, North Carolina [79]; the Southeastern US [129], and the Bahamas [129]. Ten inbred/isofemale lines were chosen at random from each collection, and five lines were randomly assigned to each of two hybrid swarms. We reassigned approximately five lines in an attempt to balance cosmopolitan inversion frequencies across the two hybrid swarms (see “Inversion genotyping” below). Two lines (24,2 and 12LN6-24) produced an insufficient number of offspring to generate F1 crosses, so they were eliminated, and each population was initiated with 34 lines. See S1 Table for complete information about founding lines.

For each population, a total of 4 sets of 34 round-robin crosses were initiated, so each line appeared in 8 out of 136 total crosses (4 crosses used males from each line, 4 additional crosses used the females). The order of crosses was randomly generated. Fifteen virgin females and 10 males were placed in a yeasted bottle of cornmeal-molasses medium and allowed 72 hours to lay eggs. One day prior to the eclosion of the first adults, the open bottles were placed in a 2m x 2m x 2m cage (Bioquip product # 1406C). The flies were given ~5 days to eclose, and then 20 trays of fresh yeasted cornmeal-molasses food (~800 mL media in a 23 cm x 23 cm tray) were provided on day 14 to each cage to collect eggs for 48 hours. The trays were incubated at 25°C in a 12:12 light: dark light cycle, 50% relative humidity. After eclosion, 8 of 20 trays were reintroduced to the cage. For the F3, F4, and F5 generations, 20 trays were provided for 24 hours for egg laying. After egg collection, the food was removed and covered and incubated at 25 C in a 12:12 light cycle. At the F6 generation, the population size in the cages was reduced by adding only 10 food trays for 16–24 hours, and the cages were maintained in this manner for all future generations.

To collect individuals for the GWAS, yeasted bottles containing 35 mL of cornmeal-molasses media were placed in each cage for 3–6 hours to collect F4 and F5 generations. Offspring were incubated for 9–10 days at 25°C on a 12:12 light cycle. Female flies were collected under light CO_2_ anesthesia within 2 hours of eclosion (as indicated by the presence of folded wings, enlarged/pale abdomen and/or meconium). 20 flies were placed in a vial containing 10 mL of cornmeal-molasses media, and flies were placed into temperature-controlled chambers within 1 hour of collection. Flies were distributed to one of 47 chambers assigned to a photoperiod of 9, 11, 13, or 15 hours of light and a temperature varying between ~10 and 15 °C (S3 Fig). After 13–15 days, the flies were transferred to fresh food. The flies were snap frozen in liquid nitrogen after exactly 28 days and stored at -80 °C.

### Yeast supplementation experiment

We collected virgin female flies from the F20 generation of each cage as described above and placed vials in 9L:15D light cycles at 5 different temperatures. Half of the vials were supplemented with a sprinkle of live baker’s yeast, and the other half received no yeast. After four weeks, flies were snap frozen.

### Phenotyping

Flies were thawed in 70% ethanol, then transferred to a ~100 μL droplet of phosphate buffered saline for ovary dissection. Two aspects of ovary development were scored. First, the most advanced, non-egg stage of ovariole observed was recorded. We recorded stages from 6 (or less) to 11; stages 12–13 were combined into stage 11 [66]. Second, the number of fully formed eggs was counted (counted from 0–15 or scored as 15+ if more than 15 eggs were present). These phenotypes were later reduced to three binary phenotypes: diapause at stage 8 (no ovarioles observed past stage 7), diapause stage 10 (no ovarioles observed past stage 9), or diapause at stage 14 (no mature, stage 14 eggs present).

We analyzed the influence of environmental variables (temperature, photoperiod, generation, population and *Wolbachia* status (see below) using binomial generalized linear models. We used the R package *car* [130] to estimate the sum of squares explained by each variable with function *Anova()*, and then divided by the total sum of squares to estimate percent variation explained (PVE).

### DNA extraction and library preparation

After dissection, fly carcasses were placed in DNA lysis buffer (Agencourt) in a 96 well deep well plate. Forceps were cleaned with ethanol between each individual dissection to prevent DNA contamination. After completing a 96 well plate, the carcasses were lysed in a Qiagen TissueLyser using four 2-millimeter stainless steel beads. The lysates were spun down, transferred to a fresh 96 well plate, and frozen at -80 °C until further processing (see DNA extraction below). Two randomly chosen blank wells were left on each 96 well plate to verify plate identity and orientation.

DNA was extracted from 20–30 pooled flies (parental strains) or individual flies (hybrid swarm offspring) using the DNAdvance kit (Agencourt) in 96 well plates according to manufacturer’s instructions. In total, we genotyped 2,823 individual flies. An RNase treatment was added between wash 1 and wash 2, and the DNA was bound with fresh beads following the RNase treatment. Sequencing libraries were prepared using a modified Nextera protocol that permits low volumes and DNA concentrations [131]. Briefly, DNA was quantified with a Picogreen assay and normalized to 1 ng/μL with a liquid handling robot. One ng of DNA was used for library preparation with unique barcode combinations for every sample (barcodes available in supplemental file on DataDryad). For the parental lines, individual libraries were pooled to obtain equal concentrations of each line. They were sequenced in a single lane of HiSeqX with paired-end, 150 bp reads at the Hudson Alpha Institute for Biotechnology. Ten 96-well libraries of hybrid swarm individuals were pooled for each lane of sequencing so that ~940 flies and 20 blanks were combined per lane. Library sizes and quality were verified by Bioanalyzer. Hybrid swarm lanes were sequenced with three lanes of Illumina HiSeq3000 paired-end, 150 bp reads at the Oklahoma Medical Research Foundation sequencing center.

### SNP calling in parental genomes

We resequenced the genomes of all 68 parental lines to confirm their identities and compared the sequences to published sequences (see S1 Table for SRA accessions of founding line sequences). Following read merging with *PEAR* [132] and read mapping to the *Drosophila* genome version R5/dm3 with the *mem* algorithm in *bwa* [133], we called preliminary SNPs with *GATK*’s *Unified Genotyper* [134]. We found that one line (12LN6_41_B47) did not match previous sequencing, and two other lines (20,17 and 20,28) appeared to be partially contaminated (some chromosomes were accurate, others had discrepancies from previous sequencing). For these lines, we used our resequencing data as the only parental genome sequence; for the other lines, we combined our new sequence data with existing data for higher depth coverage. Individual gVCF files were generated with *HaplotypeCaller* in GATK [134] and then combined into a single parental gVCF with *CombineGVCFs*. We used randomly sampled known SNPs from the DGRP to calibrate the SNP calls with *VariantRecalibrator* and *ApplyRecalibration*. We filtered down to two sets of SNPs; one conservative set of ~1 million SNPs with the highest quality (ts_filter_level = 99) and a second more extensive set of ~3.1 million SNPs with a broader range of SNP quality (ts_filter_level = 99.9). The former set of more high-confidence SNPs was used for genome reconstruction; the latter set was further filtered as described below and used for the GWAS to allow us to test more SNPs for trait association. For reconstruction purposes, heterozygous sites in the parental lines were treated as missing data.

### *Wolbachia* status

To determine whether inbred lines and hybrids were infected with *Wolbachia*, we counted the number of sequencing reads mapping to the *Wolbachia* genome (which was part of our reference genome) and divided that number by the total number of reads mapping to chromosomes 2L, 2R, 3L, 3R, and X. This ratio produced a clearly bimodal distribution, with individuals falling into two groups: those with a ratio of substantially less than 1:1000 (0.001) and those with a ratio greater than 1:1000. We classified all individuals in the former group as *Wolbachia*-negative, and the latter individuals as *Wolbachia*-positive.

### Hybrid swarm genome reconstruction

Paired end reads were merged and mapped as described above. Bam files were then processed through our in-house genome reconstruction pipeline [63] Briefly, polymorphic reads were counted with *ASEReadCounter* in *GATK* [134] with a minimum mapping quality score of 10. *HARP* [135] was used to preliminarily call parental haplotypes. The top 14 possible founders were then used for precise genome reconstruction in *RABBIT* [136]. The output of *RABBIT* was translated to phased diploid genotypes using a custom script [63]. All lines included in the founding populations were recovered following reconstruction of the F4 and F5 generations of the hybrid swarm (S20 Fig). The median proportion of the genome derived from a line was 2.7% (range = 0.5%–9%; expected = 1/34 = 2.9%). Of over 800,000 private SNPs in the founding lines, only 109 SNPs were lost in the hybrid swarm, suggesting that virtually all founding haplotypes were recovered at least once in the sequenced hybrid swarm.

### Recombination simulation and accuracy calculations

We generated hybrid swarms *in silico* using the genotypes of our founding lines and the pipeline described in Weller and Bergland 2019 [63]. Genome sequences for each parental line were generated with *FastaAlternateReferenceMaker* in *GATK* [134] with flag *use_IUPAC_sample* to generate ambiguous bases at heterozygous sites. We simulated F4 and F5 generations for populations A and B based on recombination rates in [137] (S21 Fig). For each population and generation, we constructed 100 replicate populations of 10,000 individuals and randomly chose 5 individuals from each population. Simulated reads at 0.5X coverage for recombinant individuals were generated with *wgsim* (https://github.com/lh3/wgsim). These reads were mapped to dm3, and the simulated hybrid genomes were reconstructed as described above. To determine the accuracy of reconstruction, the known genotypes of the simulated individual were compared to the reconstructed genotypes, and the proportion of sites with identical genotypes was calculated. Sites that were missing or heterozygous in the actual founder genotypes were excluded from the accuracy calculation. We found that for both sets of founders, the majority of individuals were > 99.9% accurate (S22 Fig). We therefore predict similar levels of accuracy in our actual sequencing data.

Based on the simulations described above, we found that small genomic segments (<1 Mb) from parental haplotypes were over-represented in our reconstructed data (see S21 Fig). Haplotypes less than 1 Mb made up ~4% of all simulated genome haplotypes but made up ~20% of all reconstructed haplotypes. Therefore, any reconstructed segment less than 1Mb was masked from our genotype calls as missing data. To count the number of recombination events, any consecutive short (< 1Mb) paths were grouped together as an “unknown” parental haplotype, and that single unknown path was counted as a parental segment for recombination purposes. If a short path was surrounded by the same parental haplotype on either side, we “bridged” over it and did not count it as an additional recombination event. Following this clean up step, visual inspection revealed that the resulting genome reconstructions from both simulated and actual sequencing data more closely matched the recombination rate and haplotype size predicted by our recombination simulator (S21 Fig); however, the cleaned-up reconstructions still significantly differed from the simulated distributions of haplotype size and recombination count (Kolmogorov-Smirnov test, *P* < 8 x 10^−6^ for all tests). This discrepancy occurred in both the reconstruction of simulated data as well as the reconstruction of actual data.

### Imputation of missing data

Because the *GENESIS* software package used for the GWAS requires a complete dataset and our individuals were drawn from two populations with unique genetic composition, we performed a custom imputation of missing data within each population separately. The imputation required two steps. First, for any site at which the parental line was heterozygous, we randomly chose one allele for each offspring genotype. The first step is not a true imputation, as it is completely random and therefore allows the data to represent the range of possibilities given unknown data. After this step was performed, the remaining missing data (due to missed calls in the parental genotyping or masked short segments in the reconstruction) were imputed by taking the most likely genotype based on Hardy-Weinberg allele frequencies. This step was calculated within each hybrid population to accurately capture allele frequency differences between the two populations. We repeated this random-choice followed by Hardy-Weinberg imputation algorithm 100 times to create 100 uniquely imputed genotype sets. All analyses were carried out on all imputed data sets, and the range of values for imputations are presented for transparency of the variation that this imputation introduces. The Hardy-Weinberg imputation procedure was also used to impute allele frequencies for the population genetic analysis (see below). We eliminated samples for which more than 5% of genotypic data was imputed for the GWAS and downstream analysis (n = 83 samples).

### SNP filtering prior to association mapping

We constructed two SNP filters for mapping in our hybrid swarm population. The initial results of variant calling (see above) contained ~3.1 million SNPs. We filtered this set of SNPs with two additional filters prior to association mapping. The first filter was a strict quality control filter to identify SNPs to be used for construction of the genetic relatedness matrix (GRM). The second filter was slightly less stringent to produce a larger set of SNPs for mapping. For the strict quality control filter, we excluded SNPs that had > 10% of genotypes replaced by our imputation algorithm, an F_ST_ > 0.2 between the two populations (as calculated by *snpgdsHWE()* in *SNPRelate*), SNPs that were fixed in one population (but not the other), and SNPs with a Hardy Weinberg Equilibrium *P*-value of <10^−20^ in either population alone or the two populations combined. This filtering resulted in ~1.2 million SNPs used for GRM construction. The less stringent filter was the same as above, but allowed SNPs that were fixed in one population but not the other to remain for the GWAS. This filter resulted in ~2.2 million SNPs. After mapping, SNPs were further filtered for minor allele frequency > 0.05 in downstream analyses.

### Analysis of genetic structure of hybrid populations and parents

Principal components were calculated with the function *snpgdsPCA()* in the R package *SNPRelate* [138]. Identity by state calculations were performed with *snpgdsIBS()*. Karyotypes for each chromosome arm were inferred based on diagnostic SNPs identified in [139]. Principal component analysis of reconstructed genotypes revealed strong differentiation between populations A and B (S2 Fig), which was also evident in the identity by state genetic relatedness matrix of all individuals (S23 Fig). Overall, individuals were more related to individuals from the same swarm than from the alternate swarm. Therefore, the two hybrid swarm populations represented the full genetic diversity of their founding lines and were genetically distinct populations.

We calculated LD decay in the hybrid swarm, the hybrid swarm parents, and the DGRP using the *snpgdsLDMat()* function in *SNPRelate*. We randomly sampled 10,000 SNPs and then identified SNPs that were approximately 0.1, 0.5, 1, 5, 10, 50, 100, 500, and 1,000 kb away from the focal SNP (+/- 5%). When multiple SNPs were identified at the appropriate distance, a single SNP was randomly chosen. We performed this calculation on SNPs with minor allele frequency > 0.05. All LD analyses were performed in each hybrid swarm individually as well as the combined populations.

To calculate long-distance LD, we sampled SNPs in two ways. First, we sampled 10,000 random pairs of SNPs on opposite arms of chromosomes 2 and 3 (pairs on chromosome 2L and 2R, or 3L and 3R). Second, we sampled 30,000 random pairs of SNPs on independent chromosomes (e.g., one SNP on chromosome 2 and one SNP on chromosome 3.

### Inversion genotyping

The most likely genotypes at major cosmopolitan inversions in the parental strains and hybrid offspring were identified using diagnostic SNPs described in [139]. A binomial general linear model accounting for temperature, photoperiod, swarm, *Wolbachia*, and generation was used to test for the effect of each cosmopolitan inversion on diapause.

### Heritability estimation

We estimated narrow-sense heritability using *GCTA* [73] using the restricted maximum likelihood analysis (REML) method and the Fisher-scoring algorithm [74]. We generated a genetic relatedness matrix with the—make-grm flag in *GCTA*. We used the same covariates used for association mapping and flags—reml,—reml-alg 1, and—reml-bendV to calculate heritability for the original data as well as 1000 permutations of the sample ids. Confidence intervals were calculated as 1.96 * standard error.

### Mapping in *GENESIS*

We constructed GRMs using the *snpgdsGRM(method = “Eigenstrat”)* function in the *SNPRelate* package [138] using all SNPs that passed the mapping filter (see above) for both populations combined, as well as populations A and B separately. GRMs were generated either leaving out one entire chromosome (LOCO analysis) or by including all SNPs in the GRM (non-LOCO analysis) The appropriate GRM was then passed to *GENESIS* [140,141] with the models:

diapause~GRM+temperature+photoperiod+generation+population+Wolbachia(forbothpopulationscombined)

or

diapause~GRM+temperature+photoperiod+generation+Wolbachia(formappinginpopulationsAandBseparately)

to calculate an effect at each SNP. A binomial model was used with diapause (at either stage 10 or stage 8) scored as 1 and non-diapause scored as 0. A separate GRM and mapping result was calculated for each of the 100 imputed datasets.

### Permuted GWAS

We performed 1000 permutations of the GWAS using both populations, and 100 permutations of each single-population GWAS. Phenotypes and environmental variables were permuted together so that the environmental effects would remain constant across permutations; our permutations shuffled the sample IDs *within* each hybrid swarm population to dissociate genotype and phenotype. For each permutation, one of the 100 imputed data sets was randomly chosen for genotypes. Identical permutations were used for the stage 8 and stage 10 data and for populations A, B, and both by setting the same random number seed prior to permuting the data.

### LASSO SNPs

We used LASSO to identify a subset of informative SNPs out of the top 10,000 SNPs (ranked by *P*-value) calculated in each GWAS using the *cv*.*biglasso()* function the R package *biglasso* [142]. After filtering for a minor allele frequency of > 0.05 in the mapping population, the genotypes for the top 10,000 GWAS SNPs, ranked by *P*-value, were added to a LASSO model that included temperature, photoperiod, generation, *Wolbachia*, and 32 principal components. We ran three models: 1) environment only, 2) environment + principal components and 3) environment + principal components + 10,000 genotypes. SNPs retained in model 3 are referred to as “LASSO SNPs”. We also used *snpgdsLDMat()* from *SNPRelate* to calculate LD among LASSO SNPs.

### Receiving operator characteristic (ROC) analysis

We used the R packages *ROCR* [77] to analyze the performance of environmental data, principal components, and LASSO SNPs in predicting individual phenotypes. The predictor variables chosen in each LASSO model were used to predict the stage 8 or stage 10 diapause phenotype with the *predict()* function. We then assessed these predictions using the *performance()* function with parameters “tpr”, “fpr”, and “auc”. For plotting, the ROC curves were averaged across each analysis group (the permuted and non-permuted data for each mapping population).

### SNP annotation

We annotated the predicted effects of all SNPs identified in the hybrid swarm using SNPEff [143] with reference genome BDGP5.75 using default parameters. We grouped these annotations into 6 categories: UTR, upstream/downstream (within 5 kb), intronic, intergenic, synonymous, and non-synonymous. For each imputation and permutation of the GWAS, we calculated the percentage of LASSO SNPs and top SNPs falling into each annotation category. We then compared the percentage of SNPs in each category in the permutations to the percentage of SNPs in that category in the observed data. To assign a *P*-value, we took the median percentage of the 100 imputations of the observed data and identified its quantile rank in the permutations. In this test, *P*-values of below 0.05 are indicative of a potential de-enrichment relative to permutations, whereas *P*-values above 0.95 are indicative of an enrichment.

### Clinal and seasonal polygenic score analysis

We examined spatiotemporal variation of diapause-associated SNPs using two previously generated datasets. Bergland *et al* (2014) sampled a cline from Florida to Maine and identified SNPs with repeatable seasonal changes in allele frequency in a single Pennsylvania orchard over the course of three years. Additionally, we used the data from Machado *et al* (2019), which also sampled a cline and calculated genome-wide seasonal changes in allele frequency across 20 populations sampled from North America and Europe. We re-calculated clinal *P*-values for all SNPs in the Machado *et al* (2019) dataset using a wider latitudinal spread than originally calculated. For each location near the East Coast (Homestead, FL; Athens, GA; Hahia, GA; Eutawville, SC; Charlottesville, VA; Media, PA; State College, PA; Lancaster, MA; Ithaca, NY; Bowdoin, ME), the average allele frequency across all sampling times was calculated for each SNP. These allele frequencies were corrected for the number of individuals sequenced and read depth as in [80], then regressed to latitude in a binomial general linear model. The effect sizes (beta) from the general linear model were extracted to determine the significance and direction of the cline.

We calculated polygenic scores [103,127,144,145] using the effect sizes and signs (the Score statistic from *GENESIS* for ranked SNPs or the coefficient from the LASSO model for LASSO SNPs) from the GWAS and the clinal and seasonal effect sizes described above. We then multiplied the GWAS effect by the clinal or seasonal effect for each SNP and summed these products across all tested SNPs. These tests were polarized such that SNPs with concordant signals (i.e., those where the pro-diapause allele is more common in the north or in the spring) will result in positive values for this score, whereas SNPs with discordant signals will result in negative values. We repeated this calculation for the permutations and compared the actual average polygenic scores to those calculated in the permutations.

We used a slightly different procedure to calculate polygenic scores for individual populations from [80]. We logit-transformed the fall and spring allele frequencies for each population and then subtracted the transformed fall frequency from the transformed spring frequency. This difference in logit allele frequencies was then multiplied by the GWAS effect size and summed as described above.

### Field experiment

To test whether our hybrid swarm populations carried seasonally selectable genetic variation for diapause, we placed hybrid swarm individuals (generation 30) into six outdoor field cages near Charlottesville, VA (Top of FormBottom of Form((37°57'33.5"N 78°28'18.5"W) in early June 2018. Three cages were initiated with each hybrid swarm population. Each cage was constructed around a dwarf peach tree and fed with approximately 3 kg of Red Delicious apples and 3 kg of bananas every week until November, when feeding was reduced to biweekly. Feeding was stopped in mid-December. Each cage was founded with approximately 3,000 flies that were then allowed to reproduce in overlapping generations. We sampled these populations, as well as the indoor hybrid swarm cages multiple times during the summer and fall (“G0” samples). For some collections, we dissected the field caught females to determine whether the ovaries appeared to be in diapause.

Because other Drosophilid species, including *D*. *simulans*, infiltrated our cages, it is possible that some of the G0 females dissected were in fact *D*. *simulans*; however, we never observed more than approximately 10% *simulans* males in any collection. It is also possible that some wild *D*. *melanogaster* infiltrated the cages. At Carter Mountain Orchard, which is located approximately 2 km from our field site, we observed that *D*. *simulans* made up 60–100% of all *simulans/melanogaster* individuals over the course of our field experiment. Therefore, if at most 10% of flies in the cages were *D*. *simulans*, we conservatively estimate that at most 5% of *D*. *melanogaster* in the cages were wild flies that unintentionally entered the cages.

To determine genetic changes in the propensity to diapause, we reared the sampled flies for two generations in the lab. For the first generation, isofemale lines were established in vials and offspring males were screened to determine *D*. *melanogaster* or *D*. *simulans* identity. All *D*. *melanogaster* G1s were combined and a subset were used to establish density-controlled bottles for G2s. The G2s were then assayed for diapause using our standard diapause assay (28 days, 9L:15D, either 10.5 or 12°C). We used a binomial generalized linear model to compare diapause incidence at each collection date to the initial timepoint (June 26^th^). Weather data was downloaded from wunderground.com; station ID: KVACHARL73 (Carter Mountain, VA).

### Population genetic analysis

We calculated allele frequencies from flies collected in Zambia during phase 3 of the Drosophila Genome Nexus [91,92]. We used the full sequence FASTA files to generate allele frequencies at every SNP via custom scripts (https://github.com/alanbergland/DEST/). Using the GWAS results, we calculated the median allele frequency of the pro-diapause alleles in Zambia and compared these values to those generated using the permuted GWAS. To look for overlap with tracts of European admixture in Zambian flies, we downloaded the admixture tracts for this dataset (https://www.johnpool.net/genomes.html) and counted the number of times each SNP of interest overlapped with one of these tracts.

We used two North American fly samples to test for selective sweeps. First, we used publicly available data from the Drosophila Genetic Resource Panel (DGRP) [79]. Second, we used a set of 205 inbred lines collected from Pennsylvania and Maine (hereafter “Northern lines”). These lines were collected from Media, Pennsylvania in June and October of 2012, as well as from Bowdoinham, Maine in October 2012. The mapped reads for this dataset are available on the SRA (project number PRJNA383555). SNPs were called with *HaplotypeCaller* in *GATK* to produce gVCF files, combined with *CombineGenotypes*, and filtered for quality based on known SNPs from the DGRP. This VCF file was then used in parallel with the DGRP for population genetic analysis described below.

We calculated the integrated haplotype homozygosity score (iHS) [93] for every SNP in the DGRP or Northern lines using the R package *rehh* [146,147]. Because iHS calculation in this package requires complete genotype information, we imputed missing genotypes in each dataset based on the most likely genotype given Hardy-Weinberg equilibrium as described for the hybrid swarm above. We calculated iHS by assigning the pro-diapause allele as the “derived” allele at each SNP based on the Score statistic from GENESIS. For each set of SNPs of interest, we calculated the median iHS and compared these values to those generated using the permuted GWAS.

### Statistical analysis and plotting

For analyses comparing population genetic measures between imputations and permutations, we employed the following statistical analysis. We calculated the 2.5% and 97.5% quantiles of the permuted data, and then counted how many imputations had a test statistic that exceeded these limits. If more than 50% of imputations were either higher than the 97.5% quantile or lower than the 2.5% quantile, we considered this a significant result because the majority of imputations are outliers relative to the permutations. We report the percentage of imputations that are outliers, if significant, on the indicated plot. All analysis was performed using R versions 3.3 to 3.5 [148]. In addition to the aforementioned packages, the following packages were used for general analysis and plotting: *ggplot2* [149], *cowplot* [150], *data*.*table* [151], *foreach* [152], *doMC* [153], *ggbeeswarm* [154], *lubridate* [155], *maps* [64], and *viridis* [156]. Computational analysis was performed on the Rivanna high performance computing cluster at the University of Virginia.

## Supporting information

S1 Fig Principal component analysis of genetic diversity of parental lines. Principal components (PC) were calculated for (A) all parental lines, (B) northern and southern lines (excluding the DGRP from North Carolina), and (C) lines with known spring and fall collection dates. PCs were calculated genome-wide (all) or for each chromosome arm separately.
(PDF)

S2 Fig Principal components analysis of hybrid swarm populations, including parental lines. PC1 and PC2 are plotted for all chromosomes combined (all), as well as each individual chromosome arm, and are color coded by population. Dark shapes indicate parental lines, while transparent shapes indicate individual hybrids. Circles indicate an individual that is either heterozygous or homozygous for one of the cosmopolitan inversions on that chromosome.
(PDF)

S3 Fig Environmental control chambers maintain constant temperatures with little temperature change from lights. (A) Average temperature of each box across a 24 hour day, separated by photoperiod into vertical facets. Each line represents the mean temperature of a single box recorded every 60 s for ~6 weeks. (B). Average temperature of each box when lights are off and lights are on, color coded by photoperiod. (C). Difference in average temperature when lights are on and lights are off. Each point represents one box.
(PDF)

S4 Fig Diapause phenotypes differ between generations and cages. Points indicate individual phenotypes (1 = diapause, 0 = non-diapause). Lines represent binomial models for each group. (A) F4s had uniformly higher diapause incidence than F5s, regardless of phenotype assessed (general linear model, *P* < 2 x 10^−16^ for all). (B) The two hybrid swarms were also significantly different for all three diapause phenotypes. For stage 8, population B had generally higher diapause incidence, while cage A had higher incidence for stages 10 and 14 (general linear model, *P* = 0.0006, *P* = 0.01, *P* = 1.75 x 10^−5^, respectively).
(PDF)

S5 Fig Live yeast supplementation decreases diapause incidence across temperatures. Advanced generation hybrid swarm individuals were exposed to diapause inducing conditions (9L:15D, 10–14°C) with or without a sprinkling of live baker’s yeast on the surface of the food. Points represent individuals with jitter added for visual clarity (1 = diapause, 0 = nondiapuse); lines represent binomial models for each population and treatment. The presence of yeast decreased diapause at all thresholds scored (binomial general linear model, *P* < 2 x 10^−16^ for all stages). There was a significant effect of population for all three phenotypes, with cage A showing higher diapause incidence than cage B, regardless of temperature or yeast treatment (*P* = 0.03, *P* = 3.3 x 10^−6^, *P* = 7.1 x 10^−7^).
(PDF)

S6 Fig Genome reconstructions for individual hybrids from populations A (top) and B (bottom). Each horizontal line represents one haploid chromosome (n = 2823 diploid individuals total). Data are separated by chromosome arm (horizontal) and population (vertical). Grey indicates regions that were masked due to short inferred parental haplotypes (roughly 1.2% of sequences). Each founding line is represented by a different color.
(PDF)

S7 Fig λ_GC_ for individual chromosomes. The genomic inflation factor (GIF) was calculated for each chromosome separately in all three mapping populations. Colors illustrate 100 imputations of the observed data; grey indicates permutations (100 permutations for A and B, 1000 permutations for the combined data). Points indicate the median, bars illustrate the 2.5%-97.5% quantiles.
(PDF)

S8 Fig LASSO SNPs are generally unlinked. LD heatmaps showing R^2^ for LASSO SNPs from 10 imputations of the original ordering of the data (A) and 10 random permutations (B). LASSO SNPs for stage 10 diapause from the combined A+B mapping population were used to generate this figure.
(PDF)

S9 Fig Number of SNPs chosen by LASSO. The number of LASSO SNPs was counted for each imputation or permutation of each phenotype in each mapping population. Points represent the median; bars extend to the 2.5% and 97.5% quantiles. Colors represent 100 imputations of the observed data; grey bars represent permutations (100 permutations for A and B, 1000 permutations for both). Numbers represent the percent of imputations that exceed the 97.5% quantile of the permutations, if that number is greater than 50%.
(PDF)

S10 Fig Number of diapause-associated SNPs shared between mapping populations and phenotypes. (A). For each imputation and permutation, the number of SNPs shared between populations A and B was counted for various sets of GWAS SNPs. Pink indicates 100 imputations of the actual data, grey indicates 100 permutations. Points represent the median and error bars extend to the 2.5% and 97.5% quantiles. (B) For each imputation and permutation, the number of SNPs shared between stage 8 and stage 10 diapause was counted. Grey points represent 100 permutations of populations A and B; 1000 permutations of both populations combined. Colored points represent 100 imputations of the actual data. Numbers represent the percent of imputations that exceed the 97.5% quantile of the permutations, if that number is greater than 50%.
(PDF)

S11 Fig Previously identified diapause-associated variants have no effect in this study. (A-B). A 1 bp indel that creates an alternate translation start site in *timeless* (*tim*) does not affect diapause in the full dataset or either individual population after correcting for temperature for diapause at stage 8 (A) or stage 10 (B). (C-D) An intronic SNP in *couch potato (cpo)* also has no effect in the full dataset or either individual population after correcting for temperature for diapause at stage 8 (C) or stage 10 (D). General linear model *P*-values for genotype are greater than 0.05 for all models, except for *tim* in population B, stage 8 (*P* = 0.0189).
(PDF)

S12 Fig Polygenic score test for data from Machado *et al*, 2019. A) Polygenic scores calculated by multiplying clinal effect size and GWAS effect sizes for each SNP and summing across all SNPs, LASSO SNPs, the top 0.01% of the GWAS, and the top 0.1% of the GWAS. Effect sizes are polarized such that positive numbers indicated pro-diapause alleles are more common in the north. Data are shown with a point for the mean and error bars extending to the 2.5% and 97.5% quantiles. Colored points indicate actual data for 100 imputations of each mapping population; grey points indicate the distribution for permutations. B) Polygenic scores calculated for seasonal data by multiplying seasonal betas and GWAS effect sizes, polarized so that pro-diapause and spring are positive. Numbers represent the percent of imputations that exceed the 97.5% quantile of the permutations, if that number is greater than 50%.
(PDF)

S13 Fig Individual population polygenic score test for stage 8 diapause in populations collected by Machado *et al* (2019). The GWAS or LASSO effect size was multiplied by the logit-transformed change in allele frequency from spring to fall in 20 populations. These products were then summed across all SNPs of interest for each mapping population. Populations are ordered by increasing latitude and year. Points represent median, error bars represent 2.5% and 97.5% quantiles. Grey points/bars are permutations; colors represent 100 imputations of the observed data. Numbers represent the percent of imputations that are below the 2.5% quantile or exceed the 97.5% quantile of the permutations, if that number is greater than 50%.
(PDF)

S14 Fig Individual population polygenic score test for stage 10 diapause in populations collected by Machado *et al* (2019). The GWAS or LASSO effect size was multiplied by the logit-transformed change in allele frequency from spring to fall in 20 populations. These products were then summed across all SNPs of interest for each mapping population. Populations are ordered by increasing latitude and year. Points represent median, error bars represent 2.5% and 97.5% quantiles. Grey points/bars are permutations; colors represent 100 imputations of the observed data. No imputations significantly exceed the 2.5% or 97.5% quantiles of the permutations.
(PDF)

S15 Fig Field cages used to study diapause evolution under natural conditions. Summer (A) and winter (B) views of the experimental orchard with peach trees enclosed in mesh cages. (C) Flies feeding on yeasted fruit. (D) Winter view of compost pile in more advanced stage of decomposition. Photos in B and D were taken on December 10^th^, 2018, the final collection point in Fig 6. Surviving *D*. *melanogaster* were recovered from the cages on this day, despite heavy snow and several days of sub-freezing temperatures.
(PDF)

S16 Fig GWAS SNPs are not enriched in tracts of admixture between European and Zambian flies. Each set of SNPs was intersected with the admixture tracts of European/Zambian admixture, and the proportion of SNPs found within at least one admixture tract was calculated for each mapping population. Points represent median, error bars represent 2.5% and 97.5% quantiles. No imputations significantly exceed the 2.5% or 97.5% quantiles of the permutations. Grey points/bars are permutations; colors represent 100 imputations of the observed data.
(PDF)

S17 Fig Clinal polygenic score and IHS excluding top-ranked SNPs for stage 8 diapause. Top quantile-ranked SNPs were excluded from the analysis to determine whether they influence the genome-wide signal for clinal polygenic score based on Bergland *et al* 2014 (A), IHS in the DGRP (B) or IHS in Northern populations (C). Points represent median, error bars represent 2.5% and 97.5% quantiles. Grey points/bars are permutations; colors represent 100 imputations of the observed data. Numbers represent the percent of imputations that are below the 2.5% quantile or exceed the 97.5% quantile of the permutations, if that number is greater than 50%.
(PDF)

S18 Fig Clinal polygenic score and IHS excluding SNPs near *tlk* for stage 8 diapause. All SNPs in the region chrX:3,600,000–3,700,000 were excluded from the analysis, which is otherwise identical to that shown in Figs 6 and 9, for clinal polygenic score based on Bergland *et al* 2014 (A), IHS in the DGRP (B) or IHS in Northern populations (C). Points represent median, error bars represent 2.5% and 97.5% quantiles. Grey points/bars are permutations; colors represent 100 imputations of the observed data. Numbers represent the percent of imputations that are below the 2.5% quantile or exceed the 97.5% quantile of the permutations, if that number is greater than 50%.
(PDF)

S19 Fig Photoperiod chambers. A) Array of chambers in cold room. B) Side view illustrating fan and externally mounted Raspberry Pi with ethernet connection. C) Interior view illustrating heating element (bottom), light-proof vents (sides), LED lights (top) and circuit board (top left). D) Fritzing layout for custom printed circuit board (PCB). File available upon request. E) Circuit board connected to Raspberry Pi computer via 40 pin ribbon cable.
(PDF)

S20 Fig All founding lines are represented in F4 and F5 hybrid swarms. The percent contribution of each founding line in each chromosome arm was calculated for swarm A (left) and B (right). F4s are shown with positive values, and F5s are mirrored with negative values below. Color coding corresponds to geographical origin of the lines. Dashed grey lines indicate the expected contribution of each line (1/34 = 2.9%) under perfectly even admixture.
(PDF)

S21 Fig Haplotype size and recombination distributions for reconstructed F4 and F5 genomes. (A-B) The initial genome reconstruction of sequencing data (blue, A) shows an excess of short (~10,000–1,000,000 bp) haplotypes relative to simulated F4 and F5 populations (grey bold line). This excess also occurs in reconstructions of simulated hybrid swarm reads (B, purple). Cleaning up the reconstruction data by combining adjacent short (< 1 Mb) haplotypes into unknown haplotypes and dropping singleton short haplotypes results in a distribution of haplotype sizes that more closely match the simulations for both empirical data (red, A) and simulated data (green, B). (C-D) Raw reconstructions have an excess of recombination events (blue and purple) relative to simulated data (grey). The cleanup procedure results in recombination numbers on par with the simulated data (red and green).
(PDF)

S22 Fig Accuracy of simulated genome reconstructions. Hybrid F4 and F5 individuals were simulated from the founding lines for populations A and B using a custom script, and 0.5X coverage sequencing reads were generated with *wgsim*. These simulated reads were passed through the genome reconstruction pipeline, and the reconstructed genotypes were compared to the original simulated individual. Accuracy was determined as the proportion of all sites with an exact match between the reconstructed genotype and actual genotype. The vast majority of individuals have an accuracy of >99%, though accuracy is higher in population A than population B. Note logarithmic scale of y-axis.
(PDF)

S23 Fig Identity-by-state genetic relatedness matrix for all hybrid swarm individuals. Individuals are ordered by population (A on left, B on right) and sorted by identity by state. IBS was calculated using an LD-pruned set of ~63,000 SNPs with allele frequencies > 0.05. Individuals are generally more closely related to other individuals in the same population.
(TIF)

S1 Table SRA and collection information for previously sequenced parental lines. (XLSX)

S2 Table Variance decomposition for variables influencing diapause. (PDF)

S3 Table *P*-values from general linear model of the effects of cosmopolitan inversions on diapause in each population. (PDF)

S4 Table Functional annotations in diapause-associated SNPs. For each class of variant, the percentage of diapause-associated SNPs assigned to that variant type was quantified. The percentage reported is the median of 100 imputations.
(PDF)

S5 Table Enrichment of functional annotations in diapause-associated SNPs. For each class of variant, the proportion of diapause-associated SNPs assigned to that variant type was quantified for each imputation and permutation. The numerical values are the quantile rank of the median of the 100 observed imputations relative to the distribution of the permutations. Quantile ranks below 5% and above 95% (i.e. those values that are de-enriched or enriched relative to permutations) are highlighted in bold.
(PDF)

S6 Table List of LASSO SNPs, top 0.01% SNPs, and top 0.1% SNPs along with annotations. (XLSX)

S7 Table Position, annotation, and population genetic statistics for top 0.01% SNPs in the *tlk* region. *P*-values are based on mapping stage 8 diapause in both populations. Number of imputations refers to the number of imputations in which the SNP fell in the top 0.01%, and all average values are calculated across these imputations. Average clinal polygenic score is based on Bergland *et al* 2014.
(XLSX)
