# Supplementary figures and images for: Unique genetic signatures of local adaptation over space and time for diapause, an ecologically relevant complex trait, in Drosophila melanogaster
Source: PLoS Genet. 2020 Nov 20;16(11):e1009110. doi: 10.1371/journal.pgen.1009110 (PMC7717581; doi:10.1371/journal.pgen.1009110)

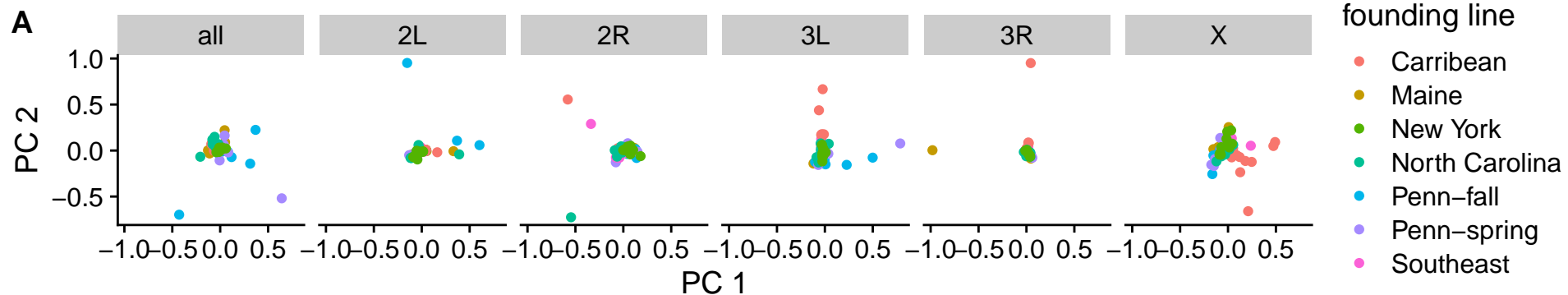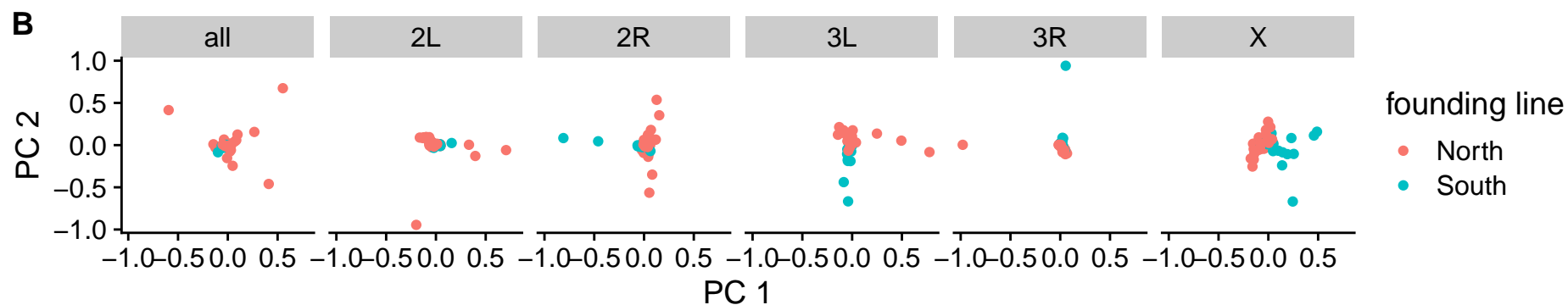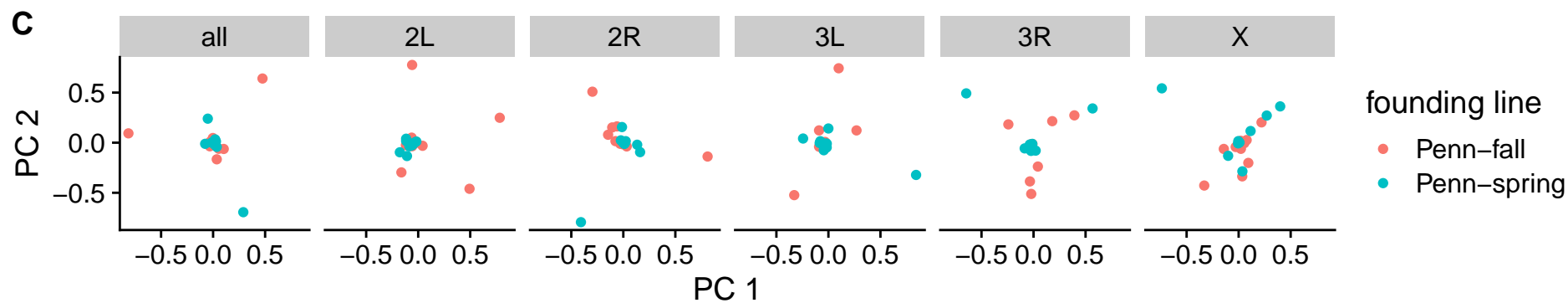

Supplement: S1 Fig — Principal components (PC) were calculated for (A) all parental lines, (B) northern and southern lines (excluding the DGRP from North Carolina), and (C) lines with known spring and fall collection dates. PCs were calculated genome-wide (all) or for each chromosome arm separately. (PDF) [file pgen.1009110.s001.pdf]

PC2

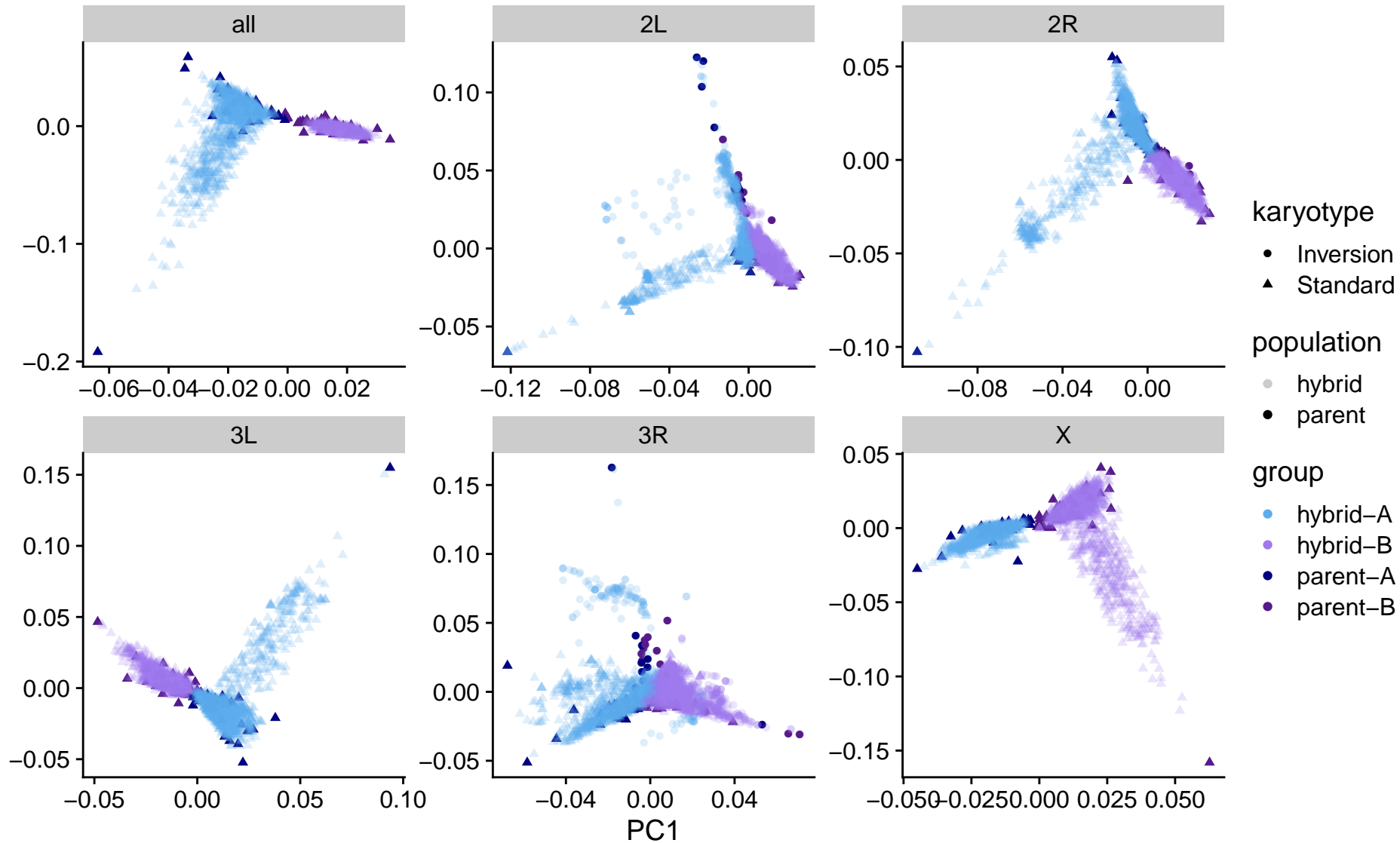

Supplement: S2 Fig — PC1 and PC2 are plotted for all chromosomes combined (all), as well as each individual chromosome arm, and are color coded by population. Dark shapes indicate parental lines, while transparent shapes indicate individual hybrids. Circles indicate an individual that is either heterozygous or homozygous for one of the cosmopolitan inversions on that chromosome. (PDF) [file pgen.1009110.s002.pdf]

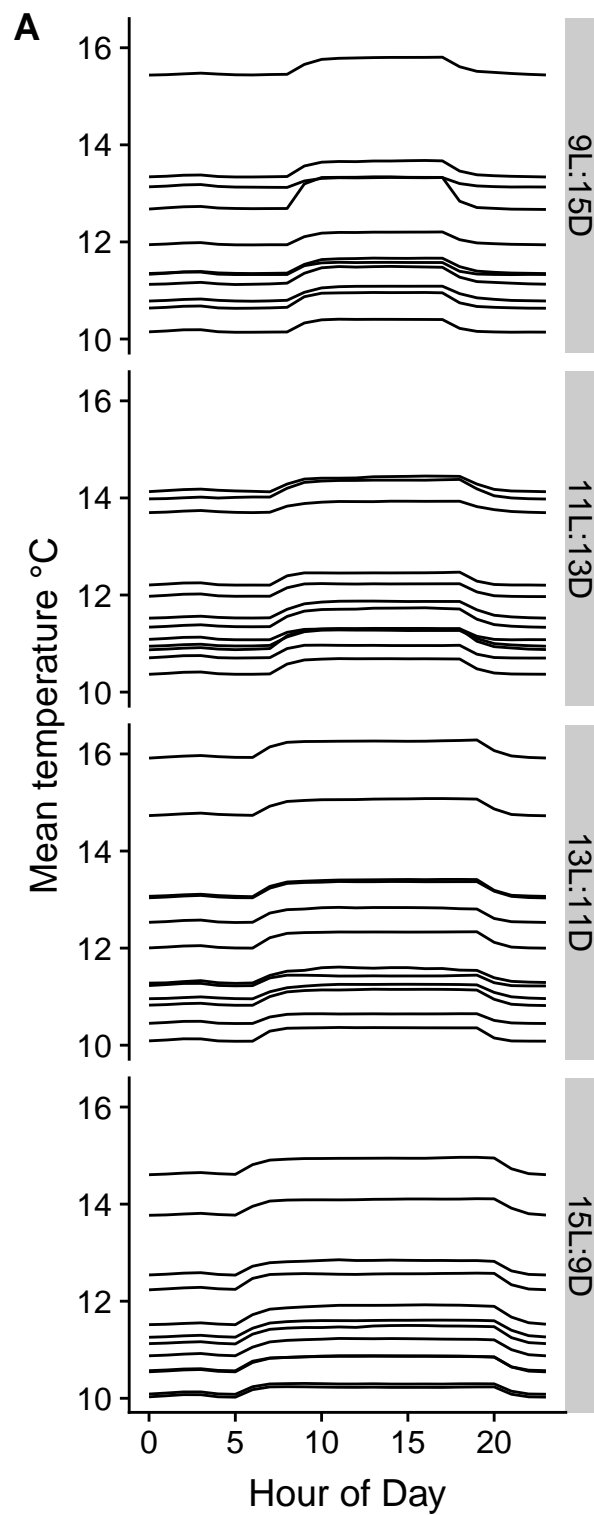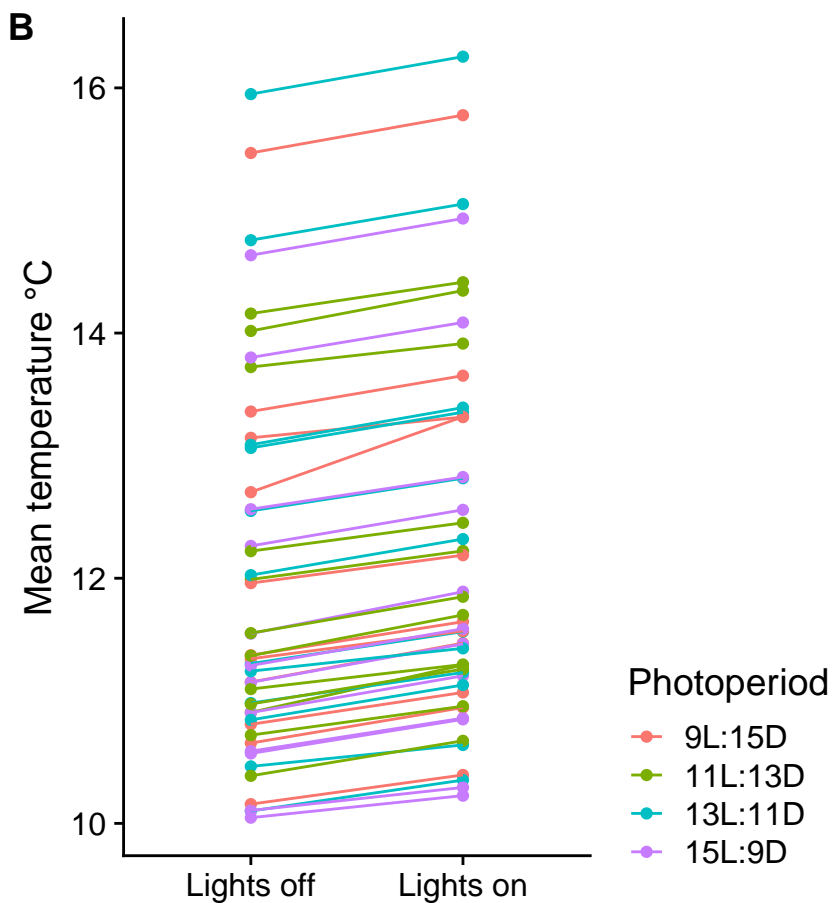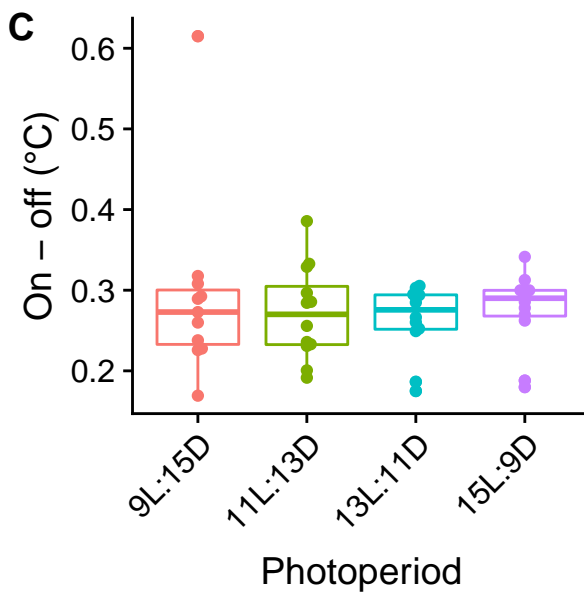

Supplement: S3 Fig — (A) Average temperature of each box across a 24 hour day, separated by photoperiod into vertical facets. Each line represents the mean temperature of a single box recorded every 60 s for ~6 weeks. (B). Average temperature of each box when lights are off and lights are on, color coded by photoperiod. (C). Difference in average temperature when lights are on and lights are off. Each point represents one box. (PDF) [file pgen.1009110.s003.pdf]

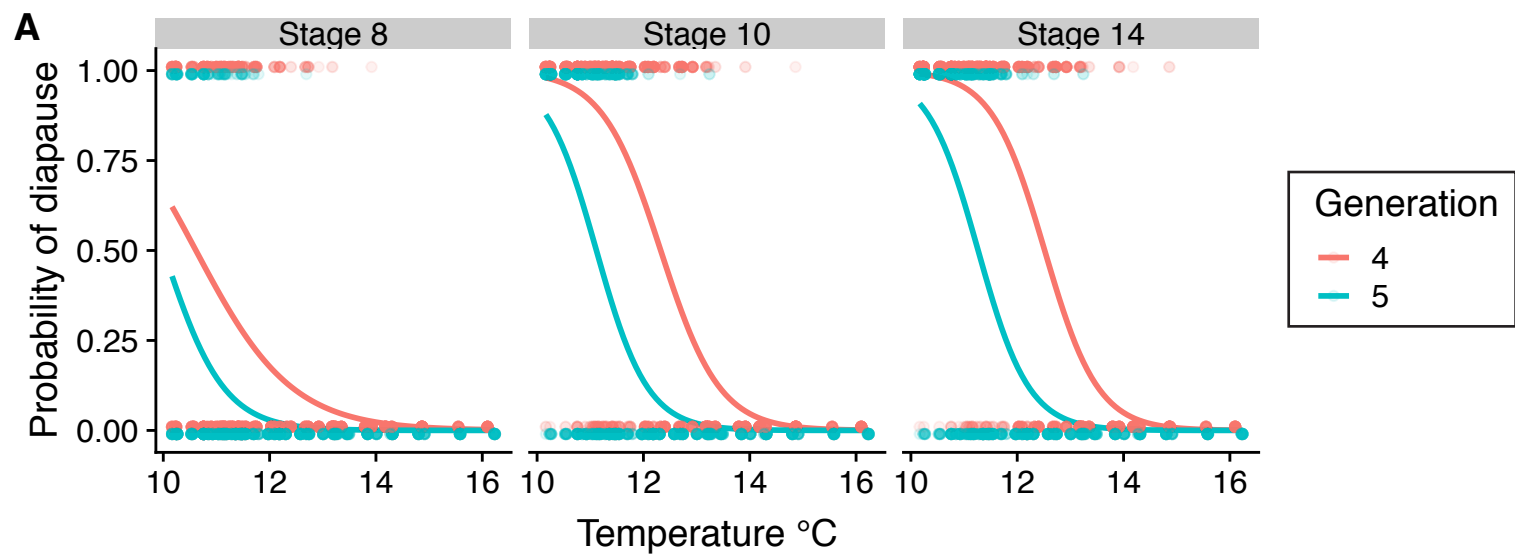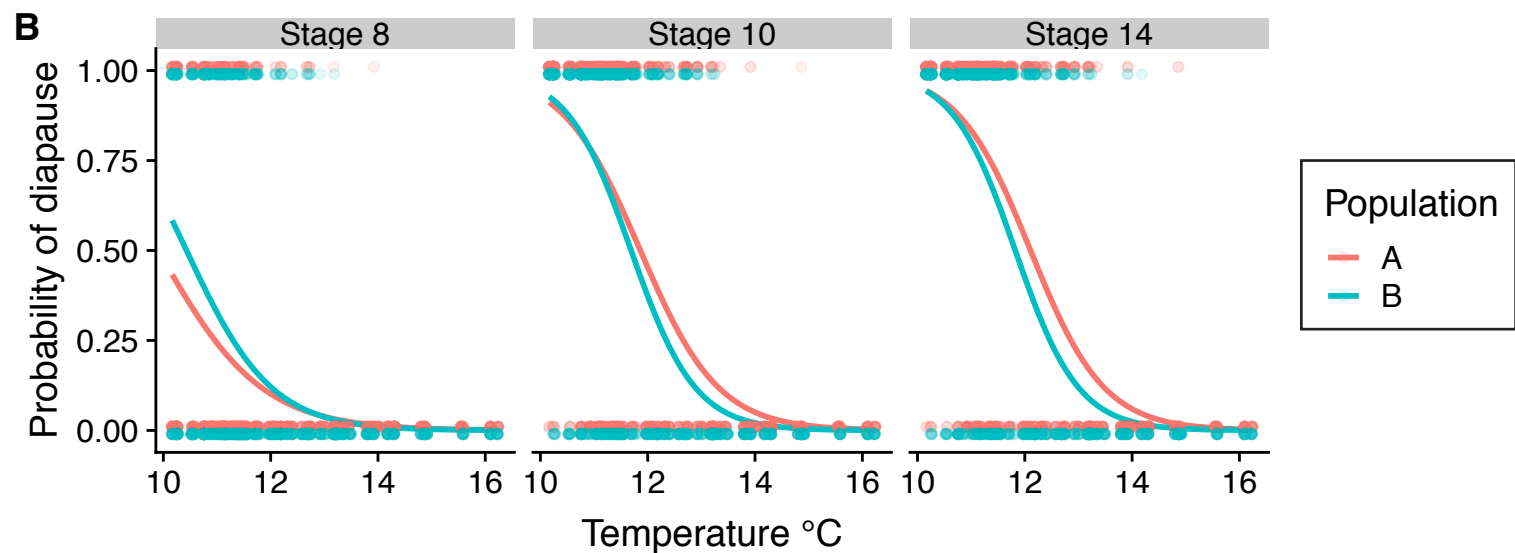

Supplement: S4 Fig — Points indicate individual phenotypes (1 = diapause, 0 = non-diapause). Lines represent binomial models for each group. (A) F4s had uniformly higher diapause incidence than F5s, regardless of phenotype assessed (general linear model, P < 2 x 10^−16^ for all). (B) The two hybrid swarms were also significantly different for all three diapause phenotypes. For stage 8, population B had generally higher diapause incidence, while cage A had higher incidence for stages 10 and 14 (general linear model, P = 0.0006, P = 0.01, P = 1.75 x 10^−5^, respectively). (PDF) [file pgen.1009110.s004.pdf]

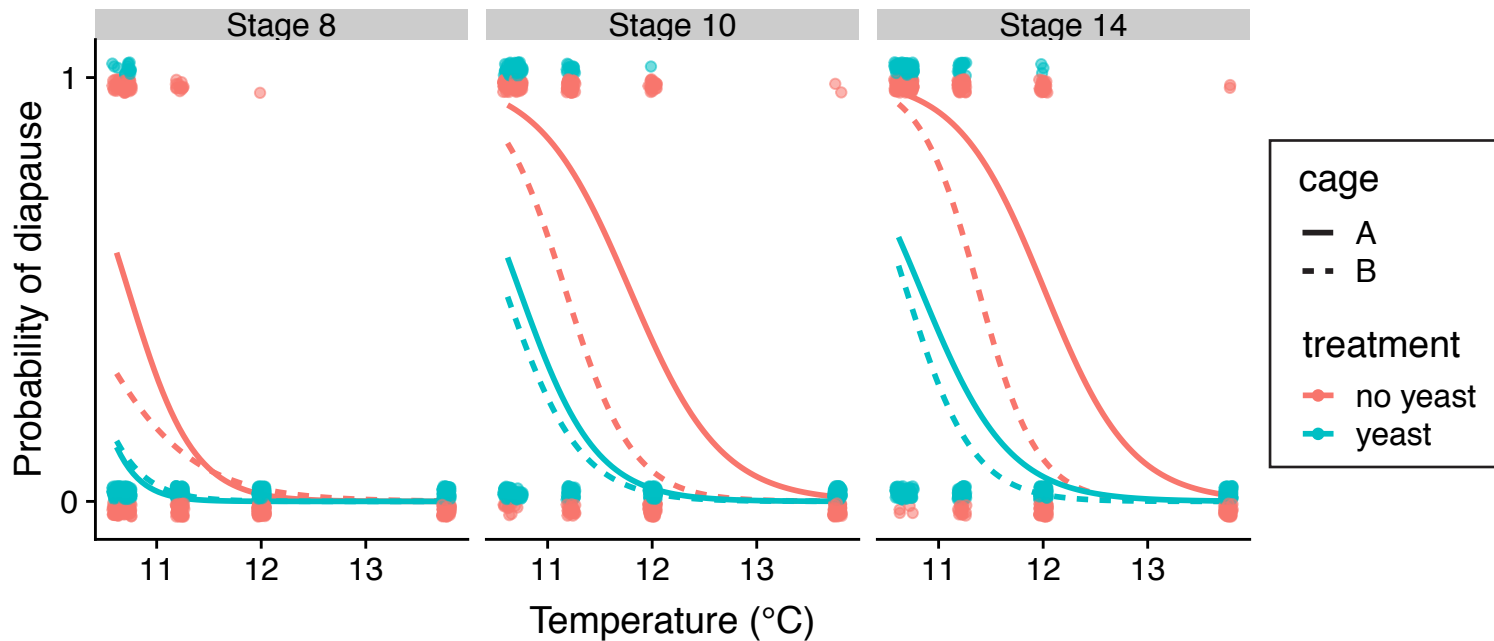

Supplement: S5 Fig — Advanced generation hybrid swarm individuals were exposed to diapause inducing conditions (9L:15D, 10–14°C) with or without a sprinkling of live baker’s yeast on the surface of the food. Points represent individuals with jitter added for visual clarity (1 = diapause, 0 = nondiapuse); lines represent binomial models for each population and treatment. The presence of yeast decreased diapause at all thresholds scored (binomial general linear model, P < 2 x 10^−16^ for all stages). There was a significant effect of population for all three phenotypes, with cage A showing higher diapause incidence than cage B, regardless of temperature or yeast treatment (P = 0.03, P = 3.3 x 10^−6^, P = 7.1 x 10^−7^). (PDF) [file pgen.1009110.s005.pdf]

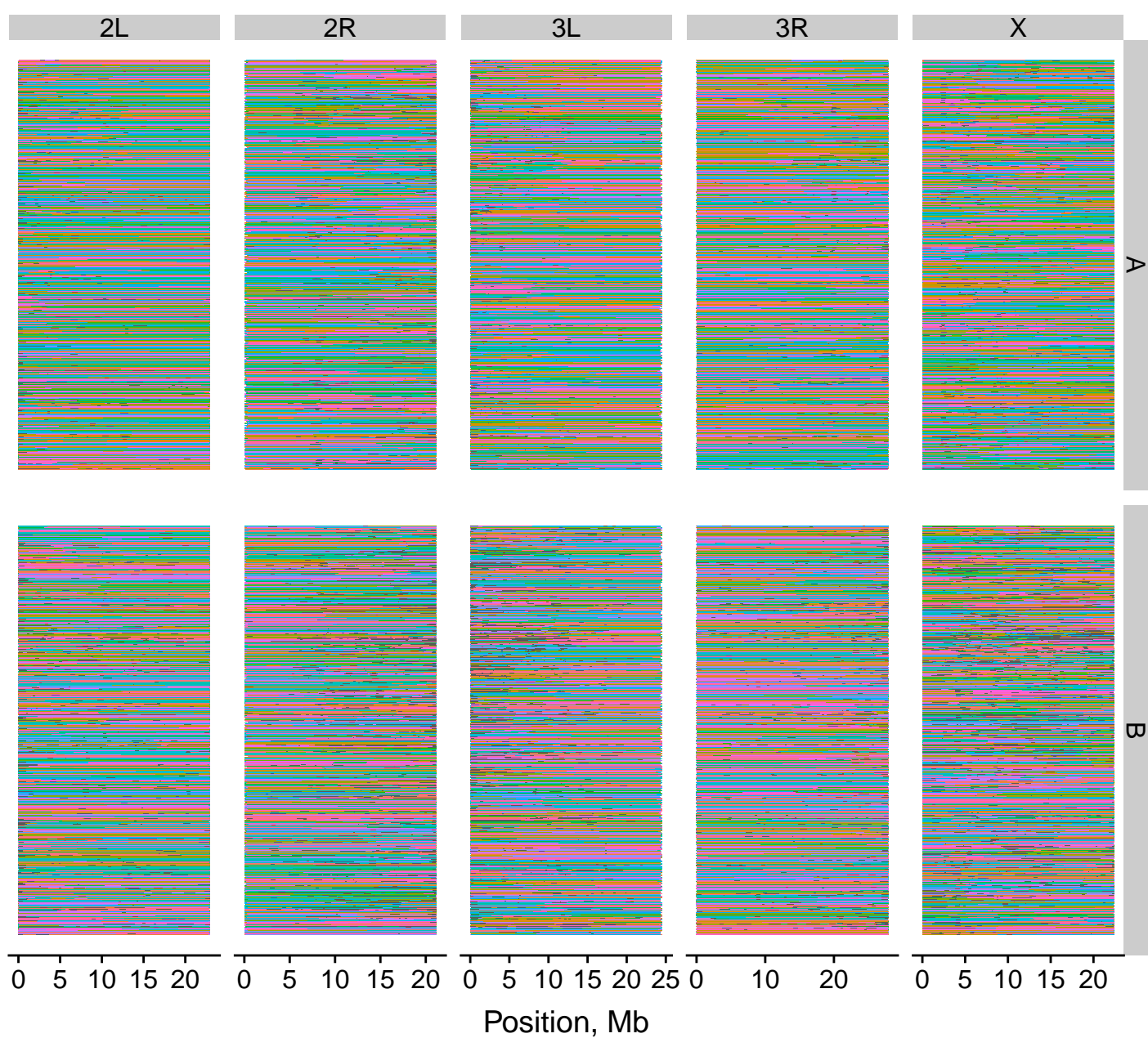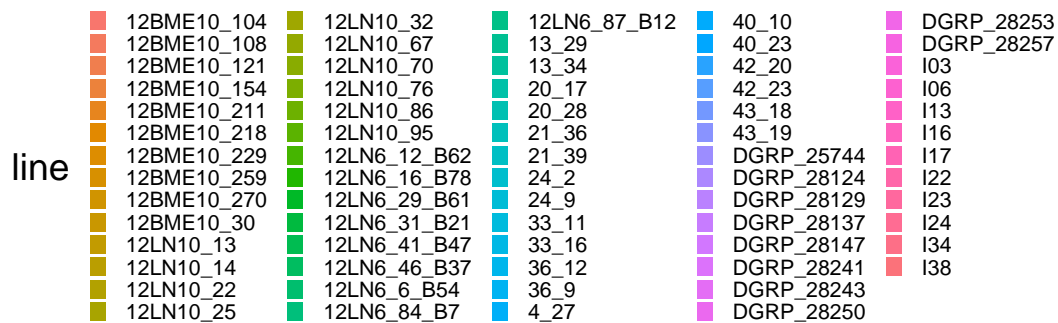

Supplement: S6 Fig — Each horizontal line represents one haploid chromosome (n = 2823 diploid individuals total). Data are separated by chromosome arm (horizontal) and population (vertical). Grey indicates regions that were masked due to short inferred parental haplotypes (roughly 1.2% of sequences). Each founding line is represented by a different color. (PDF) [file pgen.1009110.s006.pdf]

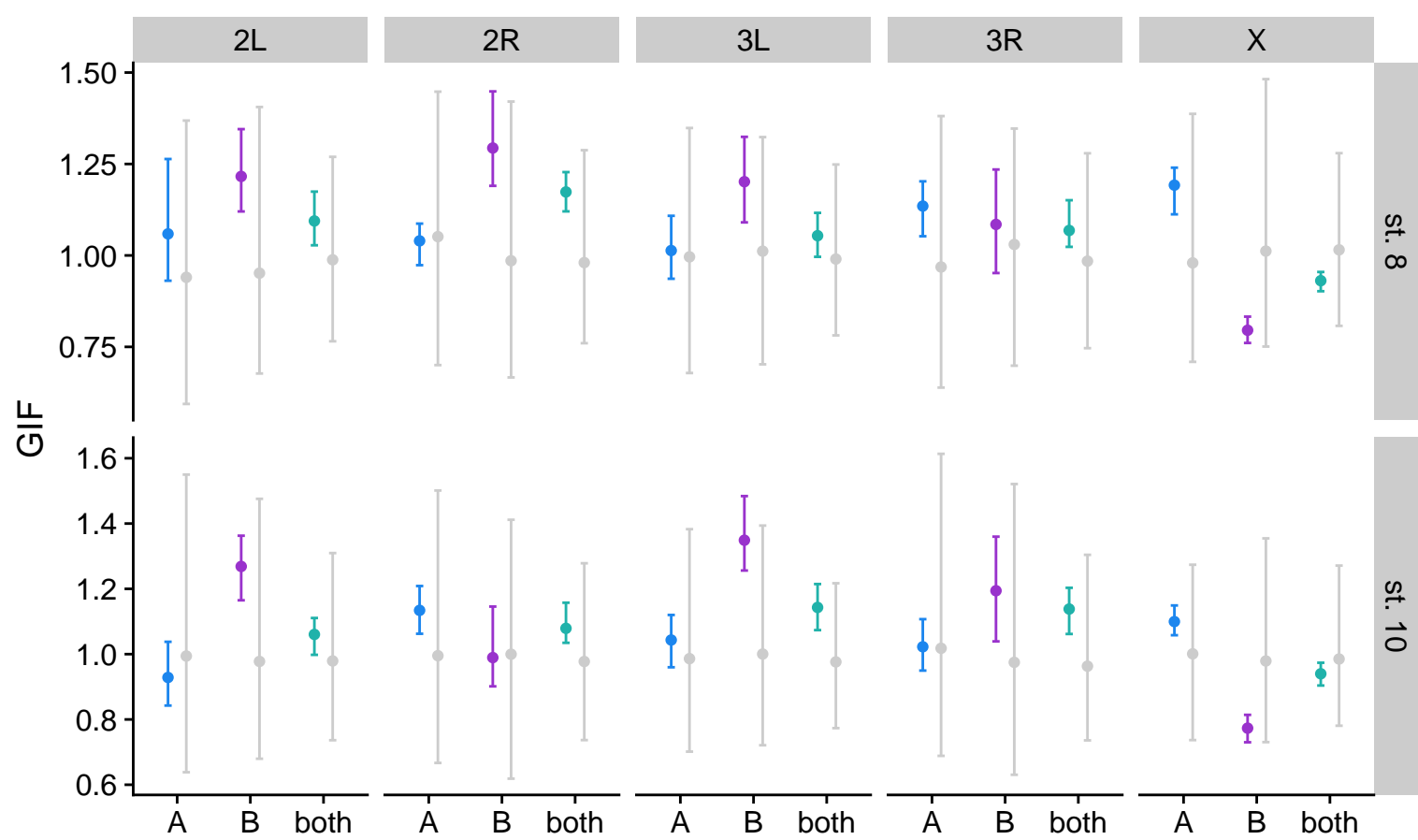

Supplement: S7 Fig — The genomic inflation factor (GIF) was calculated for each chromosome separately in all three mapping populations. Colors illustrate 100 imputations of the observed data; grey indicates permutations (100 permutations for A and B, 1000 permutations for the combined data). Points indicate the median, bars illustrate the 2.5%-97.5% quantiles. (PDF) [file pgen.1009110.s007.pdf]

**A**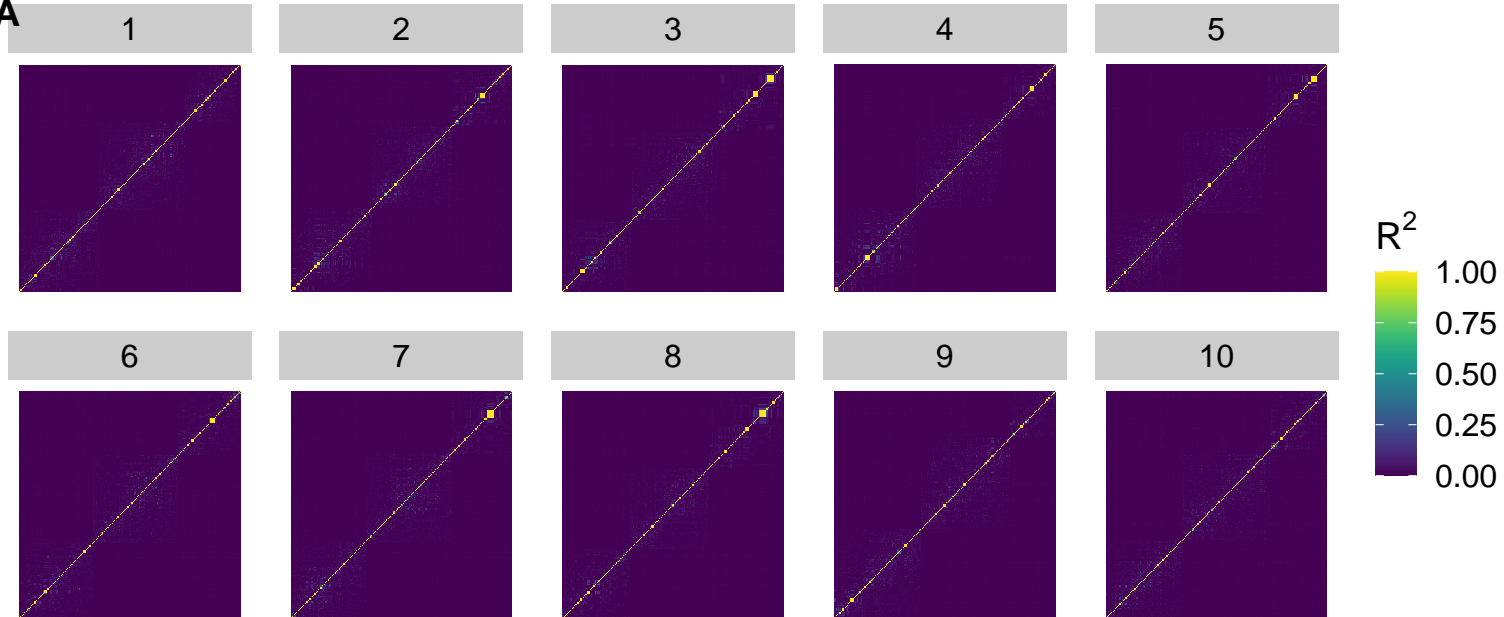**B**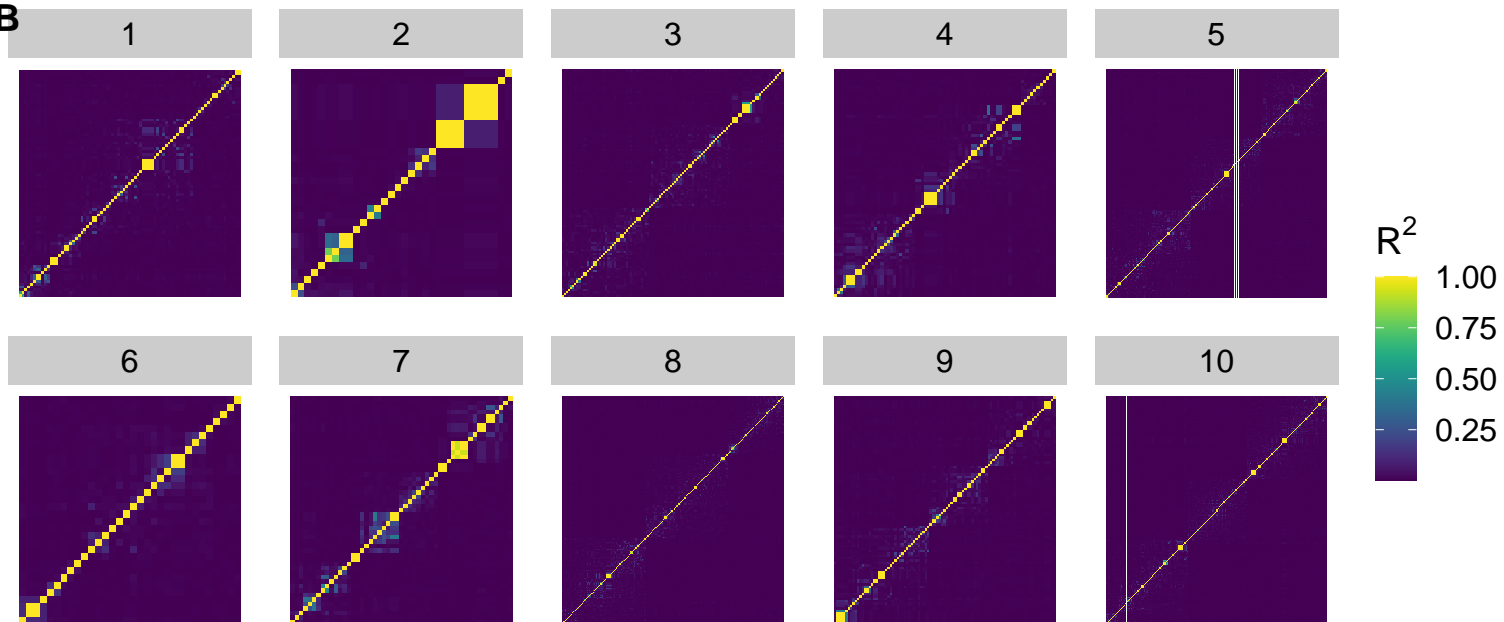

Supplement: S8 Fig — LD heatmaps showing R2 for LASSO SNPs from 10 imputations of the original ordering of the data (A) and 10 random permutations (B). LASSO SNPs for stage 10 diapause from the combined A+B mapping population were used to generate this figure. (PDF) [file pgen.1009110.s008.pdf]

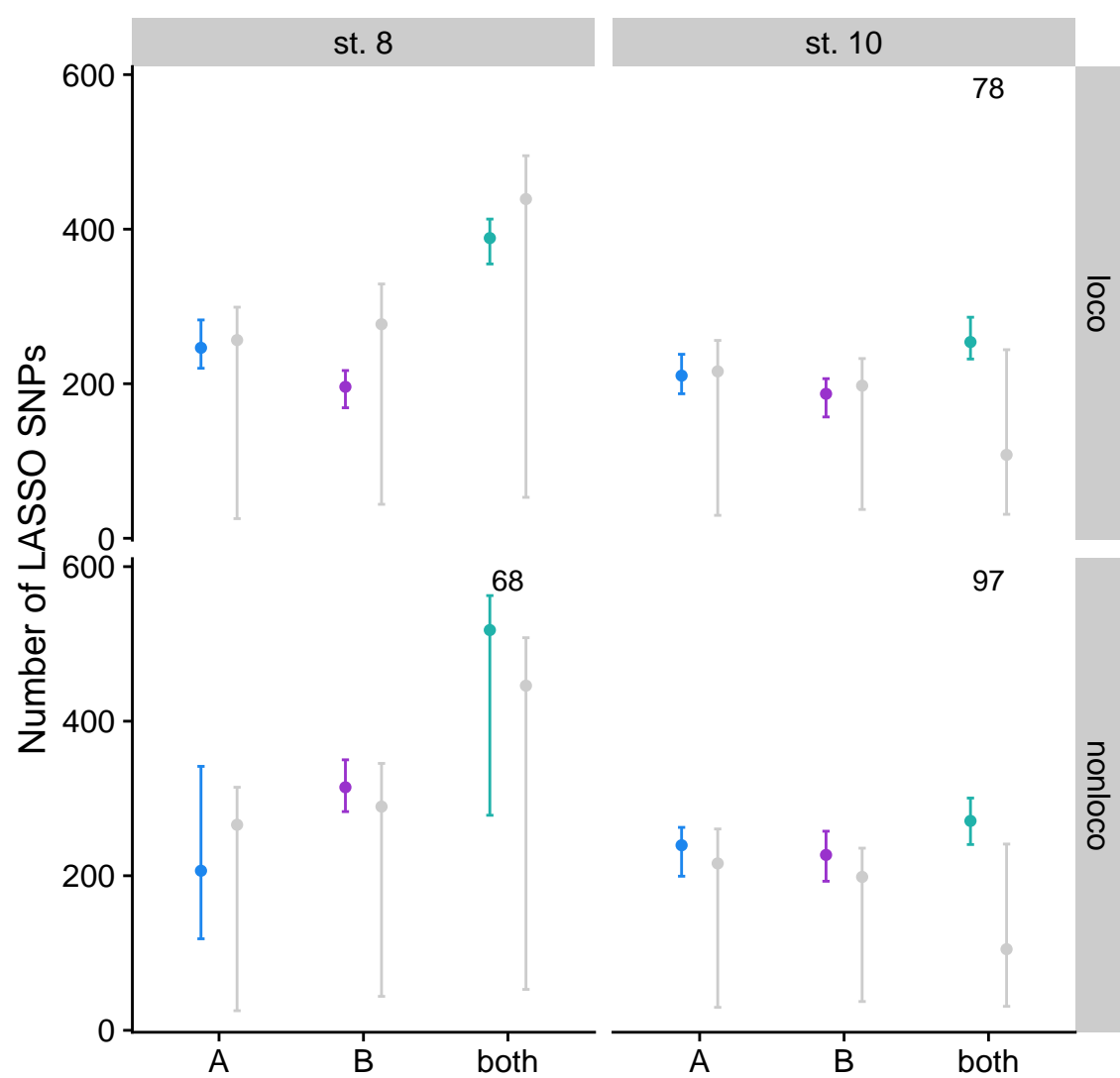

Supplement: S9 Fig — The number of LASSO SNPs was counted for each imputation or permutation of each phenotype in each mapping population. Points represent the median; bars extend to the 2.5% and 97.5% quantiles. Colors represent 100 imputations of the observed data; grey bars represent permutations (100 permutations for A and B, 1000 permutations for both). Numbers represent the percent of imputations that exceed the 97.5% quantile of the permutations, if that number is greater than 50%. (PDF) [file pgen.1009110.s009.pdf]

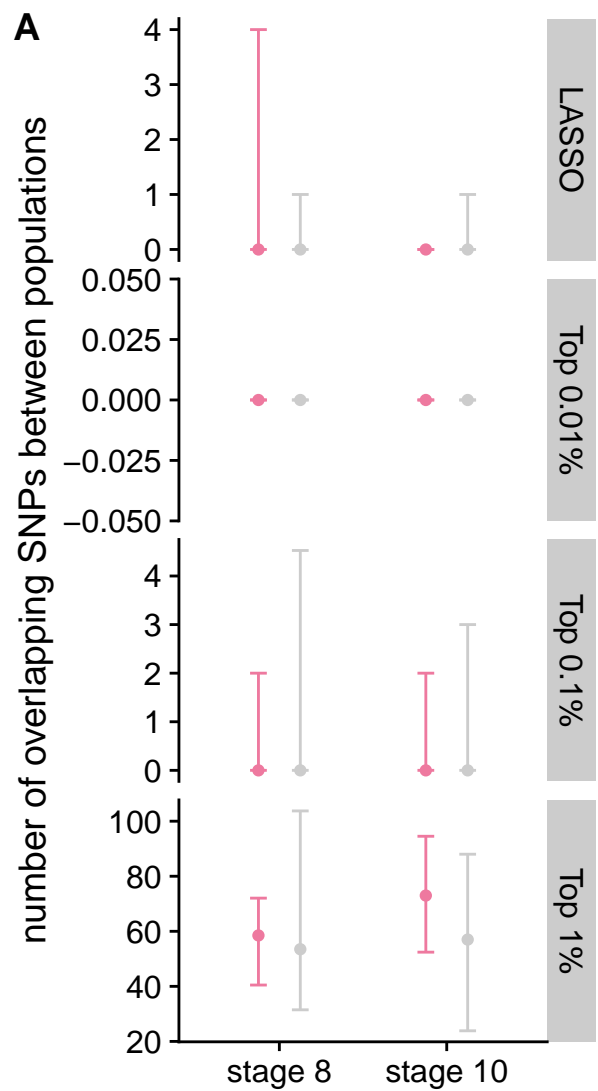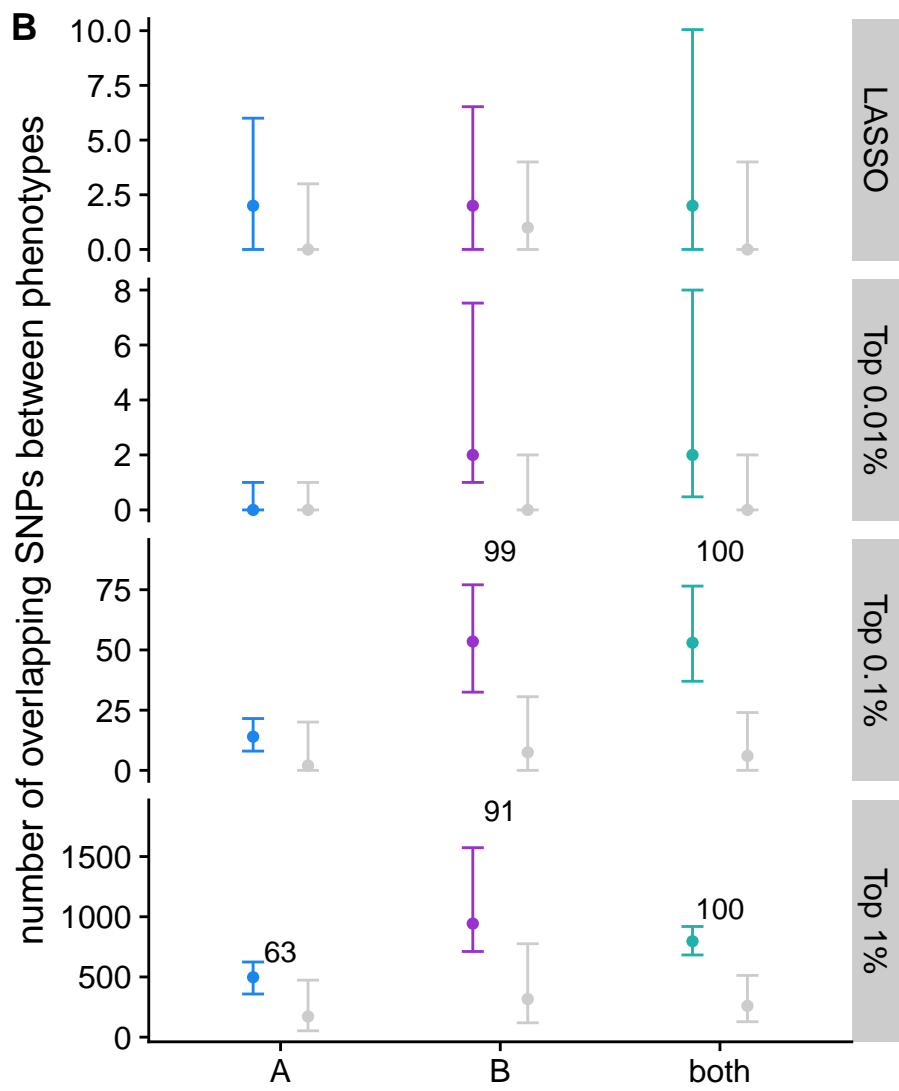

Supplement: S10 Fig — (A). For each imputation and permutation, the number of SNPs shared between populations A and B was counted for various sets of GWAS SNPs. Pink indicates 100 imputations of the actual data, grey indicates 100 permutations. Points represent the median and error bars extend to the 2.5% and 97.5% quantiles. (B) For each imputation and permutation, the number of SNPs shared between stage 8 and stage 10 diapause was counted. Grey points represent 100 permutations of populations A and B; 1000 permutations of both populations combined. Colored points represent 100 imputations of the actual data. Numbers represent the percent of imputations that exceed the 97.5% quantile of the permutations, if that number is greater than 50%. (PDF) [file pgen.1009110.s010.pdf]

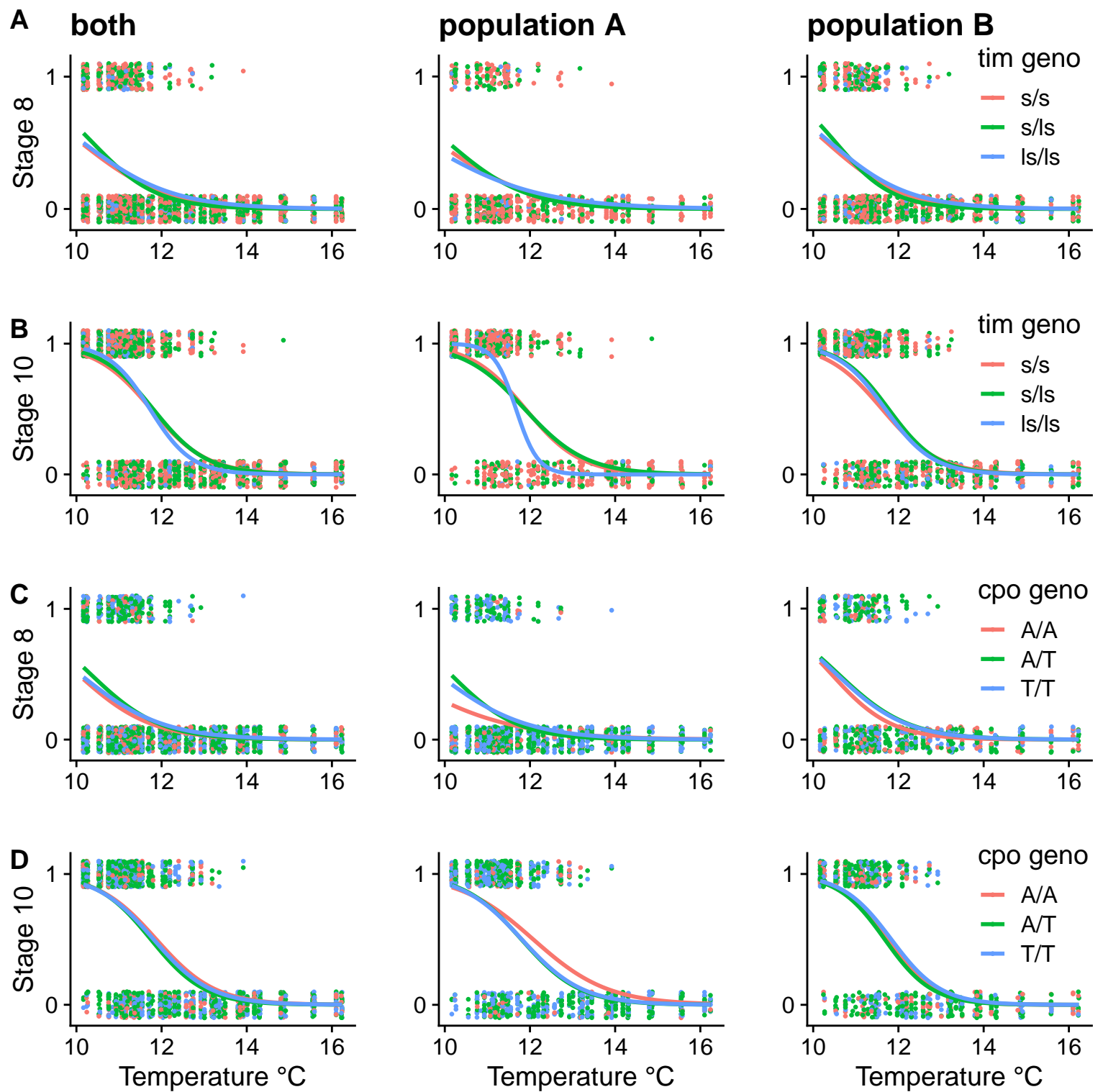

Supplement: S11 Fig — (A-B). A 1 bp indel that creates an alternate translation start site in timeless (tim) does not affect diapause in the full dataset or either individual population after correcting for temperature for diapause at stage 8 (A) or stage 10 (B). (C-D) An intronic SNP in couch potato (cpo) also has no effect in the full dataset or either individual population after correcting for temperature for diapause at stage 8 (C) or stage 10 (D). General linear model P-values for genotype are greater than 0.05 for all models, except for tim in population B, stage 8 (P = 0.0189). (PDF) [file pgen.1009110.s011.pdf]

**A****clinal**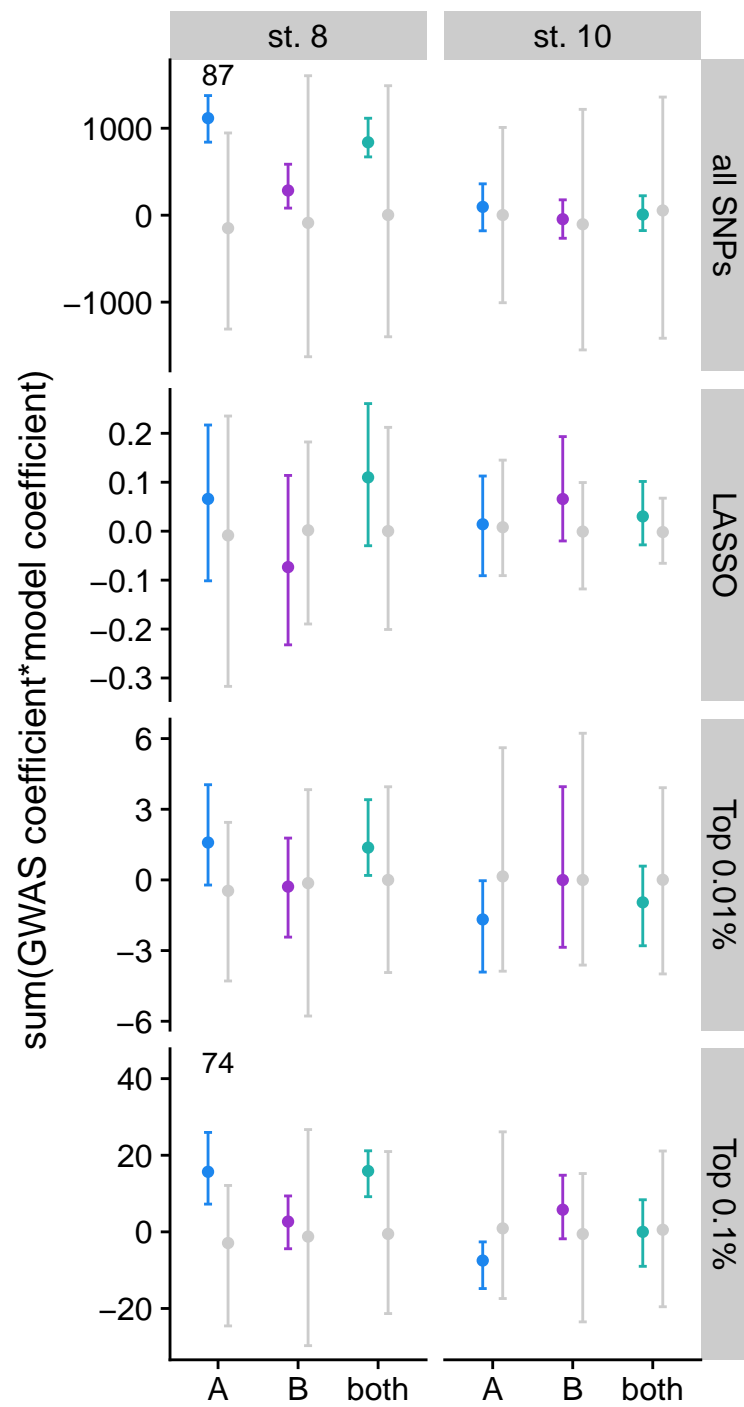**B****seasonal**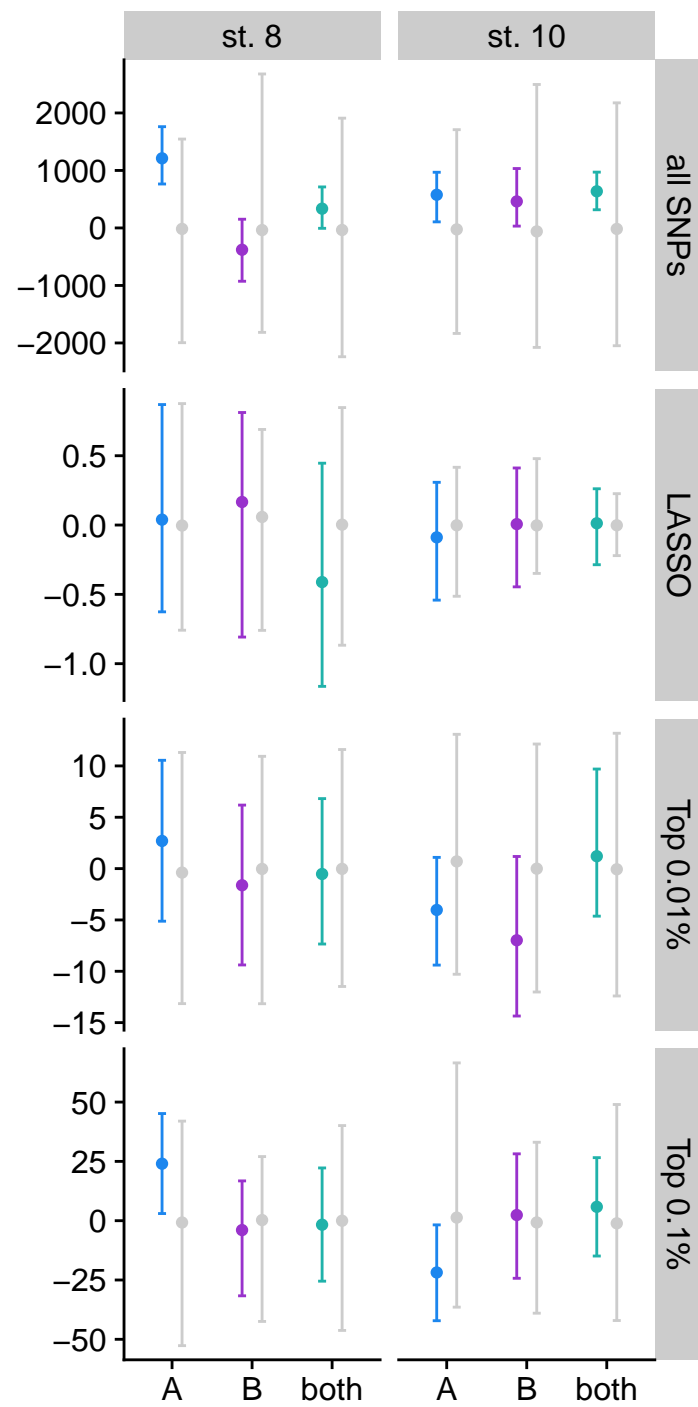

Supplement: S12 Fig — A) Polygenic scores calculated by multiplying clinal effect size and GWAS effect sizes for each SNP and summing across all SNPs, LASSO SNPs, the top 0.01% of the GWAS, and the top 0.1% of the GWAS. Effect sizes are polarized such that positive numbers indicated pro-diapause alleles are more common in the north. Data are shown with a point for the mean and error bars extending to the 2.5% and 97.5% quantiles. Colored points indicate actual data for 100 imputations of each mapping population; grey points indicate the distribution for permutations. B) Polygenic scores calculated for seasonal data by multiplying seasonal betas and GWAS effect sizes, polarized so that pro-diapause and spring are positive. Numbers represent the percent of imputations that exceed the 97.5% quantile of the permutations, if that number is greater than 50%. (PDF) [file pgen.1009110.s012.pdf]

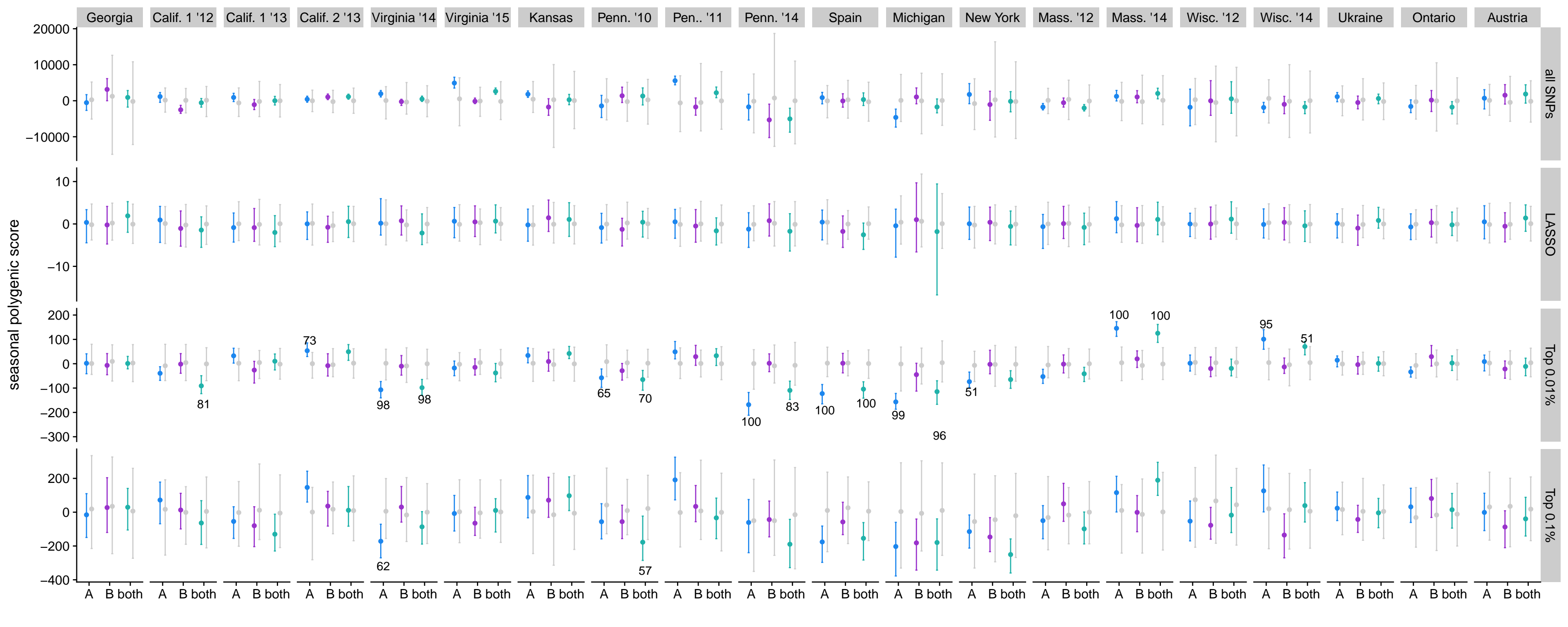

Supplement: S13 Fig — The GWAS or LASSO effect size was multiplied by the logit-transformed change in allele frequency from spring to fall in 20 populations. These products were then summed across all SNPs of interest for each mapping population. Populations are ordered by increasing latitude and year. Points represent median, error bars represent 2.5% and 97.5% quantiles. Grey points/bars are permutations; colors represent 100 imputations of the observed data. Numbers represent the percent of imputations that are below the 2.5% quantile or exceed the 97.5% quantile of the permutations, if that number is greater than 50%. (PDF) [file pgen.1009110.s013.pdf]

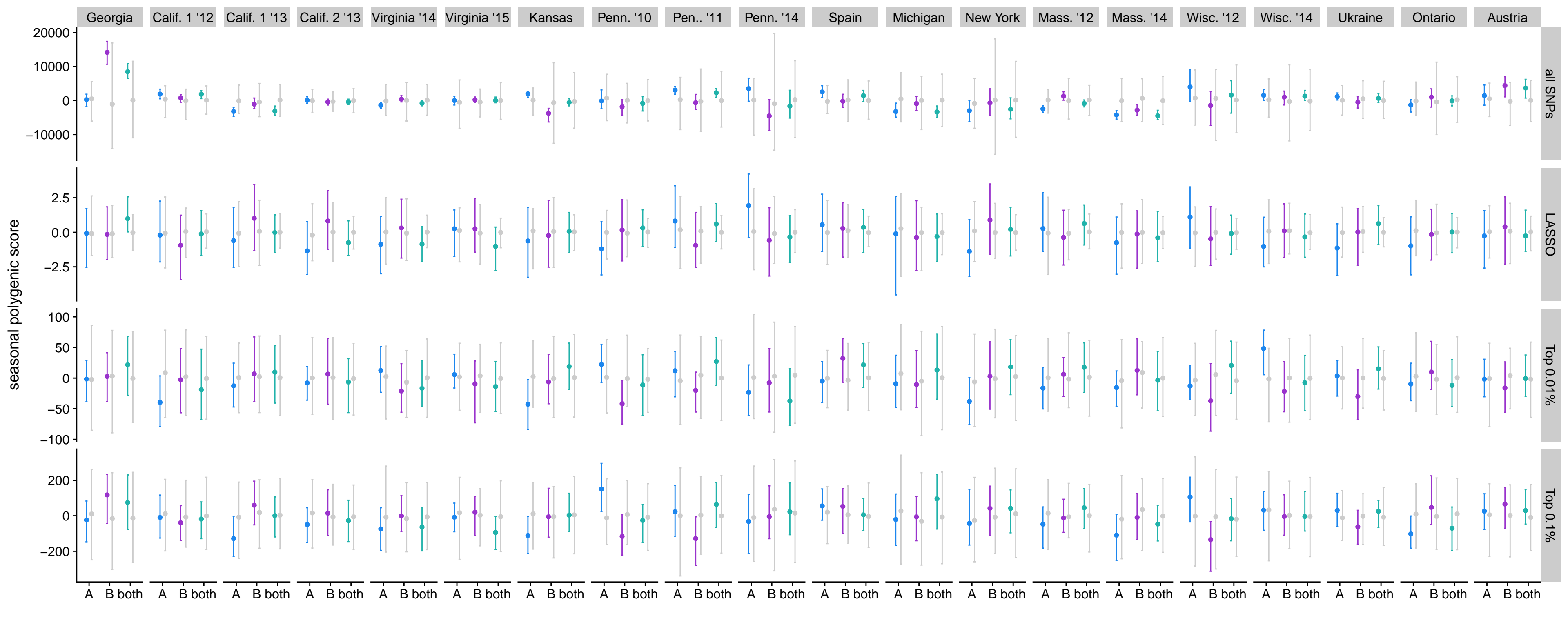

Supplement: S14 Fig — The GWAS or LASSO effect size was multiplied by the logit-transformed change in allele frequency from spring to fall in 20 populations. These products were then summed across all SNPs of interest for each mapping population. Populations are ordered by increasing latitude and year. Points represent median, error bars represent 2.5% and 97.5% quantiles. Grey points/bars are permutations; colors represent 100 imputations of the observed data. No imputations significantly exceed the 2.5% or 97.5% quantiles of the permutations. (PDF) [file pgen.1009110.s014.pdf]

**A**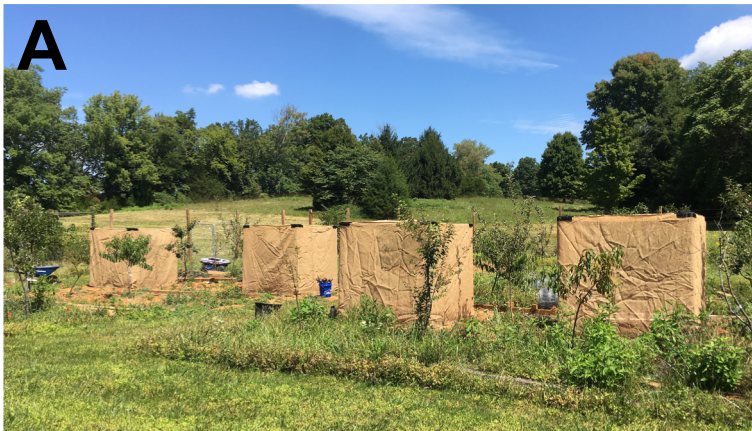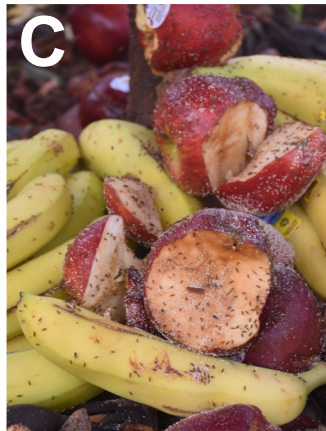**B**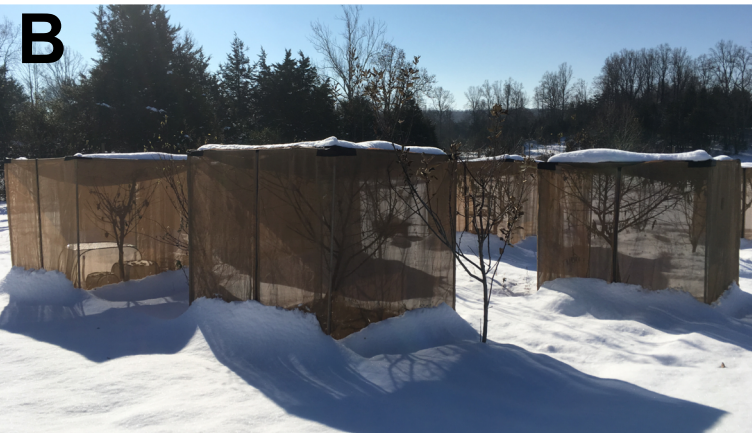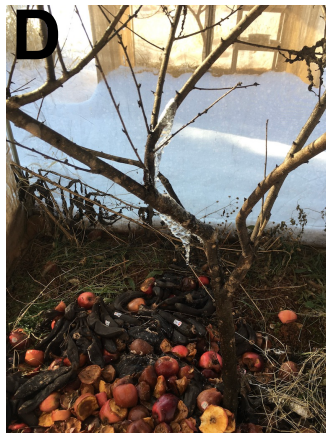

Supplement: S15 Fig — Summer (A) and winter (B) views of the experimental orchard with peach trees enclosed in mesh cages. (C) Flies feeding on yeasted fruit. (D) Winter view of compost pile in more advanced stage of decomposition. Photos in B and D were taken on December 10th, 2018, the final collection point in Fig 6. Surviving D. melanogaster were recovered from the cages on this day, despite heavy snow and several days of sub-freezing temperatures. (PDF) [file pgen.1009110.s015.pdf]

proportion of SNPs in admixture tracts

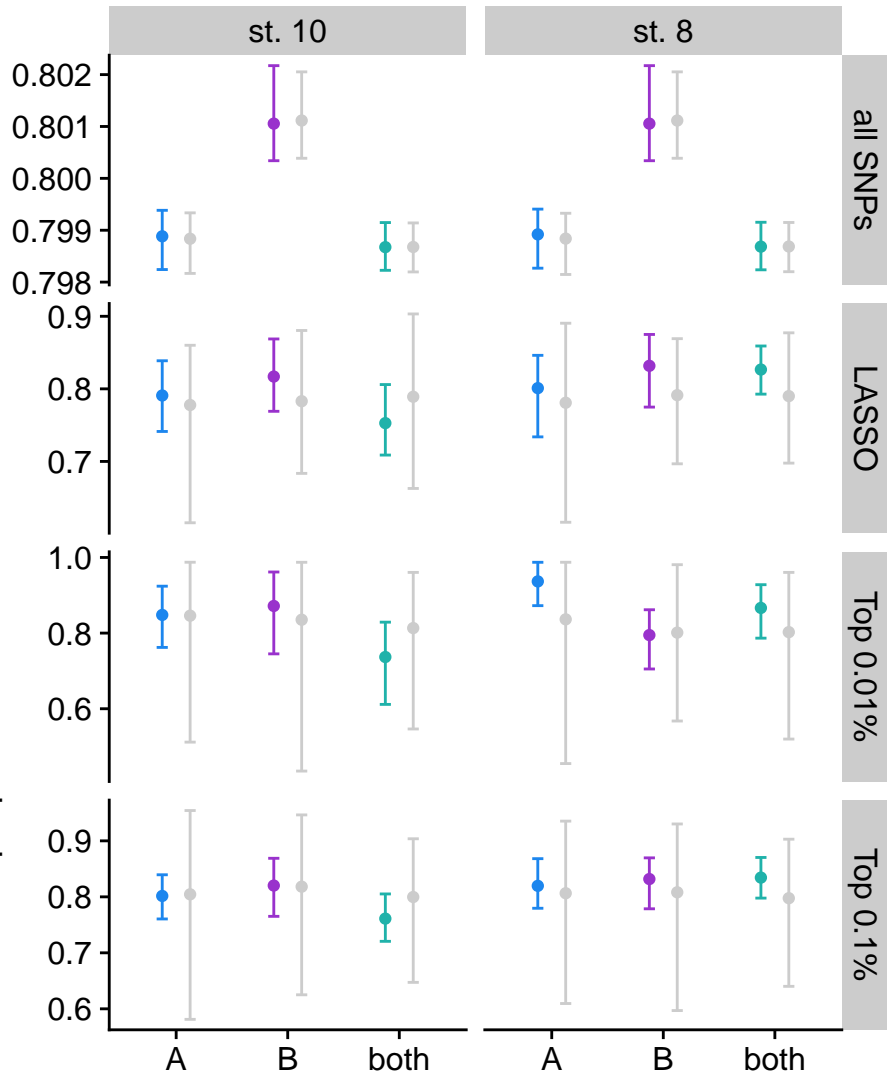

Supplement: S16 Fig — Each set of SNPs was intersected with the admixture tracts of European/Zambian admixture, and the proportion of SNPs found within at least one admixture tract was calculated for each mapping population. Points represent median, error bars represent 2.5% and 97.5% quantiles. No imputations significantly exceed the 2.5% or 97.5% quantiles of the permutations. Grey points/bars are permutations; colors represent 100 imputations of the observed data. (PDF) [file pgen.1009110.s016.pdf]

**A****clinal**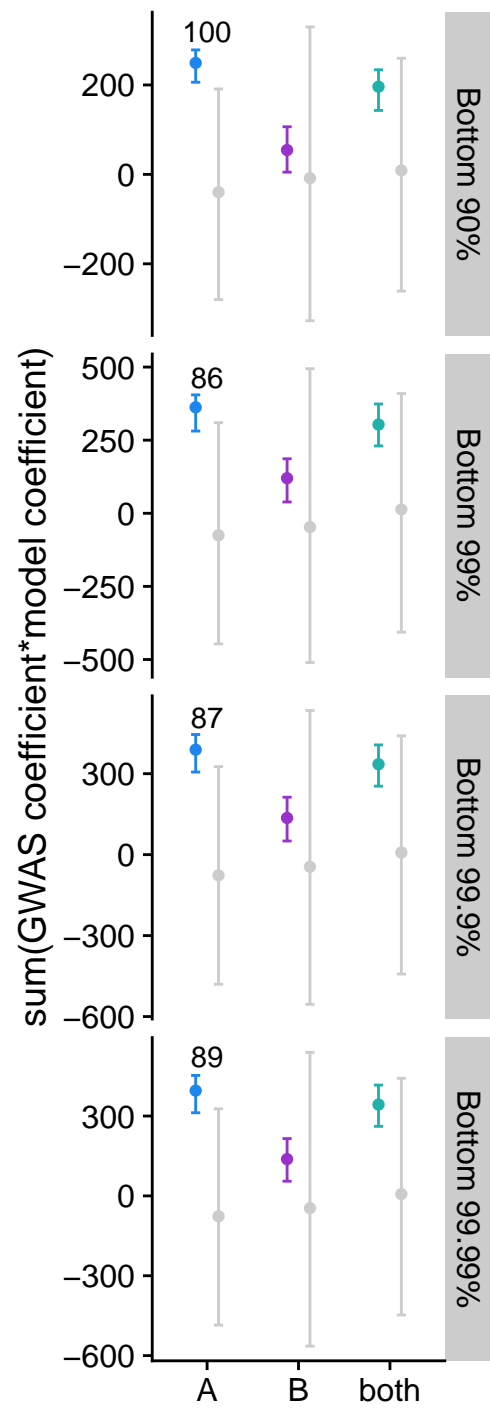**B****DGRP**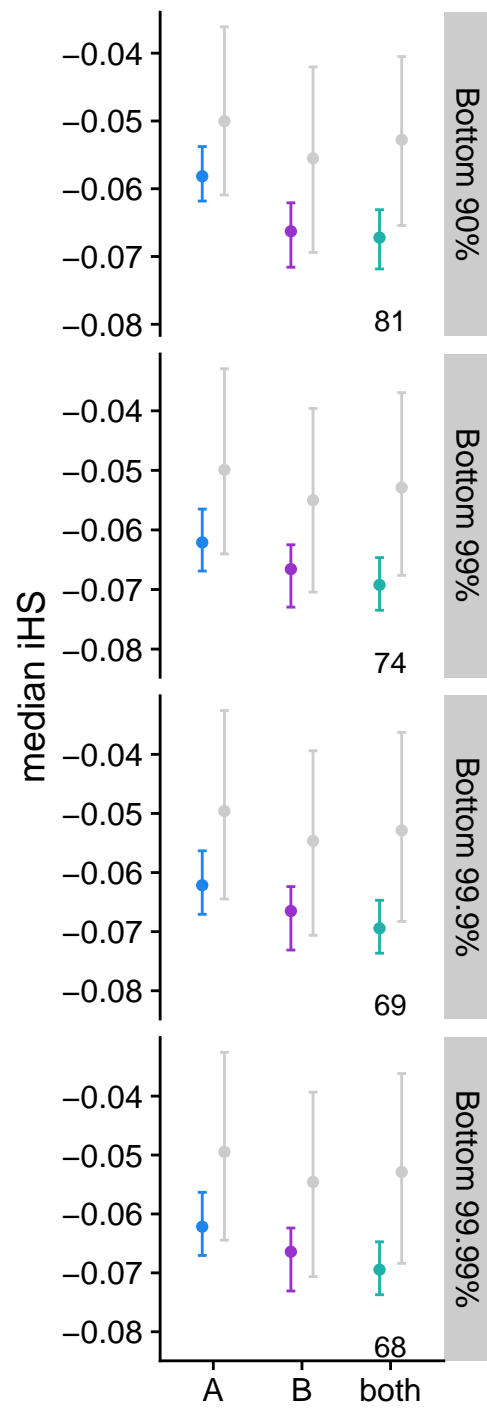**C****Northern**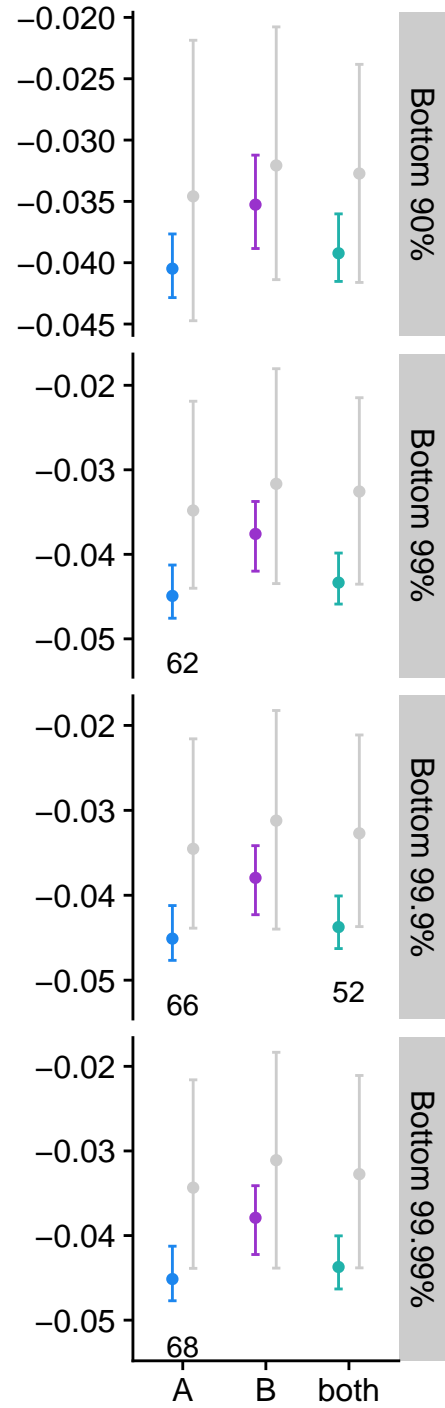

Supplement: S17 Fig — Top quantile-ranked SNPs were excluded from the analysis to determine whether they influence the genome-wide signal for clinal polygenic score based on Bergland et al 2014 (A), IHS in the DGRP (B) or IHS in Northern populations (C). Points represent median, error bars represent 2.5% and 97.5% quantiles. Grey points/bars are permutations; colors represent 100 imputations of the observed data. Numbers represent the percent of imputations that are below the 2.5% quantile or exceed the 97.5% quantile of the permutations, if that number is greater than 50%. (PDF) [file pgen.1009110.s017.pdf]

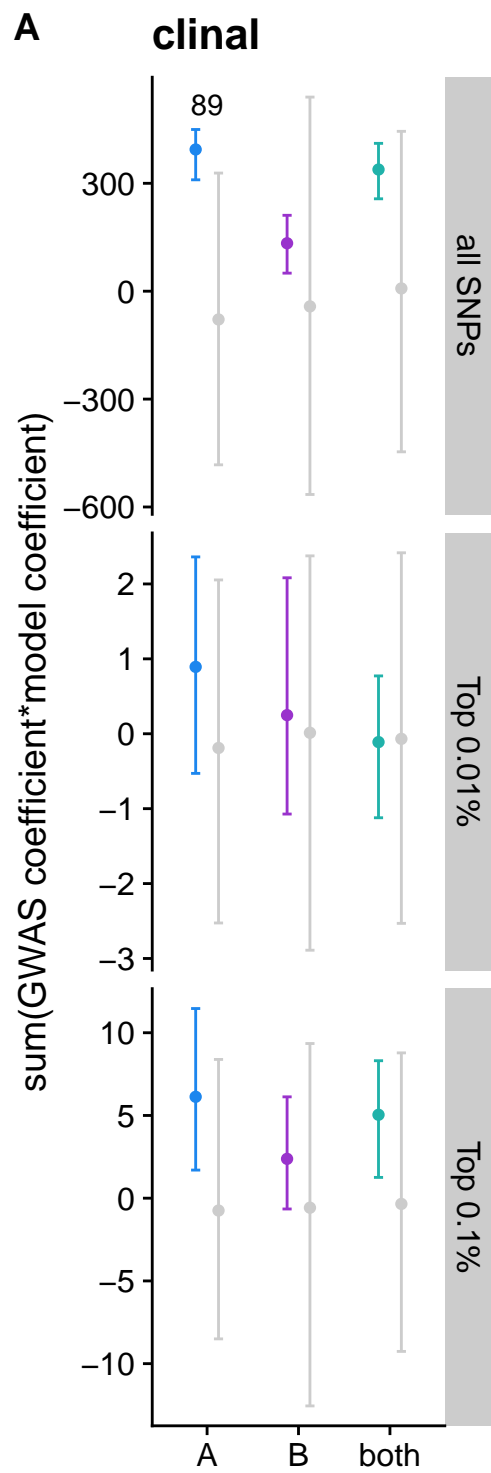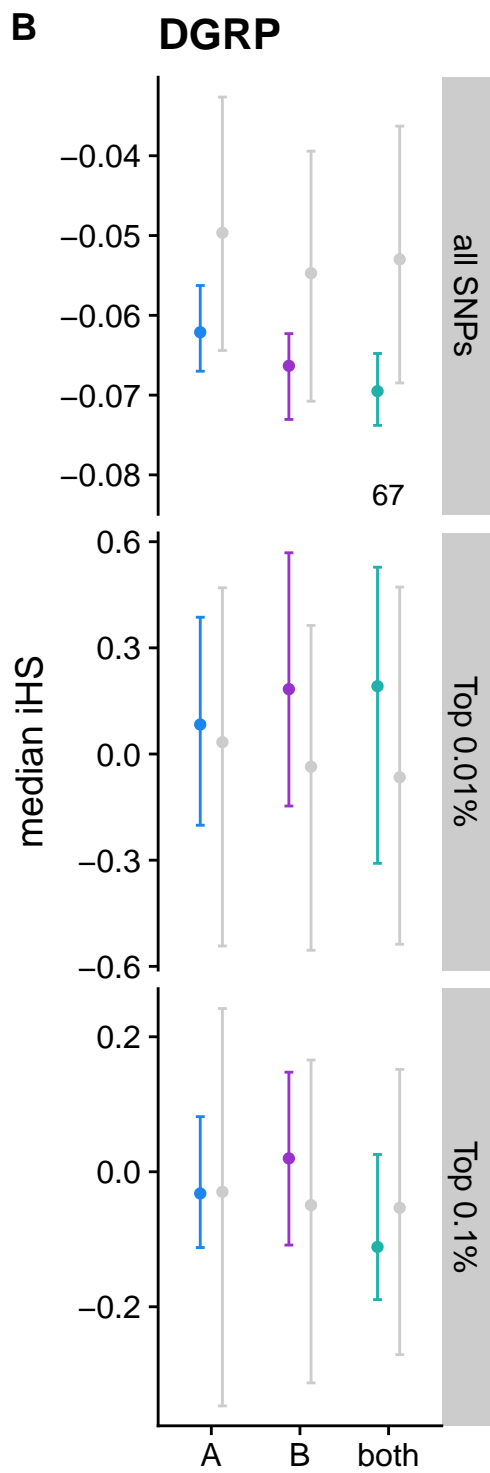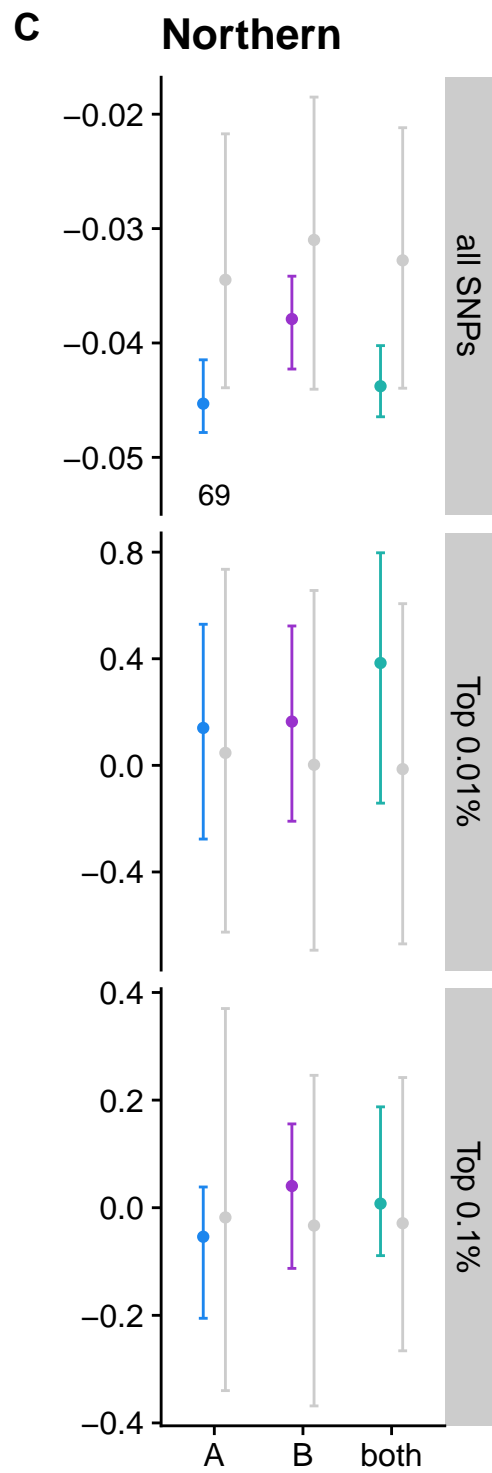

Supplement: S18 Fig — All SNPs in the region chrX:3,600,000–3,700,000 were excluded from the analysis, which is otherwise identical to that shown in Figs 6 and 9, for clinal polygenic score based on Bergland et al 2014 (A), IHS in the DGRP (B) or IHS in Northern populations (C). Points represent median, error bars represent 2.5% and 97.5% quantiles. Grey points/bars are permutations; colors represent 100 imputations of the observed data. Numbers represent the percent of imputations that are below the 2.5% quantile or exceed the 97.5% quantile of the permutations, if that number is greater than 50%. (PDF) [file pgen.1009110.s018.pdf]

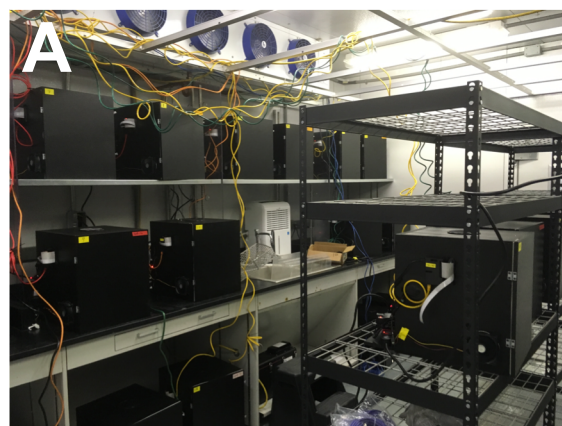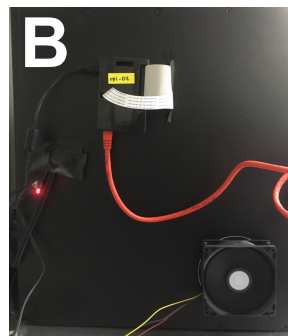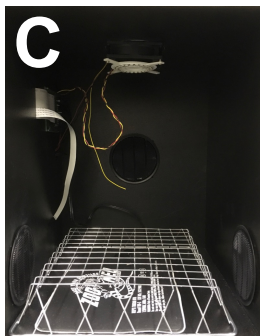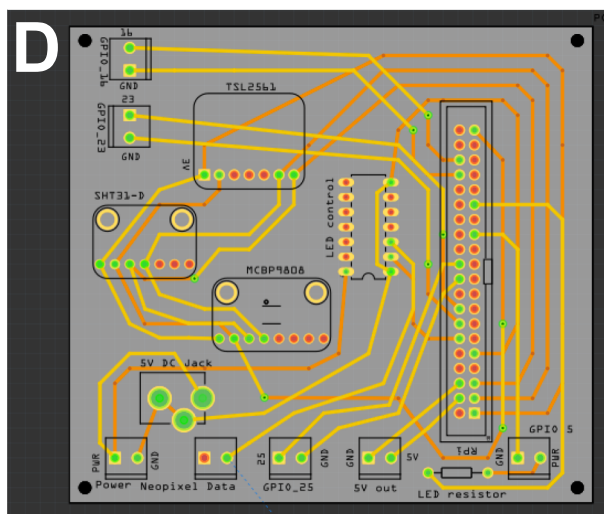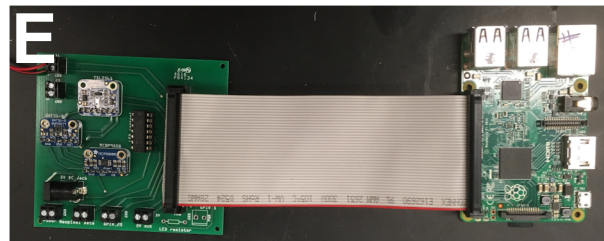

Supplement: S19 Fig — A) Array of chambers in cold room. B) Side view illustrating fan and externally mounted Raspberry Pi with ethernet connection. C) Interior view illustrating heating element (bottom), light-proof vents (sides), LED lights (top) and circuit board (top left). D) Fritzing layout for custom printed circuit board (PCB). File available upon request. E) Circuit board connected to Raspberry Pi computer via 40 pin ribbon cable. (PDF) [file pgen.1009110.s019.pdf]

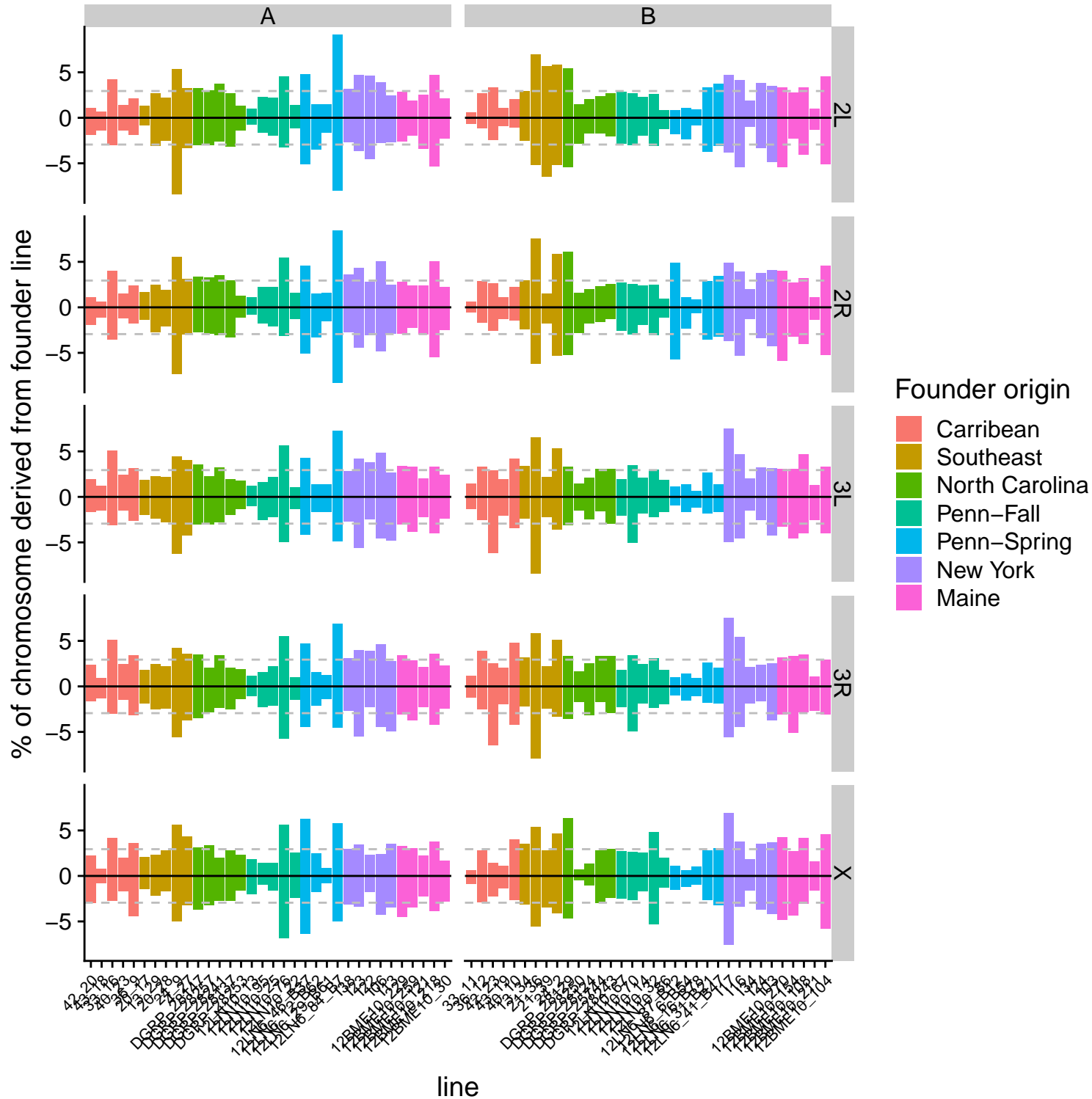

Supplement: S20 Fig — The percent contribution of each founding line in each chromosome arm was calculated for swarm A (left) and B (right). F4s are shown with positive values, and F5s are mirrored with negative values below. Color coding corresponds to geographical origin of the lines. Dashed grey lines indicate the expected contribution of each line (1/34 = 2.9%) under perfectly even admixture. (PDF) [file pgen.1009110.s020.pdf]

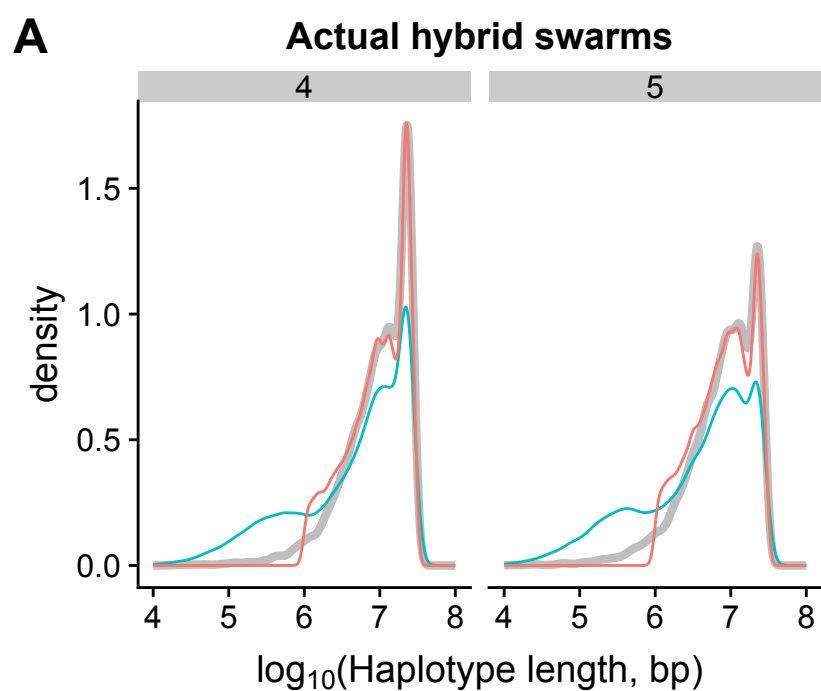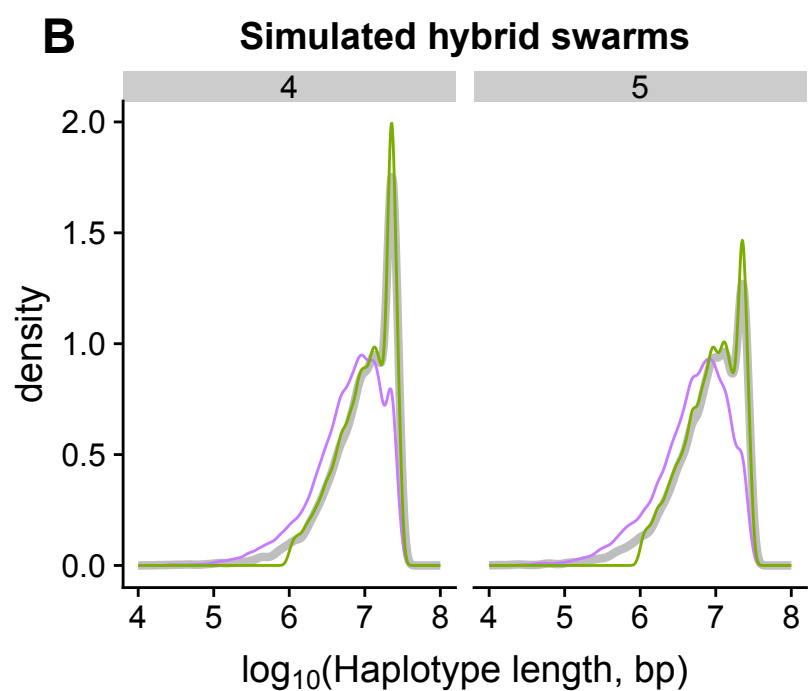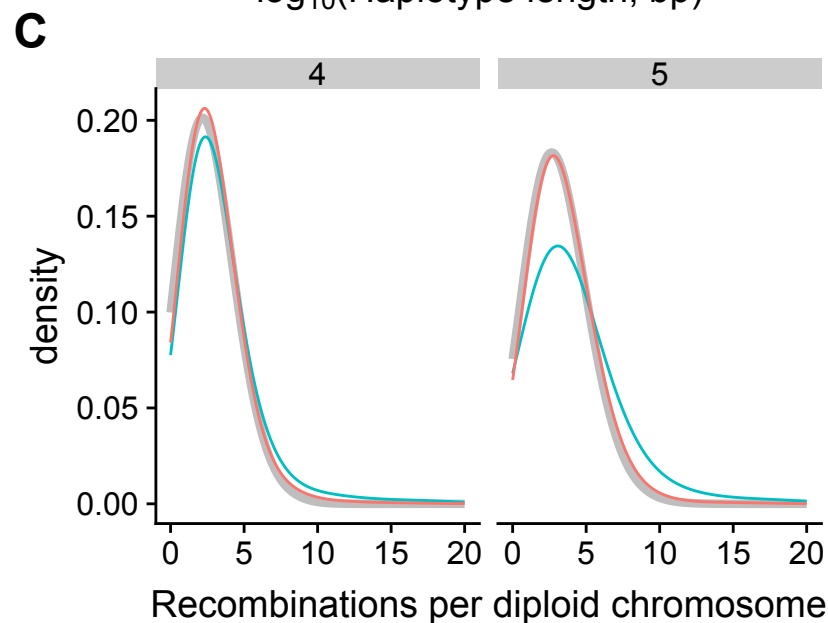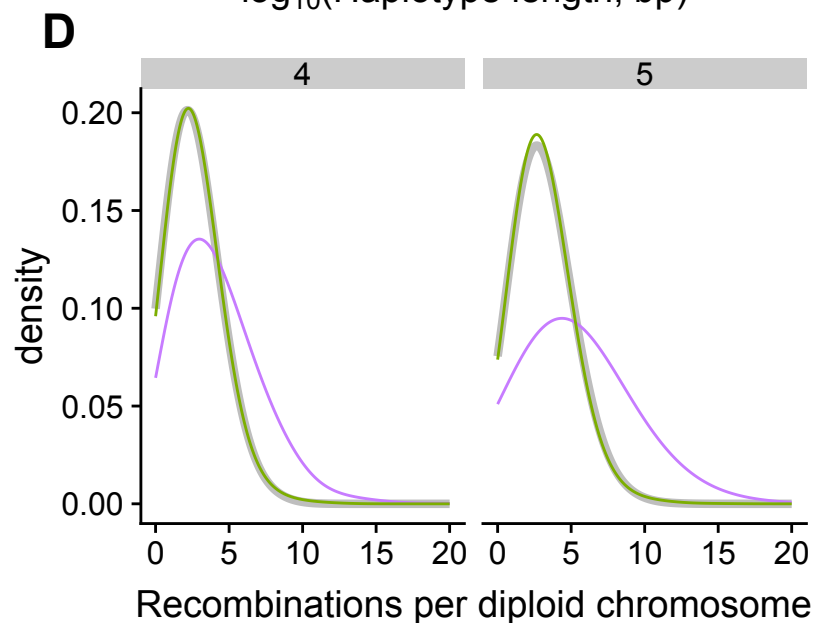

Cleaned up Reconstruction Simulation

Cleaned up Reconstruction Simulation

Supplement: S21 Fig — (A-B) The initial genome reconstruction of sequencing data (blue, A) shows an excess of short (~10,000–1,000,000 bp) haplotypes relative to simulated F4 and F5 populations (grey bold line). This excess also occurs in reconstructions of simulated hybrid swarm reads (B, purple). Cleaning up the reconstruction data by combining adjacent short (< 1 Mb) haplotypes into unknown haplotypes and dropping singleton short haplotypes results in a distribution of haplotype sizes that more closely match the simulations for both empirical data (red, A) and simulated data (green, B). (C-D) Raw reconstructions have an excess of recombination events (blue and purple) relative to simulated data (grey). The cleanup procedure results in recombination numbers on par with the simulated data (red and green). (PDF) [file pgen.1009110.s021.pdf]

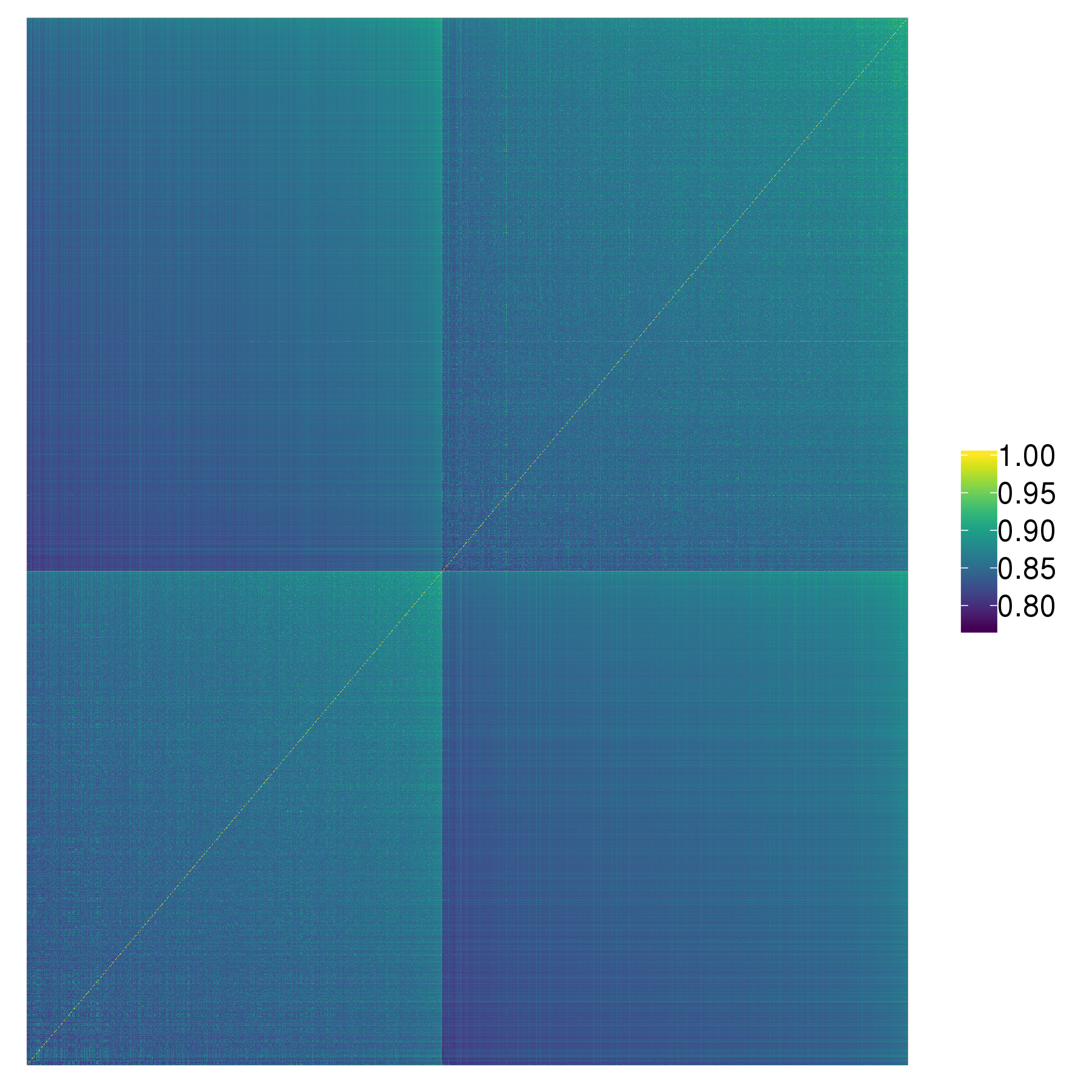

Supplement: S23 Fig — Individuals are ordered by population (A on left, B on right) and sorted by identity by state. IBS was calculated using an LD-pruned set of ~63,000 SNPs with allele frequencies > 0.05. Individuals are generally more closely related to other individuals in the same population. (TIF) [file pgen.1009110.s023.tif]
