# Supplementary material for: Unique genetic signatures of local adaptation over space and time for diapause, an ecologically relevant complex trait, in Drosophila melanogaster
Source: PLoS Genet. 2020 Nov 20;16(11):e1009110. doi: 10.1371/journal.pgen.1009110 (PMC7717581; doi:10.1371/journal.pgen.1009110)
Supplement: S22 Fig — Hybrid F4 and F5 individuals were simulated from the founding lines for populations A and B using a custom script, and 0.5X coverage sequencing reads were generated with wgsim. These simulated reads were passed through the genome reconstruction pipeline, and the reconstructed genotypes were compared to the original simulated individual. Accuracy was determined as the proportion of all sites with an exact match between the reconstructed genotype and actual genotype. The vast majority of individuals have an accuracy of >99%, though accuracy is higher in population A than population B. Note logarithmic scale of y-axis. (PDF) [file pgen.1009110.s022.pdf]

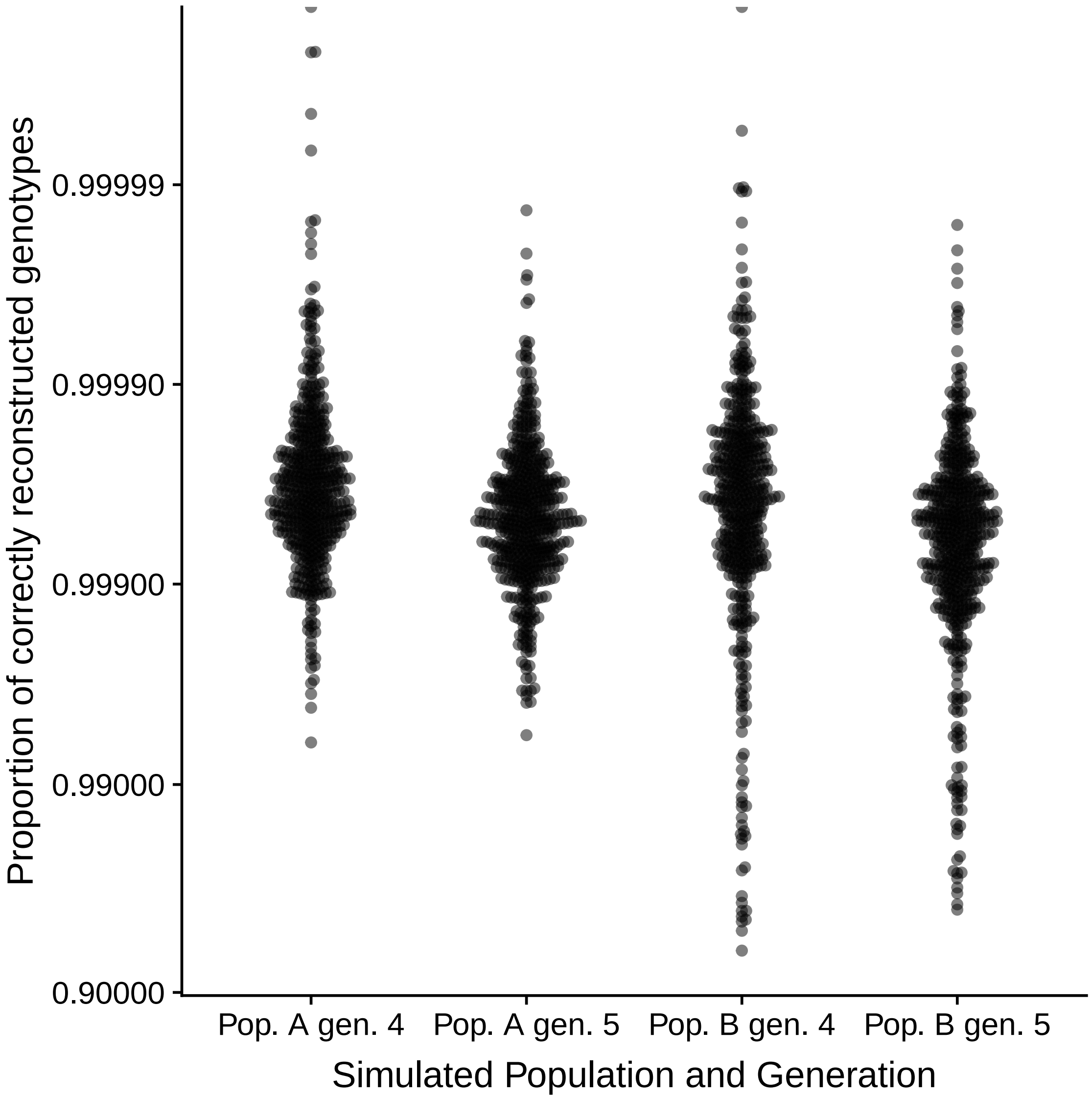
Proportion of correctly reconstructed genotypes
0.99999
0.99990
0.99900
0.99000
0.90000
Pop. A gen. 4
Pop. A gen. 5
Pop. B gen. 4
Pop. B gen. 5
Simulated Population and Generation
