# Supplementary material for: Unique genetic signatures of local adaptation over space and time for diapause, an ecologically relevant complex trait, in Drosophila melanogaster
Source: PLoS Genet. 2020 Nov 20;16(11):e1009110. doi: 10.1371/journal.pgen.1009110 (PMC7717581; doi:10.1371/journal.pgen.1009110)
Supplement: S2 Table — (PDF) [file pgen.1009110.s025.pdf]

	Stage 8				Stage 10			
	**Sum Sq**	**F**	**P**	**PVE**	**Sum Sq**	**F**	**P**	**PVE**
temperature	504.4	651.1	2.37E-129	**17.67**	1558.9	1676.2	8.55E-288	**33.84**
photoperiod	1.8	2.3	0.127	**0.06**	16.0	17.2	3.50E-05	**0.35**
generation	141.8	183.1	1.86E-40	**4.97**	416.0	447.3	3.04E-92	**9.03**
population	17.8	23.0	1.75E-06	**0.62**	0.8	0.9	0.34	**0.02**
Wolbachia	8.2	10.5	0.0011	**0.29**	0.4	0.5	0.49	**0.01**
temperature*photoperiod	3.6	4.7	0.03	**0.13**	0.7	0.7	0.39	**0.01**
residuals	2177.8	-	-	**76.27**	2613.3	-	-	**56.74**
