# Supplementary material for: Unique genetic signatures of local adaptation over space and time for diapause, an ecologically relevant complex trait, in Drosophila melanogaster
Source: PLoS Genet. 2020 Nov 20;16(11):e1009110. doi: 10.1371/journal.pgen.1009110 (PMC7717581; doi:10.1371/journal.pgen.1009110)
Supplement: S3 Table — (PDF) [file pgen.1009110.s026.pdf]

| | | | both | | A | | B | |
|---|---|---|---|---|---|---|---|---|
| | chr | inversion | stage 8 | stage 10 | stage 8 | stage 10 | stage 8 | stage 10 |
| 1 | 3R | Mo | 0.730 | 0.454 | 0.653 | 0.335 | 0.603 | 0.618 |
| 2 | 3R | C | 0.301 | 0.162 | 0.289 | 0.185 | NA | NA |
| 3 | 3R | Payne | **0.029** | **0.013** | **0.004** | **0.005** | 0.596 | 0.824 |
| 4 | 2L | t | 0.773 | 0.085 | 0.912 | 0.071 | 0.981 | 0.982 |
| 5 | 2R | Ns | 0.971 | 0.975 | 0.981 | 0.983 | NA | NA |

*Note: Bold text indicates P < 0.05; no inversions pass Bonferroni correction.*
