# Supplementary material for: Unique genetic signatures of local adaptation over space and time for diapause, an ecologically relevant complex trait, in Drosophila melanogaster
Source: PLoS Genet. 2020 Nov 20;16(11):e1009110. doi: 10.1371/journal.pgen.1009110 (PMC7717581; doi:10.1371/journal.pgen.1009110)
Supplement: S4 Table — For each class of variant, the percentage of diapause-associated SNPs assigned to that variant type was quantified. The percentage reported is the median of 100 imputations. (PDF) [file pgen.1009110.s027.pdf]

Annotation	Population	phenotype	Top 1%	Top 0.1%	Top 0.01%	LASSO
UTR	A	stage 8	6.3%	6.5%	10.1%	6.2%
	A	stage 10	6.4%	6.3%	2.6%	7.6%
	B	stage 8	6.7%	7.6%	11.5%	5.0%
	B	stage 10	6.2%	5.2%	2.6%	5.1%
	both	stage 8	6.4%	6.6%	10.5%	5.9%
	both	stage 10	6.2%	5.2%	3.9%	4.6%
intergenic	A	stage 8	11.2%	11.8%	3.8%	10.4%
	A	stage 10	10.3%	10.9%	13.9%	9.8%
	B	stage 8	11.2%	11.0%	10.3%	11.4%
	B	stage 10	11.4%	8.9%	5.1%	9.4%
	both	stage 8	10.0%	7.0%	3.9%	9.8%
	both	stage 10	10.8%	8.4%	3.3%	7.2%
intronic	A	stage 8	25.4%	24.2%	12.7%	23.7%
	A	stage 10	24.6%	24.0%	34.6%	23.1%
	B	stage 8	23.4%	24.3%	22.8%	25.3%
	B	stage 10	27.2%	31.7%	36.2%	24.8%
	both	stage 8	25.2%	24.6%	17.3%	27.2%
	both	stage 10	25.7%	30.2%	35.3%	31.4%
non-synonymous	A	stage 8	4.3%	3.7%	0.0%	3.5%
	A	stage 10	4.2%	3.8%	1.3%	3.1%
	B	stage 8	4.3%	4.2%	5.1%	3.1%
	B	stage 10	3.9%	3.0%	3.8%	3.7%
	both	stage 8	3.9%	3.5%	3.9%	3.3%
	both	stage 10	3.7%	3.2%	2.7%	3.0%
synonymous	A	stage 8	11.0%	10.5%	11.5%	12.8%
	A	stage 10	11.6%	11.9%	7.6%	13.1%
	B	stage 8	10.1%	10.0%	8.9%	12.1%
	B	stage 10	8.6%	7.4%	7.7%	9.7%
	both	stage 8	10.6%	10.0%	9.3%	10.9%
	both	stage 10	10.2%	8.7%	5.3%	10.5%
upstream/downstream	A	stage 8	41.6%	42.8%	60.3%	42.4%
	A	stage 10	42.7%	42.9%	39.1%	43.3%
	B	stage 8	44.2%	42.9%	42.3%	42.7%
	B	stage 10	42.6%	43.2%	43.9%	46.7%
	both	stage 8	43.8%	48.2%	54.2%	42.7%
	both	stage 10	43.2%	44.3%	48.7%	43.1%
