# Supplementary material for: Unique genetic signatures of local adaptation over space and time for diapause, an ecologically relevant complex trait, in Drosophila melanogaster
Source: PLoS Genet. 2020 Nov 20;16(11):e1009110. doi: 10.1371/journal.pgen.1009110 (PMC7717581; doi:10.1371/journal.pgen.1009110)
Supplement: S5 Table — For each class of variant, the proportion of diapause-associated SNPs assigned to that variant type was quantified for each imputation and permutation. The numerical values are the quantile rank of the median of the 100 observed imputations relative to the distribution of the permutations. Quantile ranks below 5% and above 95% (i.e. those values that are de-enriched or enriched relative to permutations) are highlighted in bold. (PDF) [file pgen.1009110.s028.pdf]

Annotation	Population	phenotype	Top 1%	Top 0.1%	Top 0.01%	LASSO
UTR	A	stage 8	0.55	0.66	0.94	0.59
	A	stage 10	0.68	0.55	0.23	0.74
	B	stage 8	0.9	0.89	**0.96**	0.26
	B	stage 10	0.52	0.28	0.26	0.4
	both	stage 8	0.767	0.687	0.897	0.467
	both	stage 10	0.533	0.214	0.282	0.297
intergenic	A	stage 8	0.69	0.75	0.09	0.46
	A	stage 10	0.3	0.52	0.73	0.54
	B	stage 8	0.69	0.59	0.59	0.59
	B	stage 10	0.77	0.24	0.23	0.32
	both	stage 8	0.224	0.041	0.104	0.321
	both	stage 10	0.505	0.147	0.062	0.155
intronic	A	stage 8	0.78	0.51	0.1	0.36
	A	stage 10	0.57	0.5	0.89	0.33
	B	stage 8	0.22	0.48	0.44	0.56
	B	stage 10	0.97	0.97	0.87	0.43
	both	stage 8	0.706	0.515	0.203	0.747
	both	stage 10	0.832	0.963	0.905	0.9
non-synonymous	A	stage 8	0.6	0.38	0.04	0.55
	A	stage 10	0.5	0.33	0.23	0.31
	B	stage 8	0.84	0.59	0.79	0.38
	B	stage 10	0.23	0.15	0.59	0.44
	both	stage 8	0.403	0.331	0.582	0.378
	both	stage 10	0.244	0.193	0.453	0.367
synonymous	A	stage 8	0.18	0.31	0.59	0.69
	A	stage 10	0.61	0.62	0.29	0.6
	B	stage 8	0.08	0.17	0.3	0.57
	B	stage 10	**0**	**0**	0.32	0.35
	both	stage 8	0.083	0.205	0.415	0.376
	both	stage 10	**0.019**	0.043	0.072	0.396
upstream/downstream	A	stage 8	0.1	0.48	**0.98**	0.52
	A	stage 10	0.47	0.47	0.36	0.6
	B	stage 8	0.84	0.46	0.44	0.58
	B	stage 10	0.4	0.61	0.52	0.83
	both	stage 8	0.811	0.954	0.904	0.529
	both	stage 10	0.632	0.706	0.786	0.552
